# Supplementary material for: World Health Organization Danger Signs to predict bacterial sepsis in young infants: A pragmatic cohort study
Source: PLOS Glob Public Health. 2023 Nov 21;3(11):e0001990. doi: 10.1371/journal.pgph.0001990 (PMC10662722; doi:10.1371/journal.pgph.0001990)
Supplement: S1 Table — *Bacteria were also cultured from cerebrospinal fluid (CSF). (DOCX) [file pgph.0001990.s001.docx]

**S1 Table:** Bacterial isolates categorized as pathogens or potential contaminants in secondary analysis using the modified bacterial sepsis outcome.

| Pathogens | N = 41 |
| --- | --- |
| Gram-positive bacteria | N = 21 |
| Staphylococcus aureus | 11 |
| Enterococcus faecium | 5 |
| Streptococcus agalactiae | 2 |
| Streptococcus pyogenes | 1 |
| Group D streptococcus | 1 |
| Streptococcus pneumoniae | 1 |
|  |  |
| Gram-negative bacteria | N = 20 |
| Acinetobacter baumannii | 5 |
| Klebsiella pneumoniae* | 6 |
| Citrobacter freundii | 2 |
| Escherichia coli | 2 |
| Pantoea calida | 2 |
| Pseudomonas aeruginosa/monteilii | 1 |
| Salmonella spp | 1 |
| Unidentified gram-negative diplococci* | 1 |
|  |  |
| Contaminants | N = 46 |
| Gram-positive bacteria | N = 46 |
| Coagulase-negative staphylococcus spp | 27 |
| Bacillus spp | 5 |
| Micrococcus spp | 4 |
| Staphylococcus haemolyticus | 4 |
| Corynebacterium spp | 3 |
| Streptococcus spp (including one *S.* *oralis*) | 3 |

*Bacteria were also cultured from cerebrospinal fluid (CSF)
