# Supplementary material for: World Health Organization Danger Signs to predict bacterial sepsis in young infants: A pragmatic cohort study
Source: PLOS Glob Public Health. 2023 Nov 21;3(11):e0001990. doi: 10.1371/journal.pgph.0001990 (PMC10662722; doi:10.1371/journal.pgph.0001990)
Supplement: S2 Table — £adjusted OR for age of mother, gestational age, birth and admission weights, age of the infant and sex; *Significant ORs (p<0.05). (DOCX) [file pgph.0001990.s002.docx]

**S2 Table:** Baseline infant characteristics associated with increased mortality.

|  | Cumulative number of DS | | | Mean number of DS | | |
| --- | --- | --- | --- | --- | --- | --- |
| Characteristic | OR | 95%CI | P value | OR | 95%CI | P value |
| Number of mean DS^£^ | 1.75 | (1.39 - 2.23) | <0.001 | 1.80 | (1.44 - 2.30) | <0.001* |
| Age of mother (years) | 0.98 | (0.91 - 1.05) | 0.532 | 0.98 | (0.91 -1.05) | 0.578 |
| Gestational age (weeks) | 1.08 | (0.89 - 1.31) | 0.449 | 1.08 | (0.89 - 1.31) | 0.454 |
| Birth weight (grams) | 1.00 | (0.99 – 1.00) | 0.761 | 1.00 | (0.99 – 1.00) | 0.746 |
| Admission weight (grams) | 1.00 | (0.99 – 1.00) | 0.044 | 1.00 | (0.99 – 1.00) | 0.040* |
| Age of infant (days) | 0.99 | (0.95 - 1.03) | 0.777 | 1.00 | (0.95 - 1.03) | 0.810 |
| Male sex | 1.86 | (0.84 – 4.34) | 0.138 | 1.83 | (0.82 – 4.30) | 0.151 |

^£^adjusted OR for age of mother, gestational age, birth and admission weights, age of the infant and sex

*Significant ORs (p<0.05)
