# Supplementary material for: World Health Organization Danger Signs to predict bacterial sepsis in young infants: A pragmatic cohort study
Source: PLOS Glob Public Health. 2023 Nov 21;3(11):e0001990. doi: 10.1371/journal.pgph.0001990 (PMC10662722; doi:10.1371/journal.pgph.0001990)
Supplement: S3 Table — (DOCX) [file pgph.0001990.s003.docx]

**S3 Table:** Associations between DS and bacterial sepsis when all organisms are included (i.e., including both pathogens and potential contaminants).

| Variable | Odds ratio (OR) | 95%CI | P value |
| --- | --- | --- | --- |
| Not feeding well | 1.43 | (0.88 - 2.32) | 0.151 |
| Convulsions | 0.79 | (0.26 – 1.98) | 0.640 |
| Drowsy / Difficult to wake | 1.20 | (0.49 - 2.65) | 0.673 |
| Movements only when stimulated or no movements | 1.11 | (0.40 - 2.71) | 0.825 |
| Fast breathing >=60 per min | 1.04 | (0.60 - 1.78) | 0.884 |
| Grunting | 1.95 | (0.85 - 4.26) | 0.101 |
| Severe chest recessions | 1.89 | (0.97 - 3.58) | 0.055 |
| Temperature instability >38°C or <35.5°C | 0.75 | (0.43 - 1.29) | 0.313 |
| Central cyanosis | 1.19 | (0.46 - 2.75) | 0.705 |
