# Supplementary material for: World Health Organization Danger Signs to predict bacterial sepsis in young infants: A pragmatic cohort study
Source: PLOS Glob Public Health. 2023 Nov 21;3(11):e0001990. doi: 10.1371/journal.pgph.0001990 (PMC10662722; doi:10.1371/journal.pgph.0001990)
Supplement: S1 Fig — Plots of model fits—Death as outcome. (DOCX) [file pgph.0001990.s004.docx]

## S1 Fig: Relationship between DS and mortality.

## Plots of model fits - Death as outcome


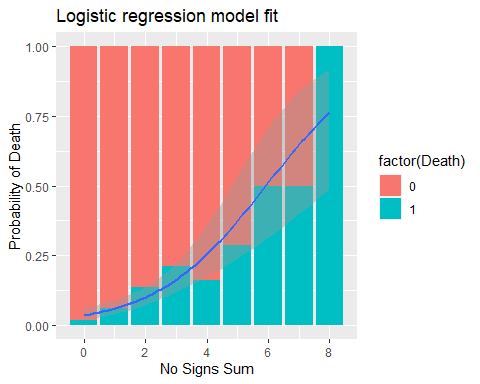


Corresponding data:

| Number DS | Factor (death) | n | fit | fit.lc | fit.uc |
| --- | --- | --- | --- | --- | --- |
| 0 | 0 | 104 | 0.0351417 | 0.0147551 | 0.0555283 |
| 0 | 1 | 2 | 0.0351417 | 0.0147551 | 0.0555283 |
| 1 | 0 | 127 | 0.0599392 | 0.0346367 | 0.0852418 |
| 1 | 1 | 8 | 0.0599392 | 0.0346367 | 0.0852418 |
| 2 | 0 | 70 | 0.1004140 | 0.0681221 | 0.1327059 |
| 2 | 1 | 11 | 0.1004140 | 0.0681221 | 0.1327059 |
| 3 | 0 | 30 | 0.1634673 | 0.1123113 | 0.2146234 |
| 3 | 1 | 8 | 0.1634673 | 0.1123113 | 0.2146234 |
| 4 | 0 | 21 | 0.2548952 | 0.1622197 | 0.3475708 |
| 4 | 1 | 4 | 0.2548952 | 0.1622197 | 0.3475708 |
| 5 | 0 | 5 | 0.3745627 | 0.2223311 | 0.5267942 |
| 5 | 1 | 2 | 0.3745627 | 0.2223311 | 0.5267942 |
| 6 | 0 | 1 | 0.5118198 | 0.3039031 | 0.7197366 |
| 6 | 1 | 1 | 0.5118198 | 0.3039031 | 0.7197366 |
| 7 | 0 | 1 | 0.6473174 | 0.4135357 | 0.8810992 |
| 7 | 1 | 1 | 0.6473174 | 0.4135357 | 0.8810992 |
| 8 | 1 | 2 | 0.7626473 | 0.5416290 | 0.9836657 |
