# Supplementary material for: World Health Organization Danger Signs to predict bacterial sepsis in young infants: A pragmatic cohort study
Source: PLOS Glob Public Health. 2023 Nov 21;3(11):e0001990. doi: 10.1371/journal.pgph.0001990 (PMC10662722; doi:10.1371/journal.pgph.0001990)
Supplement: S2 Fig — Plots of model fits—pathogen sepsis as outcome. (DOCX) [file pgph.0001990.s005.docx]

**S2 Fig:** Relationship between DS and pathogen bacterial sepsis.

## Plots of model fits - pathogen sepsis as outcome


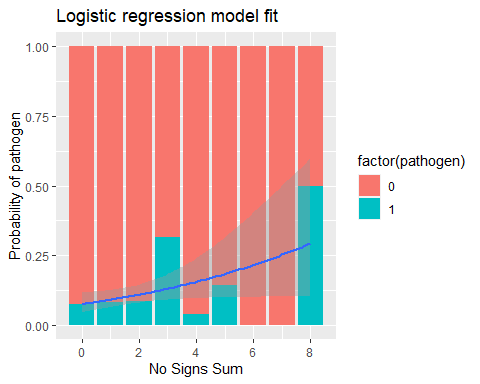


**Corresponding data:**

| Number DS | Factor (pathogen) | n | fit | fit.lc | fit.uc |
| --- | --- | --- | --- | --- | --- |
| 0 | 0 | 98 | 0.0759791 | 0.0417148 | 0.1102434 |
| 0 | 1 | 8 | 0.0759791 | 0.0417148 | 0.1102434 |
| 1 | 0 | 124 | 0.0914780 | 0.0611961 | 0.1217600 |
| 1 | 1 | 11 | 0.0914780 | 0.0611961 | 0.1217600 |
| 2 | 0 | 74 | 0.1097630 | 0.0778348 | 0.1416912 |
| 2 | 1 | 7 | 0.1097630 | 0.0778348 | 0.1416912 |
| 3 | 0 | 26 | 0.1311751 | 0.0855578 | 0.1767925 |
| 3 | 1 | 12 | 0.1311751 | 0.0855578 | 0.1767925 |
| 4 | 0 | 24 | 0.1560322 | 0.0847390 | 0.2273253 |
| 4 | 1 | 1 | 0.1560322 | 0.0847390 | 0.2273253 |
| 5 | 0 | 6 | 0.1845987 | 0.0775872 | 0.2916102 |
| 5 | 1 | 1 | 0.1845987 | 0.0775872 | 0.2916102 |
| 6 | 0 | 2 | 0.2170502 | 0.0653179 | 0.3687825 |
| 7 | 0 | 2 | 0.2534333 | 0.0489157 | 0.4579510 |
| 8 | 0 | 1 | 0.2936281 | 0.0297133 | 0.5575428 |
| 8 | 1 | 1 | 0.2936281 | 0.0297133 | 0.5575428 |
