# Supplementary material for: World Health Organization Danger Signs to predict bacterial sepsis in young infants: A pragmatic cohort study
Source: PLOS Glob Public Health. 2023 Nov 21;3(11):e0001990. doi: 10.1371/journal.pgph.0001990 (PMC10662722; doi:10.1371/journal.pgph.0001990)
Supplement: S3 Fig — Plots of model fits–All sepsis as outcome. (DOCX) [file pgph.0001990.s006.docx]

**S3 Fig:** Relationship between DS and bacterial sepsis including contaminants.

## Plots of model fits – All sepsis as outcome


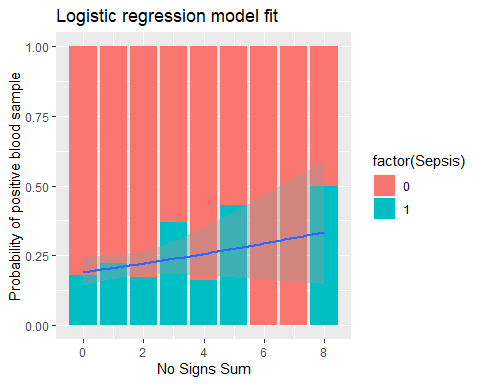


**Corresponding data:**

| Number DS | Factor (sepsis) | n | fit | fit.lc | fit.uc |
| --- | --- | --- | --- | --- | --- |
| 0 | 0 | 87 | 0.1902327 | 0.1362013 | 0.2442642 |
| 0 | 1 | 19 | 0.1902327 | 0.1362013 | 0.2442642 |
| 1 | 0 | 105 | 0.2052381 | 0.1631518 | 0.2473244 |
| 1 | 1 | 30 | 0.2052381 | 0.1631518 | 0.2473244 |
| 2 | 0 | 67 | 0.2211039 | 0.1781752 | 0.2640325 |
| 2 | 1 | 14 | 0.2211039 | 0.1781752 | 0.2640325 |
| 3 | 0 | 24 | 0.2378291 | 0.1777866 | 0.2978716 |
| 3 | 1 | 14 | 0.2378291 | 0.1777866 | 0.2978716 |
| 4 | 0 | 21 | 0.2554047 | 0.1683972 | 0.3424122 |
| 4 | 1 | 4 | 0.2554047 | 0.1683972 | 0.3424122 |
| 5 | 0 | 4 | 0.2738125 | 0.1543526 | 0.3932723 |
| 5 | 1 | 3 | 0.2738125 | 0.1543526 | 0.3932723 |
| 6 | 0 | 2 | 0.2930249 | 0.1373661 | 0.4486836 |
| 7 | 0 | 2 | 0.3130043 | 0.1182547 | 0.5077538 |
| 8 | 0 | 1 | 0.3337029 | 0.0975636 | 0.5698423 |
| 8 | 1 | 1 | 0.3337029 | 0.0975636 | 0.5698423 |
