# Supplementary material for: World Health Organization Danger Signs to predict bacterial sepsis in young infants: A pragmatic cohort study
Source: PLOS Glob Public Health. 2023 Nov 21;3(11):e0001990. doi: 10.1371/journal.pgph.0001990 (PMC10662722; doi:10.1371/journal.pgph.0001990)
Supplement: S1 File — (DOCX) [file pgph.0001990.s009.docx]

STROBE Statement—checklist of items that should be included in reports of observational studies

|  | Item No. | Recommendation | Page  No. | Relevant text from manuscript |
| --- | --- | --- | --- | --- |
| **Title and abstract** | 1 | (*a*) Indicate the study’s design with a commonly used term in the title or the abstract | 1 | Pragmatic cohort study |
|  |  | (*b*) Provide in the abstract an informative and balanced summary of what was done and what was found | 2-3 | “Background… p=0.273)..” |
| Introduction | | | |  |
| Background/rationale | 2 | Explain the scientific background and rationale for the investigation being reported | 4-5 | “ Three million children… …pragmatically reported DS (11).” |
| Objectives | 3 | State specific objectives, including any prespecified hypotheses | 5 | “The objective of this study… suspected sepsis.” |
| Methods | | | |  |
| Study design | 4 | Present key elements of study design early in the paper | 5 | “… was a pragmatic cohort study… “ |
| Setting | 5 | Describe the setting, locations, and relevant dates, including periods of recruitment, exposure, follow-up, and data collection | 5 | “ Kamuzu Central Hospital Lilongwe, Malawi… between June 5^th^ 2018 and April 6^th^ 2020 (including recruitment, exposure, follow-up during hospitalization and data collection)….” |
| Participants | 6 | (*a*) *Cohort study*—Give the eligibility criteria, and the sources and methods of selection of participants. Describe methods of follow-up  *Case-control study*—Give the eligibility criteria, and the sources and methods of case ascertainment and control selection. Give the rationale for the choice of cases and controls  *Cross-sectional study*—Give the eligibility criteria, and the sources and methods of selection of participants | 5-6 | “infants below 3 months of age with suspected sepsis, defined according to the attending staff’s clinically suspicion, were sequentially enrolled…  infants were excluded if they were already on antibiotics >4 hours at the time of enrollment.”  “Infants were followed during hospitalization (follow-up period).” |
|  |  | (*b*) *Cohort study*—For matched studies, give matching criteria and number of exposed and unexposed  *Case-control study*—For matched studies, give matching criteria and the number of controls per case |  |  |
| Variables | 7 | Clearly define all outcomes, exposures, predictors, potential confounders, and effect modifiers. Give diagnostic criteria, if applicable | 8 | See section: Outcome measures… (mortality and culture-positive sepsis, defined as…) |
| Data sources/ measurement | 8* | For each variable of interest, give sources of data and details of methods of assessment (measurement). Describe comparability of assessment methods if there is more than one group | *7-8* | “Clinical data and blood sample collection” in Methods. |
| Bias | 9 | Describe any efforts to address potential sources of bias | 5-6 | “Sequential enrolment around the clock… and consent offered in English or Chichewa (main local language).” |
| Study size | 10 | Explain how the study size was arrived at | 8 | “The sample size was set …in the end only 401 infants were enrolled.” |

Continued on next page

| Quantitative variables | 11 | Explain how quantitative variables were handled in the analyses. If applicable, describe which groupings were chosen and why |  | 8-9 “ All quantitative variables were analysed as such, except for fast breathing and temperature instability which were categorized according to WHO definitions.” |
| --- | --- | --- | --- | --- |
| Statistical methods | 12 | (*a*) Describe all statistical methods, including those used to control for confounding |  | 8 See Statistical  analysis section |
|  |  | (*b*) Describe any methods used to examine subgroups and interactions |  | 8 “potential contaminants  were included in a sensitivity  analysis… |
|  |  | (*c*) Explain how missing data were addressed |  | 9 “ For the predictive  analysis, only complete cases  were used. “ |
|  |  | (*d*) *Cohort study*—If applicable, explain how loss to follow-up was addressed  *Case-control study*—If applicable, explain how matching of cases and controls was addressed  *Cross-sectional study*—If applicable, describe analytical methods taking account of sampling strategy |  | 8-9 “Data were considered missing when infants were transferred within 72 hours after initial admission to the hospital.”  “In separate analyses, odds ratios were calculated using logistic regressions between mean DS (i.e., the number of DS divided by the available DS data) to determine the effect of missing DS data.” |
|  |  | (*e*) Describe any sensitivity analyses |  | 8 “potential contaminants  were included in a sensitivity  analysis…” |
| Results | | | | |
| Participants | 13* | (a) Report numbers of individuals at each stage of study—eg numbers potentially eligible, examined for eligibility, confirmed eligible, included in the study, completing follow-up, and analysed |  | 7 “All infants originally enrolled in the parent study were analyzed…” |
|  |  | (b) Give reasons for non-participation at each stage |  | 8 “About 851 infants presented with suspected sepsis and the vast majority were not enrolled because staff were unable to get consent – either parents declined, or were not approached for consent due to lack of time and/or competing clinical demands.”  (All infants in the parent study were included.) |
|  |  | (c) Consider use of a flow diagram |  | N/A (all were included from parent study). |
| Descriptive data | 14* | (a) Give characteristics of study participants (eg demographic, clinical, social) and information on exposures and potential confounders |  | Table 1 |
|  |  | (b) Indicate number of participants with missing data for each variable of interest |  | Table 1 |
|  |  | (c) *Cohort study*—Summarise follow-up time (eg, average and total amount) |  |  |
| Outcome data | 15* | *Cohort study*—Report numbers of outcome events or summary measures over time |  | 11-15 “Outcome data section: Blood, CSF and mortality … The number of DS was also not significantly associated with bacterial sepsis when including all pathogens and potential contaminants (OR 1.09 [95% CI: 0.93 – 1.28]; p=0.273; Supplemental Figure 3).” |
|  |  | *Case-control study—*Report numbers in each exposure category, or summary measures of exposure |  |  |
|  |  | *Cross-sectional study—*Report numbers of outcome events or summary measures |  |  |
| Main results | 16 | (*a*) Give unadjusted estimates and, if applicable, confounder-adjusted estimates and their precision (eg, 95% confidence interval). Make clear which confounders were adjusted for and why they were included |  | Tables 2, Table 3, page 9 “unadjusted estimates”… page 12 “When adjusting for these co-variables, both the cumulative (OR 1.75 [95% CI: 1.39 – 2.23]; p<0.001) and mean number of DS (1.80 [95% CI: 1.44 – 2.30]; p<0.001) remained highly significantly associated with mortality (Supplemental Table 2).” |
|  |  | (*b*) Report category boundaries when continuous variables were categorized |  | N/A |
|  |  | (*c*) If relevant, consider translating estimates of relative risk into absolute risk for a meaningful time period |  | N/A |

Continued on next page

| Other analyses | 17 | Report other analyses done—eg analyses of subgroups and interactions, and sensitivity analyses |  | 14-15 “Associations when including potential contaminant blood cultures: When considering positive blood cultures for potential contaminants … number of DS was also not significantly associated with bacterial sepsis when including all pathogens and potential contaminants (OR 1.09 [95% CI: 0.93 – 1.28]; p=0.273; Supplemental Figure 3).” |
| --- | --- | --- | --- | --- |
| Discussion | | | | |
| Key results | 18 | Summarise key results with reference to study objectives |  | 15 “This study found… … or potential bacterial contaminants were considered.” |
| Limitations | 19 | Discuss limitations of the study, taking into account sources of potential bias or imprecision. Discuss both direction and magnitude of any potential bias |  | 15 “A number of study limitations… “ |
| Interpretation | 20 | Give a cautious overall interpretation of results considering objectives, limitations, multiplicity of analyses, results from similar studies, and other relevant evidence |  | 15 “The current study findings have implications for a hospital setting… “ |
| Generalisability | 21 | Discuss the generalisability (external validity) of the study results |  | 15-16, 17 “Only few other studies have prospectively estimated… (comparisons with published literature), and more is said in limitation section: caution should be exercised…” |
| Other information | |  | | |
| Funding | 22 | Give the source of funding and the role of the funders for the present study and, if applicable, for the original study on which the present article is based |  | 1 (Funding Support) and (Role of Funder/Sponsor) |
|  |  |  |  |  |

*Give information separately for cases and controls in case-control studies and, if applicable, for exposed and unexposed groups in cohort and cross-sectional studies.

**Note:** An Explanation and Elaboration article discusses each checklist item and gives methodological background and published examples of transparent reporting. The STROBE checklist is best used in conjunction with this article (freely available on the Web sites of PLoS Medicine at http://www.plosmedicine.org/, Annals of Internal Medicine at http://www.annals.org/, and Epidemiology at http://www.epidem.com/). Information on the STROBE Initiative is available at www.strobe-statement.org.
