# Supplementary material for: World Health Organization Danger Signs to predict bacterial sepsis in young infants: A pragmatic cohort study
Source: PLOS Glob Public Health. 2023 Nov 21;3(11):e0001990. doi: 10.1371/journal.pgph.0001990 (PMC10662722; doi:10.1371/journal.pgph.0001990)
Supplement: Attachment — Submitted filename: PGPH-D-23-00809_R1_AN.pdf [file pgph.0001990.s011.pdf]

PLOS Global Public Health

World Health Organization Danger Signs to predict bacterial sepsis in young infants: A pragmatic cohort study

--Manuscript Draft--

**Manuscript Number:**	PGPH-D-23-00809R1
**Article Type:**	Research Article
**Full Title:**	World Health Organization Danger Signs to predict bacterial sepsis in young infants: A pragmatic cohort study
**Short Title:**	Prediction of bacterial sepsis in young infants using WHO Danger Signs.
**Corresponding Author:**	Pascal M Lavoie, MDCM PhD FRCPC<br>The University of British Columbia<br>Vancouver, CANADA
**Order of Authors:**	Omolabake Akinseye, MBBS
	Constantin R. Popescu, MD, MSc
	Msandeni Chiume-Kayuni MMED Peads, MSc
	Michael A. Irvine, PhD
	Norman Lufesi DCM, MPhil
	Tisungane Mvalo, MBBS, MMED
	Niranjan Kissoon, MD
	Matthew O. Wiens, PharmD, PhD
	Pascal M. Lavoie, MDCM PhD FRCPC
**Keywords:**	Neonate; sepsis; Bacteremia; diagnosis; Epidemiology; low- and middle-income countries
**Abstract:**	Background: Bacterial sepsis is generally a major concern in ill infants. To help triaging decisions by front-line health workers in these situations, the World Health Organization (WHO) has developed danger signs (DS). The objective was to evaluate the extent to which nine DS predict bacterial sepsis in young infants presenting with suspected sepsis in a low-income country setting.<br>Methodology: This study pragmatically evaluated nine DS in infants younger than 3 months with suspected sepsis in a regional hospital in Lilongwe, Malawi, between June 2018 and April 2020. Main outcomes were positive blood or cerebrospinal fluid (CSF) cultures for neonatal pathogens and mortality.<br>Findings/Results: Among 401 infants (gestational age [mean ± SD]: 37.1±3.3 weeks, birth weight 2865±785 grams), 41 had positive blood or CSF cultures for a neonatal pathogen. In-hospital mortality occurred in 9.7% (N=39/401) of infants, of which 61.5% (24/39) occurred within 48 hours of admission. All DS were associated with mortality except for temperature instability and tachypnea, whereas none of the DS were significantly associated with bacterial sepsis, except for “unable to feed” (OR 2.25; 95%CI: 1.17 – 4.44; p=0.017). The number of DS predicted mortality (OR: 1.75; 95%CI: 1.43–2.17; p<0.001; AUC: 0.756), but was marginally associated with positive cultures with a neonatal pathogen (OR 1.22; 95%CI: 1.00–1.49; p=0.046; AUC: 0.743). The association between number of DS and mortality remained significant after adjusting for admission weight, the only statistically significant co-variable (OR 1.75 [95% CI: 1.39 - 2.23]; p<0.001). Considering all positive cultures including potential bacterial contaminants resulted a non-significant association between number of DS and sepsis (OR 1.09 [95% CI: 0.93–1.28]; p=0.273).<br>Conclusion/Significance: DS predicted death, but was marginally associated with culture-positive pathogen sepsis in a regional hospital setting. These data imply that the incidence of bacterial sepsis and attributable mortality in infants based on clinical signs may not be accurate, in LMIC settings.
**Additional Information:**	

Powered by Editorial Manager® and ProduXion Manager® from Aries Systems Corporation

Question	Response
**Financial Disclosure**<br><br>Enter a financial disclosure statement that describes the sources of funding for the work included in this submission and the role the funder(s) played. This includes grants and any commercial funding of the work or authors.<br><br>This statement will be typeset if the manuscript is accepted for publication.<br><br>*Please review the submission guidelines and the instructions link below for detailed requirements and guidance.*	The author(s) received no specific funding for this work.
**Competing Interests**<br><br>On behalf of all authors, disclose any competing interests that could be perceived to bias this work.<br><br>This statement will be typeset if the manuscript is accepted for publication.<br><br>*Please review the instructions link below and PLOS Global Public Health's competing interests policy to determine what information must be disclosed at submission.*	The authors have declared that no competing interests exist.
**Data Availability**<br><br>Before publication, Authors are required to make fully available and without restriction all data underlying their findings. Please see our PLOS Data Policy page for detailed information on this policy.<br><br>A **Data Availability Statement**, detailing where the data can be accessed, is	Dataset has been uploaded.

Powered by Editorial Manager® and ProduXion Manager® from Aries Systems Corporation

required at first submission. Insert your Data Availability Statement in the box below.<br><br>Please see the data reporting section of our submission guidelines for instructions on what you need to include in your Data Availability Statement.<br><br>This statement will be typeset if the manuscript is accepted for publication.<br><br>*PLOS allows rare exemptions to address legal and ethical concerns. If you have legal or ethical restrictions, please use the box below to detail these in full sentences for the Journal team to consider.*	

Powered by Editorial Manager® and ProduXion Manager® from Aries Systems Corporation

Manuscript

Click here to access/download;Manuscript;WHO Manuscript - Revised Jul 23 2023.docx

World Health Organization Danger Signs to predict bacterial sepsis in young infants: A pragmatic cohort study

Omolabake Akinseye, MBBS[1,#], Constantin R. Popescu, MD, MSc[2,3,#], Msandeni Chiume-Kayuni MMED Peads, MSc[4,5], Michael A. Irvine, PhD[6,7], Norman Lufesi DCM, MPhil[8], Tisungane Mvalo, MBBS, MMED[9,10], Niranjan Kissoon, MD, MCCM[1,2], Matthew O. Wiens, PharmD, PhD[11], Pascal M. Lavoie, MDCM, PhD[1,2,*]

[#] contributed equally as co-first authors

Affiliations:

[1] Department of Pediatrics, Faculty of Medicine, University of British Columbia, Vancouver, Canada
[2] British Columbia Children's Hospital Research Institute, Vancouver, Canada
[3] Department of Pediatrics, Université Laval, Québec, Canada
[4] Department of Pediatrics, Kamuzu Central Hospital, Lilongwe, Malawi
[5] Kamuzu University of Health Sciences, Lilongwe, Malawi
[6] British Columbia Centre for Disease Control, Vancouver, Canada
[7] Faculty of Health Sciences, Simon Fraser University, Burnaby, Canada
[8] Department of Curative and Medical Rehabilitation, Ministry of Health, Lilongwe, Malawi
[9] University of North Carolina Project Malawi, Lilongwe, Malawi
[10] Department of Pediatrics, University of North Carolina at Chapel Hill School of Medicine, Chapel Hill, NC, USA
[11] Department of Anesthesiology, Pharmacology & Therapeutics, Faculty of Medicine, University of British Columbia, Vancouver, Canada

***Corresponding author:** Dr. Pascal M. Lavoie
BC Children's Hospital Research Institute
4th Floor, Translational Research Building
950 West 28th Avenue
Vancouver, British Columbia V5Z 4H4, Canada
Email: plavoie@bcchr.ca
Fax: +1-604-875-3106

Short title: Prediction of bacterial sepsis in infants.

Conflict of Interest Statement: The authors declare no conflicts of interest.

Funding Support: This study was support by a grant from Grand Challenges Canada (R-ST-POC-1707-04630). PML is supported by the BC Children's Hospital Research Foundation through the Investigator Grant Award Program. CRP was funded by a Sue Carruthers Graduate Studentship from the BC Children's Hospital Foundation. The other authors received no additional funding.

Role of Funder/Sponsor: The funder/sponsor did not participate in the work.

Abbreviations: WHO: World Health Organization; DS: danger signs; LMICs: low and middle-income countries; CSF: cerebrospinal fluid; AUC-ROC: Area Under Curve – Receiver Operator Characteristics; bpm: breaths per minute.

Abstract

Background: Bacterial sepsis is generally a major concern in ill infants. To help triaging decisions by front-line health workers in these situations, the World Health Organization (WHO) has developed danger signs (DS). The objective was to evaluate the extent to which nine DS predict bacterial sepsis in young infants presenting with suspected sepsis in a low-income country setting.

Methodology: This study pragmatically evaluated nine DS in infants younger than 3 months with suspected sepsis in a regional hospital in Lilongwe, Malawi, between June 2018 and April 2020. Main outcomes were positive blood or cerebrospinal fluid (CSF) cultures for neonatal pathogens and mortality.

Findings/Results: Among 401 infants (gestational age [mean ± SD]: 37.1±3.3 weeks, birth weight 2865±785 grams), 41 had positive blood or CSF cultures for a neonatal pathogen. In-hospital mortality occurred in 9.7% (N=39/401) of infants, of which 61.5% (24/39) occurred within 48 hours of admission. All DS were associated with mortality except for temperature instability and tachypnea, whereas none of the DS were significantly associated with bacterial sepsis, except for "unable to feed" (OR 2.25; 95%CI: 1.17 – 4.44; p=0.017). The number of DS predicted mortality (OR: 1.75; 95%CI: 1.43–2.17; p<0.001; AUC: 0.756), but was marginally associated with positive cultures with a neonatal pathogen (OR 1.22; 95%CI: 1.00–1.49; p=0.046; AUC: 0.743). The association between number of DS and mortality remained significant after adjusting for admission weight, the only statistically significant co-variable (OR 1.75 [95% CI: 1.39 - 2.23]; p<0.001). Considering all positive cultures including potential bacterial contaminants resulted a non-significant association between number of DS and sepsis (OR 1.09 [95% CI: 0.93–1.28]; p=0.273).

Conclusion/Significance: DS predicted death, but was marginally associated with culture-positive pathogen sepsis in a regional hospital setting. These data imply that the incidence of bacterial sepsis and attributable mortality in infants based on clinical signs may not be accurate, in LMIC settings.

Introduction

Three million children die each year worldwide in their first month of age, with a significant proportion of these deaths occurring during the first week(1). Severe infections (including sepsis, pneumonia, diarrhea and viral diseases) are common causes of serious illnesses in young infants (2). Up to 25% of cases in low- and middle-income countries (LMICs) have identified bacterial causes (3). However, the lack of diagnostic resources, particularly in LMICs makes it difficult to accurately quantify this burden. This gap has enormous implications in terms of implementing policies and guidelines for the use of antimicrobials to prevent these deaths (1). At the individual level, identifying bacterial causes for sepsis is crucial to prevent fatalities and guide the correct empiric antibiotic therapy in the context of rising antimicrobial resistance. Indeed, the misuse, or even the overuse, of antibiotics carries a risk of subsequent treatment failure due to colonization by resistant bacteria (4, 5).

To assist front-line health workers, the World Health Organization (WHO) and United Nations International Children's Emergency Fund developed algorithms in the early 1990s to help triage interventions for sick children (6). This Integrated Management of Childhood Illness (IMCI) strategy included the identification of clinical signs to alert the need for escalation of care, later termed Danger Signs (DS) (6). Subsequently, the Young Infant Clinical Signs Study Group carried out a multi-centered study in six LMICs and found that seven signs (i.e., history of difficulty feeding, grunting, convulsions, movement only when stimulated, respiratory rate ≥60 breaths per minute (bpm), severe chest indrawing, temperature below 35.5°C or above 37.5°C, prolonged capillary refill, cyanosis and stiff limbs) best predicted the need for higher level of care, with a sensitivity of 74% to 85%, and specificity of 75% to 79%, depending on infants' age (i.e., 0 to 6 days versus 7 to 59 days old) (7). A systematic review of five studies on 17,506 infants found similar results (8).

Over time, the use of DS has been extended to provide a reasonable basis for the initiation of empiric antibiotic treatment (9). However, these signs have neither been developed nor validated to diagnose sepsis of bacterial etiology. Neonatal disorders such as transient tachypnea of the newborn, hypoglycemia, hyaline membrane disease, birth asphyxia, and viral infections (to mention only a few), present like bacterial sepsis (10). Hence, a key question is whether DS can reasonably identify young infants who truly benefit from antibiotic treatment. Only a few studies have attempted to answer this question, but certainly none that have, to the best of our knowledge, been conducted prospectively, in a pragmatic setting where clinicians, not research staff, pragmatically reported DS (11).

The objective of this study was to determine the extent to which WHO DS predict bacterial sepsis in young infants, in a LMIC hospital setting where front-line health workers were asked to assess and record the presence of DS in infants who presented with suspected sepsis.

Materials and Methods

Study approval: The study was approved by the National Health Science Research Committee of Malawi (#17/8/1819) and the University of British Columbia's Children's and Women's Research Ethics Board (#H16-02639). The study was discussed and supported by the chair of the Department of Pediatrics at Kamuzu Central Hospital, leaders from the Pediatric and Child Health Association (PACHA), as well as UNICEF Malawi and the Health Minister of Malawi.

Study design, setting & population: This study was a pragmatic cohort study, and secondary analysis of data from a parent study that aimed to identify molecular RNA-sequencing signatures of bacterial sepsis in young infants with suspected sepsis, defined according to the attending staff's suspicion (12). In the parent study, infants below 3 months of age were sequentially enrolled "around the clock" at the

time they presented to the neonatal or pediatric units at Kamuzu Central Hospital in Lilongwe, Malawi between June 5th, 2018, and April 6th, 2020 (including recruitment, exposure, follow-up and data collection). To minimize bias towards false-negative blood culture, infants were excluded if they were already on antibiotics for >4 hours at the time of enrollment. About 851 infants presented with suspected sepsis and the vast majority were not enrolled because staff were unable to get consent – either parents declined, or were not approached for consent due to lack of time and/or competing clinical demands. Written informed consent was obtained from parents/guardian of each participant, in English or Chichewa (main local language). Enrolled infants were followed during their entire hospitalization at Kamuzu Central Hospital, until transferred to another facility or discharged home.

The Kamuzu Central Hospital the largest referral hospital in central Malawi, where approximatively 3,000 infants are admitted to its neonatal unit annually. “Suspected sepsis” was defined as per the clinical staff, essentially in an infant that looked unwell where bacterial sepsis could not be excluded clinically. Before the study, the staff at Kamuzu Central Hospital did not have routinely access to blood culture or other ancillary tests, IV fluids and gavage feeds were also most often not available due to limited resources and supplies. Available interventions include antibiotics, assistance with feeding and routine surgeries when necessary. Oxygen and continuous positive pressure ventilation were only available to an extremely limited number of infants, on a limited basis when the hospital had electrical power. Invasive mechanical ventilation was not possible. During the study, investigators did not have access to information that could identify participants, with the exception of the consent forms kept under the responsibility of the local study PI (MCK). The study was administered and local research staff were hired through a partnership with the Parent and Child Health Initiative (PACHI), a Malawian non-profit non-governmental organization, which works in collaboration with the Ministry of Health.

Clinical data and blood samples collection: Each infant had an initial clinical evaluation by a healthcare worker (or staff, either a nurse or clinical officer). The presence of the following nine DS were prospectively collected at presentation, using electronic forms, on a tablet device: i) history of not feeding well (interviewing the parent/caregiver), entered as “feeding well”, “unable to feed” or “uninterested in feeding” (combining the latter two categories), ii) fast breathing ≥60 bpm (with respiratory rate visually counted by attended clinician), iii) temperature instability, defined as axillary body temperature <35.5°C or >38°C, (measured using an electronic Welch Allyn SureTemp thermometer, model 692), iv) drowsiness/difficulty to wake, v) grunting, vi) severe chest recession, vii) central cyanosis, viii) movements only when stimulated or no movement at all and ix) convulsions (defined pragmatically by attending clinician). All infants also had a standardized aerobic blood culture (minimum 2 mL of blood) collected within 4 hours of presentation, using a rigorously implemented aseptic protocol (with in-training of all front-line healthcare providers prior to study initiation), as detailed (13). Infants also had cerebrospinal fluid (CSF) and urine cultures collected at the discretion of the clinical staff, availability of resources and cost of the test to be paid by parents.

Blood cultures were processed into BACTEC PEDS Plus culture bottles incubated for 5 days in a BD BACTEC 9050 instrument. In keeping with a pragmatic design, the study did not attempt to enforce strict criteria to define suspected sepsis, DS or illness status. Identification of organisms from blood or CSF cultures was completed in-house using bioMerieux API, and BD Crystal kits at a local University of North Carolina-affiliated research facility, as described (12). Bacterial isolates that could be restored (~60% of isolates) were also analyzed for further confirmation of identity on a Microflex LT MALDI-TOF mass spectrometer, using the BioTyper software version 3.1 (Bruker Daltonik, Germany). Culture results were made available to clinicians involved in the care of the infants.

Outcome measures: The primary outcomes of interest were *mortality*, and *culture-positive sepsis* defined as the bacterial growth of a known neonatal pathogen in blood or CSF culture sample. Micro-organisms not commonly considered as pathogens in term or near-term infants, such as coagulase-negative Staphylococcus, Micrococcus or Bacillus were excluded (14), but these potential contaminants were included in a sensitivity analysis using an alternate bacterial sepsis outcome "including potential contaminants" (**Supplemental Table 1**). In-hospital mortality was recorded until discharge home or transfer to another hospital facility. Data were considered missing when infants were transferred within 72 hours after initial admission to the hospital.

Statistical Analysis: In the parent study, the sample size was set conveniently at 500 infants over two years, but the study needed to be stopped early, in April 2020 due to the COVID-19 pandemic, so in the end only 401 infants were enrolled. All infants originally enrolled in the parent study were analyzed. Demographic characteristics were reported descriptively using the mean/median with dispersion. The nine aforementioned WHO DS were used as predictors. All quantitative variables were analyzed as such, except for fast breathing and temperature instability which were categorized according to WHO definitions (see above). Odds ratios were calculated for each DS and the main outcomes of bacterial sepsis or mortality. Logistic regressions were conducted between the number of DS and the main outcomes. In separate analyses, odds ratios were calculated using logistic regressions between mean DS (i.e., the number of DS divided by the available DS data) to determine the effect of missing DS data. Unadjusted logistic regression models, including demographic variables, were conducted to determine the predictive value of the DS. The predictive performance of DS for each outcome was assessed using the area under receiver operator characteristic curve (AUC-ROC). Sociodemographic variables associated with mortality, as well as their independent effects were analyzed using regression models. Only complete cases were used in the analysis for associations with individual DS, or the

outcome "cumulative number of DS". However, analyses were also conducted using "mean number of DS", to account for missing values, with no significant differences in results. Statistical significance was considered at p-value <0.05 with no adjustment made for multiple testing in unadjusted models. Analyses were performed using R (version 4.1.0) and SPSS version 20 (IBM, Armonk, NY, USA).

Inclusivity in global research: Additional information regarding the ethical, cultural, and scientific considerations specific to inclusivity in global research is included in the **Supporting Information**.

Results

Study population: The majority of the 401 infants included in the study were term or late preterm infants, born vaginally, presented from home in their first week of post-natal life, and had roughly equal female/male distribution (**Table 1**). Most infants were reported by clinicians to look ill at the time of initial presentation (69.3%; 278/401), 22.2% (89/401) were severely ill, and 8.5% appeared well (34/401). In total, 79.8% (320/401) presented at least one DS. Overall, 56.9% (228/401) of infants had been ill for at least 24 hours prior to initial presentation, and their parents had traveled a median distance of 17 kilometers, often by foot or taxi, to reach the nearest hospital. In total, 94/401 (23.4%) infants had received antibiotics prior to the blood culture, for a median of 2 hours (range 0.75 to 3.5 hours). The duration of antibiotic use in infants ranged from 1 to 30 days (**Table 1**). Few infants with negative blood culture had antibiotics discontinued after 48 hours (21/401, 5.2%).

Table 1: Socio-demographic characteristics of the study population.

**Participant characteristics**		**N with data**
Gestational age at birth (weeks), mean (SD)	37.1 (3.3)	397
Birth weight (gram), mean (SD)	2865 (785)	380

Female, no (%)	185 (46)	401
Admitted from, no (%)		401
Inborn (i.e., admitted from birth hospital)	161 (40)	
Transferred from another facility	224 (56)	
Home	16 (4)	
Post-natal age at presentation (days), median (IQR)	4 (2-16)	401
Mode of delivery, no (%)		398
Caesarean section	101 (25.2)	
Vaginal delivery	297 (71.8)	
Duration of illness before presentation to hospital (hours), median (IQR)	24 (12-48)	341
Distance of travel to hospital (km), median (IQR)	17 (11-25)	342
Illness status, number on infants (%)		401
Well	34 (8.5)	
[illegible]	278 (69.3)	
Severely ill	89 (22.2)	
Duration of antibiotics (days), median (IQR)	6 (5-9)	401
Duration of hospital stay (days), median (IQR)	7 (5-9)	401
Danger Signs, number of infants (%)		
Not feeding well	171 (43.3)	395
Convulsions	28 (7.0)	401
Drowsy / Difficult to wake	33 (8.2)	401
Movements only when stimulated or no movements	26 (6.5)	401

Fast breathing >=60 per min	104 (27.1)	384
Grunting	30 (7.5)	401
Severe chest recessions	51 (12.7)	401
Temperature instability >38°C or <35.5°C	118 (32.7)	361
Central cyanosis	29 (7.2)	401

Outcome data: The proportion of infants with bacterial sepsis, considering only known pathogens in term and near-term infants, was 10.2% (41/401). Blood (39/401) and CSF (2/204) cultures were positive for pathogens in 9.7% and 1.0% of the infants, respectively. One infant had a contaminating blood culture, but grew unidentified gram-negative diplococci in the CSF, so was classified as bacterial sepsis with a pathogen. Of 230 infants under 7 days old, 21 (9.1%) had bacterial sepsis with a pathogen versus 20 of the 171 infants (11.7%; p=0.402 by chi-square test) ≥ 7 days old. The proportion of gram-positive sepsis (21/39) and gram-negative sepsis (20/39) were similar among all infants (**Supplemental Table 1**), and were also similar between infants under 7 days (9 versus 12) and infants ≥ 7 days old (12 versus 8; p=0.272 by chi-square test, noting however the small sample size for comparison). The proportion of sepsis was also similar in inborn infants (19/161) versus infants transferred from another hospital facility (22/224) or admitted from home (0/16). Three infants were transferred before 72 hours from admission, so the mortality outcome was considered missing in these infants. Among the remaining infants, mortality was 9.7% (i.e., 39/398), of which 61.5% [24/39] died within 48 hours.

Associations with mortality and culture-positive outcomes: The following DS were significantly associated with mortality (**Table 2**): *not feeding well*, *convulsions*, *drowsy/difficult to wake*, *movements only when stimulated or no movements*, *grunting*, *severe chest recessions*, *central cyanosis*. In contrast,

fast breathing or *temperature instability* were not significantly associated with mortality. The number of DS was also significantly associated with mortality (OR 1.75 [95%CI: 1.43 – 2.17] per cumulative DS; p<0.001; AUC: 0.743) (**Supplemental Figure 1**). Admission weight was significantly associated with mortality, but neither age of the mother, gestational age, birth weight nor sex were significantly associated with mortality (**Supplemental Table 2**). When adjusting for these co-variables, both the cumulative (OR 1.75 [95% CI: 1.39 - 2.23]; p<0.001) and mean number of DS (1.80 [95% CI: 1.44 – 2.30]; p<0.001) remained highly significantly associated with mortality (**Supplemental Table 2**).

Table 2: Associations between DS and mortality.

**Variable**	**Odds ratio (OR)**	**95%CI**	**P value**
Not feeding well	7.21	3.27 - 18.2	<0.001
Convulsions	3.52	1.31 - 8.59	0.008
Drowsy / Difficult to wake	5.04	2.12 - 11.4	<0.001
Movements only when stimulated or no movements	4.89	1.88 - 11.9	<0.001
Fast breathing >=60 per min	1.86	0.87 - 3.85	0.100
Grunting	3.95	1.54 - 9.34	0.002
Severe chest recessions	3.01	1.34 - 6.37	0.005
Temperature instability >38°C or <35.5°C	1.70	0.75 - 3.75	0.191
Central cyanosis	4.15	1.62 - 9.87	0.002

Bacterial sepsis with a known neonatal pathogen was significantly associated with mortality (OR 3.07 [95%CI: 1.28 – 6.82]; p = 0.008). However, the only DS significantly associated with bacterial sepsis when considering only known neonatal pathogens was *not feeding we*[illegible]R 2.25 [95%CI: 1.17 – 4.44]; p=0.017) (**Table 3**). Associations between convulsions, drowsiness, lethargy (i.e., movements only

when stimulated or no movement), tachypnea, grunting, severe chest recession, temperature instability and central cyanosis were all non-significant (**Table 3**). Association was between cumulative number of DS and pathogen bacterial sepsis was marginally significant (OR 1.22 [95% CI: 1.00 – 1.49]; p=0.046) (**Supplemental Figure 2**). At discharge, clinical staff selected non-bacterial sepsis in 76.3% (306/401), bacterial sepsis in 15.5% (62/401) and “other non-sepsis syndromes” in 8.2% (33/401) of cases, from these options, for study purposes.

Table 3: Association between DS and bacterial sepsis when organism is a known pathogen.

**Variable**	**Odds ratio (OR)**	**95%CI**	**P value**
Not feeding well	2.25	1.17 - 4.44	0.017
Convulsions	0.65	0.10 - 2.30	0.571
Drowsy / Difficult to wake	0.86	0.20 - 2.57	0.811
Movements only when stimulated or no movements	1.65	0.46 - 4.59	0.382
Fast breathing >=60 per min	1.06	0.49 - 2.17	0.868
Grunting	2.38	0.84 – 5.89	0.077
Severe chest recessions	2.17	0.92 – 4.74	0.061
Temperature instability >38°C or <35.5°C	1.60	0.78 - 3.24	0.193
Central cyanosis	1.01	0.32 - 3.03	0.994

<u>Associations when including potential contaminant blood cultures:</u> When considering potential blood culture contaminants (e.g., *micrococcus*, *Corynebacterium*, *bacillus* and *coagulase-negative staphylococcus* species and non-pathogenic *streptococcus* species such as *S. oralis*), [illegible] infants had positive blood or CSF cultures. Bacterial culture positivity was not significantly associated with mortality (OR 1.74 [95% CI: 0.82 – 3.53]; p=0.135), or any DS when including contaminants

(**Supplemental Table 3**). The number of DS was also not significantly associated with bacterial sepsis when including contaminants (OR 1.09 [95% CI: 0.93 – 1.28]; p=0.273; **Supplemental Figure 3**).

Discussion

This study found that most WHO DS were significantly associated with mortality, but only one DS (i.e., not feeding well) as significantly associated with culture-proven bacterial sepsis. Additionally, the majority of DS were not associated with culture-proven bacterial sepsis in this LMIC neonatal cohort. The results held true whether only bacterial pathogens, or potential bacterial contaminants were considered. To the best of our knowledge, the current study is the first to pragmatically examine whether DS predict bacterial sepsis in infants. These findings were obtained in an LMIC setting where DS were prospectively identified by front line healthcare workers as they would normally do pragmatically outside a research protocol – we assumed that this study design would more closely reflects the real-life utility of DS. Few studies have attempted to determine how DS can predict bacterial sepsis caused by bacterial pathogens in young infants. A retrospective Brazilian study reviewed data on 83 sick neonates over a 12-month period, and came to the same conclusion that DS alone or in combination did not predict severe serious bacterial infection (15). The findings of the current study are also consistent with those of another study carried out in a primary care, rural setting in Bangladesh, India and Pakistan. In the latter study, clinical signs could not differentiate infants with viral or bacterial illnesses (16). The current study findings have implications for a hospital setting, but further research is needed to determine the generalizability of the results to other hospital contexts.

In addition to the main findings, the current study also found that only a minor proportion of infants with suspected sepsis showed positive blood culture. Only few other studies have prospectively estimated the incidence of bacterial sepsis in LMIC cohorts, and it is therefore useful to present the

current findings in the context of those other studies. A hospital centre study in Delhi, India, included 13,530 neonates (all comers; mean GA±SD: 36.0±3.4 weeks) monitored daily for clinical signs of sepsis with per-protocol completion of a sepsis work-up (blood culture and lumbar puncture) prior to initiating antibiotics, and found a 14.3% incidence of culture-positive sepsis (17), which is similar to the findings of the current study. Another study in community settings in Madagascar, Senegal and Cambodia reported 8.2% culture-confirmed sepsis among 514 infants assessed for “possible serious bacterial infection” out of 3,688 neonates (18). One large study followed 12,622 live births up to 60 days of age in rural India between April 2002 and March 2005, with daily home visits and clinical assessment, and standardized blood and CSF cultures following enrollment of infants with suspected sepsis. It reported a 6.3% incidence of culture-confirmed sepsis (53 with positive blood cultures out of 842 infants assessed, excluding potential contaminants), for a population-level incidence of culture-confirmed sepsis of 6.7 per 1,000 births (19). Taken together, these findings provide robust data on the incidence of bacterial sepsis in LMIC settings, to inform interventions and form the basis for future studies to measure the impact of those interventions.

Blood cultures are the gold standard for diagnosing bacterial sepsis in young infants (20). However, these tests lack sensitivity in neonates (21). In the current study we were careful to prescribe a minimum blood volume to maximize culture yield. Yet, a key question is whether we misestimate the proportion of bacterial sepsis in infants in whom cultures are negative? The Aetiology of Neonatal Infection in South Asia (ANISA) study (22) also attempted to identify the cause of sepsis for 6022 cases of possible serious bacterial infections in infants in the first 60 days after birth, in a population of 63,114 livebirth). Bacterial and viral (e.g., respiratory syncytial virus) causes were identified in 16% and 12% of cases, respectively. For the remaining 72% cases, no pathogen could be identified despite systematic blood and respiratory cultures, which is consistent with findings from the current study.

Evidently the use of antibiotics in the current study was high, with the vast majority of infants receiving antibiotics for a minimum of five days during hospitalization, as per hospital practices. As in other LMIC settings, blood cultures were not routinely available at the study site prior to the study. As such, no approach had been developed to stop antibiotics after 48 hours given a negative blood culture. In another study in Thailand, the duration of antibiotic use was also high with durations of 15 and 8 days for culture-proven and culture-negative early-onset neonatal sepsis, respectively (23). These results highlight the limits of having to rely on DS, and potential coercive effect of the lack of laboratory resources capable of providing basic laboratory test results to help "rule out" sepsis (24).

A strength of this study is its robust outcomes, with standardized blood culture procedures to ensure that the chances of contamination during blood sampling were minimized, and robust microbiological identification methods including clinical laboratory protocols on-site augmented by advanced MALDI-TOF species determination in another, North American clinical microbiology laboratory. The strong association between bacterial sepsis and mortality provides reassurance infants with positive cultures were correctly identified. However, a number of study limitations should be acknowledged. Although a sizeable proportion of the recruited infants presented from home, data were collected in a single hospital-type setting; as such, caution should be exercised when generalizing to other hospital settings or extrapolating to non-hospital primary care settings. For logistical reasons CSF was collected from only approximately half of the infants, which may have underestimated the incidence of bacterial sepsis. Similarly, anaerobic blood cultures were not collected, which may have resulted in missed bacteremia. Some infants received antibiotics prior to blood culture, which may have lowered bacterial sepsis estimates. Moreover, due to the extremely limited diagnostic capabilities of the study LMIC

settings, in most cases, causes of deaths or alternate diagnoses could not be determined. Similarly, the study did not include non-bacterial testing, which precluded identification of viral sepsis syndromes.

In conclusion, our study validates the use of WHO DS to predict fatal outcomes, but not bacterial sepsis, in young infants. This study further emphasizes the need for better diagnostics for bacterial sepsis, to ensure that health gains continue to improve, in areas of the world where sepsis represents a huge burden and where antimicrobial resistance is also increasing at alarming rates (25).

Acknowledgments

We thank Bentry Tembo, Rhoda Chifisi and Blessings Chiluzi for help with recruitment and data collection, Charles Mwansambo, Patience Johane and the staff at the PACHI for administrative and operational support during the study, Robert Krysiak, Gerald Tegha and the staff of the University of North Carolina – Malawi lab for performing all bacterial cultures and identification, on-site, in Lilongwe, according to clinical laboratory standards, Dr. David Goldfarb for expert input into data interpretation, Amber Johnson from the British Columbia Children's Hospital Microbiology Laboratory for secondary confirming bacterial isolates by mass spectrometry.

REFERENCES

1. GBD 2016 Causes of Death Collaborators. Global, regional, and national age-sex specific mortality for 264 causes of death, 1980-2016: a systematic analysis for the Global Burden of Disease Study 2016. Lancet. 2017;390(10100):1151-210.
2. Rudd KE, Johnson SC, Agesa KM, Shackelford KA, Tsoi D, Kievlan DR, et al. Global, regional, and national sepsis incidence and mortality, 1990-2017: analysis for the Global Burden of Disease Study. Lancet. 2020;395(10219):200-11.
3. Fleischmann-Struzek C, Goldfarb DM, Schlattmann P, Schlapbach LJ, Reinhart K, Kissoon N. The global burden of paediatric and neonatal sepsis: a systematic review. The Lancet Respiratory medicine. 2018;6(3):223-30.
4. Costelloe C, Metcalfe C, Lovering A, Mant D, Hay AD. Effect of antibiotic prescribing in primary care on antimicrobial resistance in individual patients: systematic review and meta-analysis. BMJ. 2010;340:c2096.
5. Klingenberg C, Kornelisse RF, Buonocore G, Maier RF, Stocker M. Culture-Negative Early-Onset Neonatal Sepsis - At the Crossroad Between Efficient Sepsis Care and Antimicrobial Stewardship. Front Pediatr. 2018;6:285.
6. Costello A. Integrated management of childhood illness. Lancet. 1997;350(9087):1266.
7. Young Infants Clinical Signs Study Group. Clinical signs that predict severe illness in children under age 2 months: a multicentre study. The Lancet. 2008;371(9607):135-42.
8. Opiyo N, English M. What clinical signs best identify severe illness in young infants aged 0-59 days in developing countries? A systematic review. Archives of disease in childhood. 2011;96(11):1052-9.

9. World Health Organization. Pocket Book of Hospital Care for Children: Guidelines for the Management of Common Childhood Illnesses. WHO Guidelines Approved by the Guidelines Review Committee. 2nd ed. Geneva, 2013.
10. Wynn JL, Polin RA. Progress in the management of neonatal sepsis: the importance of a consensus definition. Pediatr Res. 2018;83(1-1):13-5.
11. Clinical prediction of serious bacterial infections in young infants in developing countries. The WHO Young Infants Study Group. Pediatr Infect Dis J. 1999;18(10 Suppl):S23-31.
12. Popescu CR, Tembo B, Chifisi R, Cavanagh MMM, Lee AH, Chiluzi B, et al. Whole blood genome-wide transcriptome profiling and metagenomics next-generation sequencing in young infants with suspected sepsis in a low-and middle-income country: A study protocol. Gates Open Res. 2020;4:139.
13. Popescu C, Tembo B, Chifisi R, Cavanagh M, Chiluzi B, Lufesi N, et al. Procedure policies, data collection form and model consent form (supplemental material)~Improving the Early Diagnosis of Neonatal Sepsis in Malawi. In: Scholars Portal Dataverse, editor. 2 ed 2020.
14. Downie L, Armiento R, Subhi R, Kelly J, Clifford V, Duke T. Community-acquired neonatal and infant sepsis in developing countries: efficacy of WHO's currently recommended antibiotics--systematic review and meta-analysis. Archives of disease in childhood. 2013;98(2):146-54.
15. Ronfani L, Vilarim JN, Dragovich D, Bacalhau AF, Cattaneo A. Signs of severe bacterial infection in neonates. J Trop Pediatr. 1999;45(1):48-51.
16. Arvay ML, Shang N, Qazi SA, Darmstadt GL, Islam MS, Roth DE, et al. Infectious aetiologies of neonatal illness in south Asia classified using WHO definitions: a primary analysis of the ANISA study. The Lancet Global health. 2022;10(9):e1289-e97.
17. Study IotDNI. Characterisation and antimicrobial resistance of sepsis pathogens in neonates born in tertiary care centres in Delhi, India: a cohort study. The Lancet Global health. 2016;4(10):e752-60.

18. Huynh BT, Kermorvant-Duchemin E, Chheang R, Randrianirina F, Seck A, Ratsima EH, et al. Severe bacterial neonatal infections in Madagascar, Senegal, and Cambodia: A multicentric community-based cohort study. Plos Medicine. 2021;18(9).
19. Panigrahi P, Chandel DS, Hansen NI, Sharma N, Kandefer S, Parida S, et al. Neonatal sepsis in rural India: timing, microbiology and antibiotic resistance in a population-based prospective study in the community setting. J Perinatol. 2017;37(8):911-21.
20. Polin RA. Management of neonates with suspected or proven early-onset bacterial sepsis. Pediatrics. 2012;129(5):1006-15.
21. Popescu CR, Cavanagh MMM, Tembo B, Chiume M, Lufesi N, Goldfarb DM, et al. Neonatal sepsis in low-income countries: epidemiology, diagnosis and prevention. Expert Rev Anti Infect Ther. 2020;18(5):443-52.
22. Saha SK, Schrag SJ, El Arifeen S, Mullany LC, Shahidul Islam M, Shang N, et al. Causes and incidence of community-acquired serious infections among young children in south Asia (ANISA): an observational cohort study. Lancet. 2018;392(10142):145-59.
23. Kiatchoosakun P, Jirapradittha J, Sirikarn P, Laopaiboon M, Pattanittum P, Chandrakachorn W, et al. Early-Onset Neonatal Sepsis and Antibiotic Use in Northeast Thailand. Am J Perinatol. 2019;36(12):1295-303.
24. Lavoie PM, Popescu CR, Molyneux EM, Wynn JL, Chiume M, Keitel K, et al. Rethinking management of neonates at risk of sepsis. Lancet. 2019;394(10195):279-81.
25. Iregbu K, Dramowski A, Milton R, Nsutebu E, Howie SRC, Chakraborty M, et al. Global health systems' data science approach for precision diagnosis of sepsis in early life. Lancet Infect Dis. 2022;22(5):e143-e52.

Click here to access/download
Supporting Information
REB Approval - PGPH-D-23-00809.pdf

Click here to access/download
Supporting Information
WHO Manuscript - [Supplemental Data] - Jul 11 2023.docx

Click here to access/download
Supporting Information
STROBE-checklist WHO DS Manuscript.docx

Click here to access/download
Supporting Information
Inclusivity-in-global-research-questionnaire.docx

Click here to access/download
Supporting Information
WHO DS - Final DataSet - Jul 11 2023.xlsx

World Health Organization Danger Signs to predict bacterial sepsis in young ~~newborns~~ infants: A pragmatic ~~prospective~~ cohort study

Omolabake Akinseye, MBBS[1,#], Constantin R. Popescu, MD, MSc[2,3,#], Msandeni Chiume-Kayuni MMED Peads, MSc[4,5], Michael A. Irvine, PhD[6,7], Norman Lufesi DCM, MPhil[8], Tisungane Mvalo, MBBS, MMED[9,10], Niranjan Kissoon, MD, MCCM[1,2], Matthew O. Wiens, PharmD, PhD[11], Pascal M. Lavoie, MDCM, PhD[1,2,*]

[#] contributed equally as co-first authors

Affiliations:
[1] Department of Pediatrics, Faculty of Medicine, University of British Columbia, Vancouver, Canada
[2] British Columbia Children's Hospital Research Institute, Vancouver, Canada
[3] Department of Pediatrics, Université Laval, Québec, Canada
[4] Department of Pediatrics, Kamuzu Central Hospital, Lilongwe, Malawi
[5] Kamuzu University of Health Sciences, Lilongwe, Malawi
[6] British Columbia Centre for Disease Control, Vancouver, Canada
[7] Faculty of Health Sciences, Simon Fraser University, Burnaby, Canada
[8] Department of Curative and Medical Rehabilitation, Ministry of Health, Lilongwe, Malawi
[9] University of North Carolina Project Malawi, Lilongwe, Malawi
[10] Department of Pediatrics, University of North Carolina at Chapel Hill School of Medicine, Chapel Hill, NC, USA
[11] Department of Anesthesiology, Pharmacology & Therapeutics, Faculty of Medicine, University of British Columbia, Vancouver, Canada

***Corresponding author:** Dr. Pascal M. Lavoie
BC Children's Hospital Research Institute
4th Floor, Translational Research Building
950 West 28th Avenue
Vancouver, British Columbia V5Z 4H4, Canada
Email: plavoie@bcchr.ca
Fax: +1-604-875-3106

Short title: Prediction of bacterial sepsis in infants.

Conflict of Interest Statement: The authors declare no conflicts of interest.

Funding Support: This study was support by a grant from Grand Challenges Canada (R-ST-POC-1707-04630). PML is supported by the BC Children's Hospital Research Foundation through the Investigator Grant Award Program. CRP was funded by a Sue Carruthers Graduate Studentship from the BC Children's Hospital Foundation. The other authors received no additional funding.

Role of Funder/Sponsor: The funder/sponsor did not participate in the work.

Abbreviations: WHO: World Health Organization; DS: danger signs; LMICs: low and middle-income countries; CSF: cerebrospinal fluid; AUC-ROC: Area Under Curve – Receiver Operator Characteristics; bpm: breaths per minute.

Abstract

Background: Bacterial sepsis is generally a major concern in ill infants~~ presenting with acute illnesses~~. To help triaging decisions by front-line health workers in these situations, the World Health Organization (WHO) has developed danger signs (DS). The objective was to evaluate the extent to which nine DS predict bacterial sepsis in young infants presenting with suspected sepsis in a low-income country setting.

~~The World Health Organization (WHO) has developed danger signs (DS) to help front-line health workers triage interventions in young infants children with severe acute illnesses. Our The objective was to evaluate the extent to which nine DS predict bacterial sepsis in young infants presenting with acute illnessthese children.~~

Methodology~~/Principal Findings~~: This ~~prospective~~ study pragmatically evaluated nine DS in infants younger than 3 months with suspected sepsis in a ~~large~~ regional hospital in Lilongwe, Malawi, between June 2018 and April 2020. M~~The m~~ain outcomes were positive blood or cerebrospinal fluid (CSF) cultures for neonatal pathogens and mortality.

Findings/Results: Among 401 infants (gestational age [mean ± SD]: 37.1±3.3 weeks, birth weight 2865±785 grams), 41 had positive blood ~~Blood (n=85/401) and~~or CSF ~~(n=2/204)~~ cultures for a neonatal pathogen~~were positive in 21.2% and 1% of infants, respectively (N=401; gestational age mean ± SD: 37.1±3.3 weeks, birth weight 2865±785 grams)~~. In-hospital ~~deaths~~ mortality occurred in 9.7% (N=39/401) of infants, of which ~~(~~61.5% (24/39) occurred within 48 hours ~~-~~of admission~~)~~. ~~In univariate analyses, a~~All DS were associated with mortality except for temperature instability and tachypnea, whereas ~~“infant was unable to feed” was the only~~ none of the DS were significantly associated with

bacterial sepsis, except for “unable to feed” (OR 2.25; 95%CI: 1.17 – 4.44; p=0.017). ~~After co-variable adjustments,~~The number of DS predicted mortality (OR: 1.75; 95%CI: 1.43–2.~~16~~17; p<0.001; AUC~~-ROC~~: 0.756), but was ~~not~~ marginally ~~predicted~~associated with ~~positive cultures~~ positive cultures with a neonatal pathogen (OR ~~1.08~~1.22; 95%CI: ~~0.92~~1.00–1.49~~30~~; p=0.046; AUC: 0.743~~336~~). The association between number of DS and mortality remained significant after adjusting for admission weight, the only statistically significant co-variable (OR 1.75 [95% CI: 1.39 - 2.23]; p<0.001). ~~Whether~~ Considering all positive cultures ~~including~~including potential bacterial ~~bacterial~~ contaminants ~~were included or not did not change~~resulted a non-significant ~~results~~association between number of DS and sepsis (OR 1.09 [95% CI: 0.93~~—~~1.28]; p=0.273). ~~meaningfully.~~

Conclusion/Significance: DS predicted ~~fatal outcomes~~death, but ~~not marginally~~was marginally associated with ~~predicted culture-positive~~ culture-~~pathogen~~positive pathogen sepsis ~~– cultures sepsis~~ in a ~~large~~ regional hospital setting. These data imply that the incidence of bacterial sepsis and attributable mortality in infants based on clinical signs ~~are unlikely to~~may not be accurate~~ly predicted based on clinical signs alone~~, ~~in infants~~ in LMIC settings.

Introduction

Three million children die each year worldwide in their first month of age, with a significant proportion of these deaths occurring during the first week(1). Severe infections (including sepsis, pneumonia, diarrhea and viral diseases) are common causes of serious illnesses in young infants (2). Up to 25% of cases in low- and middle-income countries (LMICs) have identified bacterial causes (3). However, the lack of diagnostic resources, particularly in LMICs makes it difficult to accurately quantify this burden. This gap has enormous implications in terms of implementing policies and guidelines for the use of antimicrobials to prevent these deaths (1). At the individual level, identifying bacterial causes for sepsis is crucial to prevent fatalities and guide the correct empiric antibiotic therapy in the context of rising antimicrobial resistance. Indeed, the misuse, or even the overuse, of antibiotics carries a risk of subsequent treatment failure due to colonization by resistant bacteria (4, 5).

To assist front-line health workers, the World Health Organization (WHO) and United Nations International Children’s Emergency Fund developed algorithms in the early 1990s to help triage interventions for sick children (6). This Integrated Management of Childhood Illness (IMCI) strategy included the identification of clinical signs to alert the need for escalation of care, later termed Danger Signs (DS) (6). Subsequently, the Young Infant Clinical Signs Study Group carried out a multi-centered study in six LMICs and found that seven signs (i.e., history of difficulty feeding, grunting, convulsions, movement only when stimulated, respiratory rate ≥60 breaths per minute (bpm), severe chest indrawing, temperature below 35.5°C or above 37.5°C, prolonged capillary refill, cyanosis and stiff limbs) best predicted the need for higher level of care, with a sensitivity of 74% to 85%, and specificity of 75% to 79%, depending on infants’ age (i.e., 0 to 6 days versus 7 to 59 days old) (7). A systematic review of five studies on 17,506 infants found similar results (8).

Over time, the use of DS has been extended to provide a reasonable basis for the initiation of empiric antibiotic treatment (9). However, these signs have neither been developed nor validated to diagnose sepsis of bacterial etiology. Neonatal disorders such as transient tachypnea of the newborn, hypoglycemia, hyaline membrane disease, birth asphyxia, and viral infections (to mention only a few), present like bacterial sepsis (10). Hence, a key question is whether DS can reasonably identify ~~newborns or~~ young infants who truly benefit from antibiotic treatment. Only a few studies have attempted to answer this question, but certainly none that have, to the best of our knowledge, been conducted prospectively, in a pragmatic setting where clinicians, not research staff, pragmatically reported DS (11).

The objective of this study was to determine the extent to which WHO DS predict bacterial sepsis in young infants, in a LMIC hospital setting where front-line health workers were asked to assess and record the presence of DS in infants who presented with suspected sepsis.

Materials and Methods

Study approval: The study was approved by the ~~University of British Columbia's Children's and Women's Research Ethics Board (#H16-02639) and the~~ National Health Science Research Committee of Malawi (#17/8/1819) and the University of British Columbia's Children's and Women's Research Ethics Board (#H16-02639). ~~In addition, the study was discussed~~The study was discussed and supported by ~~with~~ the chair of the Department of Pediatrics at Kamuzu Central Hospital, leaders from the Pediatric and Child Health Association (PACHA), as well as UNICEF Malawi and the Health Minister of Malawi.

Study design, setting & population: This study was a pragmatic cohort study, and secondary analysis of data from a parent study that aimed to identify molecular RNA-sequencing signatures of bacterial sepsis in young infants with suspected sepsis, defined according to the attending staff's suspicion (12). In the parent study, infants below 3 months of age were sequentially enrolled "around the clock" at the time they presented ~~with suspected sepsis before,~~ to the neonatal or pediatric units at Kamuzu Central Hospital in Lilongwe, Malawi between June 5th, 2018, and April 6th, 2020 (including recruitment, exposure, follow-up and data collection). To minimize bias towards false-negative blood culture, infants were excluded if they ~~had~~ were already on ~~received a~~antibiotics for >4 hours at the time of enrollment. ~~In the original (parent) study, a~~About 851 infants presented with suspected sepsis and the vast majority were not enrolled because staff were unable to get consent ~~-~~– either parents declined, or ~~the conditions were not sufficiently permissive~~were not approached for ~~proper~~ consent due to lack of time and/or ~~other~~ competing clinical demands. Written informed consent was obtained from ~~all~~ parents/guardian of each participant, in English or Chichewa (main local language). Enrolled infants were followed during their entire hospitalization at Kamuzu Central Hospital, until transferred to another facility or discharged home.

The Kamuzu Central Hospital the largest referral hospital in central Malawi, where approximativ~~v~~ely 3,000 infants are admitted to its neonatal unit annually. "Suspected sepsis" was defined as per the clinical staff, essentially in an infant that looked unwell where bacterial sepsis could not be excluded clinically. Before the study, the staff at Kamuzu Central Hospital did not have routinely access to blood culture or other ancillary tests, IV fluids and gavage feeds were also most often not available due to limited resources and supplies. Available interventions include antibiotics, assistance with feeding and routine surgeries when necessary. Oxygen and continuous positive pressure ventilation were only

available to an extremely limited number of infants, on a limited basis when the hospital had electrical power. Invasive mechanical ventilation was not possible. During the study, ~~the authors~~investigators did not have access to information that could identify participants, with the exception of the consent forms ~~who were~~ kept under the responsibility of the local study PI (MCK). The study was administered and local research staff were hired through a partnership with the Parent and Child Health Initiative (PACHI), a Malawian non-profit non-governmental organization, which works in collaboration with the Ministry of Health.~~The study was approved by the University of British Columbia's Children's and Women's Research Ethics Board (#H16-02639) and the National Health Science Research Committee of Malawi (#17/8/1819).~~

Clinical data and blood samples collection: Each infant had an initial clinical evaluation by a healthcare worker (or staff, either a nurse or clinical officer). T~~, and t~~he presence of the following nine DS were prospectively collected at presentation, using electronic forms, on a tablet device: i) history of ~~poor feeding~~not feeding well (interviewing the parent/caregiver), entered as "feeding well", "unable to feed" or "uninterested in feeding" (combining the latter two categories), ii) fast breathing ≥60 bpm (with respiratory rate ~~(~~visually counted by attended clinician), iii) temperature instability, defined as axillary body temperature ~~Temperature instability was defined as a body temperature~~ <35.5°C or >38°C, ~~.~~ (measured using an electronic Welch Allyn SureTemp thermometer, model 692), iv) drowsiness/difficulty to wake, v) grunting, vi) severe chest recession, vii) central cyanosis, viii) movements only when stimulated or no movement at all and ix) convulsions (defined pragmatically by attending clinician). All infants also had a standardized aerobic blood culture (minimum 2 mL of blood) collected within 4 hours of presentation, using a rigorously implemented aseptic protocol (with in-training of all front-line healthcare providers prior to study initiation), as detailed (13). Infants also

had cerebrospinal fluid (CSF) and urine cultures collected at the discretion of the ~~based on the clinical judgement of the medical~~clinical staff ~~team,~~, availability of resources ~~such as lab personnel~~ and cost of the test to be paid by parents. ~~as clinically indicated~~. ~~Fast breathing was defined as a respiratory rate of ≥60 bpm.~~ ~~Temperature instability was defined as a body temperature <35.5°C or >38°C.~~

Blood cultures were processed into BACTEC PEDS Plus culture bottles incubated for 5 days in a BD BACTEC 9050 instrument. In keeping with a pragmatic design, the study did not attempt to enforce strict criteria to define suspected sepsis, DS or illness status. Identification of organisms from blood or CSF cultures was completed in-house using bioMerieux API, and BD Crystal kits at a local University of North Carolina-affiliated research facility, as described (12). ~~Culture results were made available to clinicians within 5 days.~~ Bacterial isolates that could be restored (~60% of isolates) were also analyzed for further confirmation of identity ~~(not shown)~~ on a Microflex LT MALDI-TOF mass spectrometer, using the BioTyper software version 3.1 (Bruker Daltonik, Germany). Culture results were made available to clinicians involved in the care of the infants.

<u>Outcome measures:</u> The primary outcomes of interest were *mortality*, and *culture-positive sepsis* defined as the bacterial growth of a known neonatal pathogen in blood or CSF culture sample. Micro-organisms not commonly considered as pathogens in term or near-term infants, such as coagulase-negative Staphylococcus, Micrococcus or Bacillus were excluded (14), but these potential contaminants were included in a sensitivity analysis using an alternate bacterial sepsis outcome "including potential contaminants" (**Supplemental Table 1**). In-hospital mortality was recorded until discharge home or transfer to another hospital facility. Data were considered missing when infants were transferred within 72 hours after initial admission to the hospital.

Statistical Analysis: In the parent study, the sample size was set conveniently at 500 infants over ~~2~~ two years, but the study needed to be stopped early, in April 2020 due to the COVID-19 pandemic, so in the end only 401 infants were enrolled~~. A total of 401 were included in the study~~. All infants originally enrolled in the parent study were analyzed. Demographic characteristics were reported descriptively using the mean/median with dispersion. The ~~following~~ nine aforementioned WHO DS were used as predictors~~: *not feeding well, convulsions, drowsy/difficult to wake, movements only when stimulated or no movements, fast breathing, grunting, severe chest recessions, temperature instability (hypo/hyperthermia), central cyanosis*~~. All quantitative variables were analyzed as such, except for fast breathing and temperature instability which were categorized according to WHO definitions (see above). Odds ratios were calculated for each DS and the main outcomes of bacterial sepsis or mortality. Logistic regressions were conducted between the number of DS and the main outcomes. In separate analyses, odds ratios were calculated using logistic regressions between mean DS (i.e., the number of DS divided by the available DS data) to determine the effect of missing DS data. Unadjusted logistic regression models, including demographic variables, were conducted to determine the predictive value of the DS. The predictive performance of DS for each outcome was assessed using the area under receiver operator characteristic curve (AUC-ROC). Sociodemographic variables associated with mortality, as well as their independent effects were analyzed using regression models. Only complete cases were used in the analysis for associations with individual DS, or the outcome "cumulative number of DS". However, analyses were also conducted using "mean number of DS", to account for missing values, with no significant differences in results. Statistical significance was considered at p-value <0.05 with no adjustment made for multiple testing in unadjusted models. Analyses were performed using R (version 4.1.0) and SPSS version 20 (IBM, Armonk, NY, USA).

Inclusivity in global research: Additional information regarding the ethical, cultural, and scientific considerations specific to inclusivity in global research is included in the **Supporting Information**.

Results

Study population: ~~All infants originally enrolled in the parent study were included, for a total of 401 infants enrolled in the original study and included in this analysis.~~ The majority of the 401 infants included in the study were term or late preterm infants, born vaginally, presented from home in their first week of post-natal life, and had roughly equal female/male distribution (**Table 1**). Most infants were reported by clinicians to ~~be~~ look ill at the time of initial presentation (69.3%; 278/401), 22.2% (89/401) were severely ill, and 8.5% appeared well (34/401). In total, 79.8% (320/401) presented at least one DS. Overall, 56.9% (228/401) of infants had been ill for at least 24 hours prior to initial presentation, and their parents had traveled a median distance of 17 kilometers, often by foot or taxi, to reach the nearest hospital. In total, 94/401 (23.4%) infants had received antibiotics prior to the blood culture, for a median of 2 hours (range 0.75 to 3.5 hours). The duration of antibiotic use in infants ranged from 1 to 30 days (**Table 1**). Few infants with negative blood culture had antibiotics discontinued after 48 hours (21/401, 5.2%).

Table 1: Socio-demographic characteristics of the study population.

**Participant characteristics**		**N with data**
Gestational age at birth (weeks), mean (SD)	37.1 (3.3)	397
Birth weight (gram), mean (SD)	2865 (785)	380
Female, no (%)	185 (46)	401

Admitted from, no (%)		401
Inborn (i.e., admitted from birth hospital)	161 (40)	
Transferred from another facility	224 (56)	
Home	16 (4)	
Post-natal age at presentation (days), median (IQR)	4 (2-16)	401
Mode of delivery, no (%)		398
~~Cesarean~~Caesarean section	101 (25.2)	
Vaginal delivery	297 (71.8)	
Duration of illness before presentation to hospital (hours), median (IQR)	24 (12-48)	341
Distance of travel to hospital (km), median (IQR)	17 (11-25)	342
Illness status, number on infants (%)		401
Well	34 (8.5)	
Ill	278 (69.3)	
Severely ill	89 (22.2)	
Duration of antibiotics (days), median (IQR)	6 (5-9)	401
Duration of hospital stay (days), median (IQR)	7 (5-9)	401
Danger Signs, number of infants (%)		
Not feeding well	171 (43.3)	395
Convulsions	28 (7.0)	401
Drowsy / Difficult to wake	33 (8.2)	401
Movements only when stimulated or no movements	26 (6.5)	401
Fast breathing >=60 per min	104 (27.1)	384

Grunting	30 (7.5)	401
Severe chest recessions	51 (12.7)	401
Temperature instability >38°C or <35.5°C	118 (32.7)	361
Central cyanosis	29 (7.2)	401

Outcome data: ~~Blood (n = 85/401) and CSF (n = 2/204) cultures were positive in 21.2% and 1% of the infants, respectively. However, about half (46 / 85) had bacterial types considered to be contaminants (e.g., *bacillus, micrococcus, corynebacterium* and *coagulase-negative staphylococcus* species and non-pathogenic *streptococcus* species such as *oralis*), so the actual~~ The proportion of infants with bacterial sepsis, considering only known pathogens in term and near-term infants, was ~~effectively 9.7~~10.2% (~~39~~41/401). Blood (39/401) and CSF (2/204) cultures were positive for pathogens in 9.7% and 1.0% of the infants, respectively. One infant had a contaminating blood culture, but grew unidentified gram-negative diplococci in the CSF, so was classified as bacterial sepsis with a pathogen. Of 230 infants under 7 days old, 21 (9.1%) had bacterial sepsis with a pathogen versus 20 of the 171 infants (11.7%; p=0.402 by chi-square test) ≥ 7 days old. ~~Excluding contaminants,~~ The proportion of gram-positive ~~bacteria~~ sepsis (~~53.8%;~~ 21/39) ~~were~~ and~~slightly more prominent than~~ gram-negative ~~bacteria~~ sepsis (~~46.2%; 18~~20/39) were similar among all infants (**Supplemental Table 1**), and were also similar between infants under 7 days (9 versus 12) and infants ≥ 7 days old (12 versus 8; p=0.272 by chi-square test, noting however the small sample size for comparison).~~.~~ ~~Among all infants with presumed sepsis,~~ The proportion of ~~pathogen~~ sepsis was also ~~more common in~~similar in ~~the~~ inborn infants (19~~7~~/161) ~~than~~ versus~~in the~~ infants transferred from another hospital facility (22/224) or admitted from home (~~22~~0/~~240~~16) ~~(p=0.029)~~. ~~The proportion of gram-positive sepsis was comparable between inborn~~

~~infants (10/240) versus in infants transferred from another hospital or home (11/161) (p=0.240).~~ Three infants were transferred before 72 hours from admission, so the mortality outcome was considered missing in these infants. A~~a~~mong the remaining infants, ~~infants in whom data were available, the~~ mortality was 9.7% (i.e., 39/398~~)~~)~~,~~, of which 61.5% [~~(~~24/39]~~)~~ died within 48 hours.~~h.~~

Associations with mortality and culture-positive outcomes: The following DS were significantly associated with mortality (**Table 2**): *not feeding well*, *convulsions*, *drowsy/difficult to wake*, *movements only when stimulated or no movements*, *grunting*, *severe chest recessions*, *central cyanosis*. In contrast, *fast breathing* or *temperature instability* were not significantly associated with mortality. The ~~cumulative~~ number of DS was also significantly associated with mortality (OR 1.75 [95%CI: 1.43 – 2.~~16~~17] per cumulative ~~additional~~ DS; p<0.001; AUC: 0.743) (**Supplemental Figure 1**). Admission weight was significantly associated with mortality, ~~,~~but neither age of the mother, gestational age, birth weight nor sex~~,~~ ~~was~~ were significantly associated with mortality (**Supplemental Table 2**). When adjusting for these co-variables, both the cumulative (OR 1.75 [95% CI: 1.39 - 2.23]; p<0.001) and mean number of DS (1.80 [95% CI: 1.44 – 2.30]; p<0.001) remained highly significantly associated with mortality ~~(OR 1.75 [95% CI: 1.39 - 2.23] and 1.80 [95% CI: 1.44 – 2.30], respectively;~~ (**Supplemental Table 2**).

Table 2: Associations between DS and mortality.

Variable	Odds ratio (OR)	95%CI	P value
Not feeding well	7.21	(3.27 - 18.2)	<0.001
Convulsions	3.52	(1.31 - 8.59)	0.008
Drowsy / Difficult to wake	5.04	(2.12 - 11.4)	<0.001
Movements only when stimulated or no movements	4.89	(1.88 - 11.9)	<0.001
Fast breathing >=60 per min	1.86	(0.87 - 3.85)	0.100
Grunting	3.95	(1.54 - 9.34)	0.002
Severe chest recessions	3.01	(1.34 - 6.37)	0.005
Temperature instability >38°C or <35.5°C	1.70	(0.75 - 3.75)	0.191
Central cyanosis	4.15	(1.62 - 9.87)	0.002

Bacterial sepsis with a known neonatal pathogen was significantly associated with mortality (OR 3.2907 [95%CI: 1.2387 – 76.3682]; p = 0.0085). However, the only DS significantly associated with bacterial sepsis when considering only known neonatal pathogens was *not feeding well* (OR 2.04 25 [95%CI: 1.1705 – 4.4406]; p=0.01378) (**Table 3**). Associations between convulsions, drowsiness, lethargy (i.e., movements only when stimulated or no movement), tachypnea, grunting, severe chest recession, temperature instability and central cyanosis were all non-significant (**Table 3**). Association was between cumulative number of DS and pathogen bacterial sepsis was also marginallynon-significant (OR 1.221 [95% CI: 1.0000.98 – 1.497]; p=0.04672) in regression models (**Supplemental Figure 2**). At discharge, clinical staff selected non-bacterial sepsis in 76.3% (306/401), bacterial sepsis in 15.5% (62/401) and “other non-sepsis syndromes” in 8.2% (33/401) of cases, from these options, for study purposes.

Table 3: Association between DS and bacterial sepsis when organism is a known pathogen.

Variable	Odds ratio (OR)	95%CI	P value
Not feeding well	2.~~04~~25	~~(~~1.~~05~~ 17 - 4.~~06~~44~~)~~	0.~~038~~017
Convulsions	0.65~~9~~	~~(~~0.10~~1~~ - 2.30~~45)~~	0.571~~626~~
Drowsy / Difficult to wake	0.~~91~~86	~~(~~0.20~~1~~ - 2.57~~73)~~	0.811~~886~~
Movements only when stimulated or no movements	1.65~~75~~	~~(~~0.46~~9~~ - 4.59~~90)~~	0.382~~27~~
Fast breathing >=60 per min	1.0~~1~~6	~~(~~0.49~~53~~ - 2.17~~38)~~	0.~~703~~868
Grunting	2.38~~54~~	~~(~~0.84~~9 -~~ – 5.89~~6.31)~~	0.07~~58~~7
Severe chest recessions	2.17~~1.61~~	~~(~~0.92~~62 -~~ – 4.74~~3.71)~~	0.~~288~~061
Temperature instability >38°C or <35.5°C	1.60~~80~~	~~(~~0.78~~86~~ - 3.24~~70)~~	0.19~~1~~3
Central cyanosis	1.01~~0.67~~	0.32~~(0.11~~ - 3~~2~~.03~~4)~~	0.~~588~~994

Associations when including potential contaminant blood cultures: When considering potential blood culture contaminants (e.g., *micrococcus*, *Corynebacterium*, *bacillus* and *coagulase-negative staphylococcus* species and non-pathogenic *streptococcus* species such as *S. oralis*), 86 infants had

positive blood or CSF cultures. ~~When including all positive blood cultures (**Supplemental Table 3**),~~ Bacterial culture positivity was not significantly associated with mortality (OR 1.~~77~~ 74 [95% CI: 0.82~~3~~ – 3.53~~6~~]; p=0.135~~23~~), or any DS when including contaminants (**Supplemental Table 3**). The ~~cumulative~~ number of DS was also not significantly associated with bacterial sepsis when including ~~positive cultures with potential bacterial~~ contaminants (OR 1.09 [95% CI: 0.93 – 1.28]; p=0.273; **Supplemental Figure 3**~~OR 1.21 [95% CI: 0.98 – 1.47]; p=0.0715~~).

Discussion

This study found that most WHO DS were significantly associated with mortality, but only one DS (i.e., not feeding well) as significantly associated with culture-proven bacterial sepsis. Additionally, the majority of DS were not associated with culture-proven bacterial sepsis in this LMIC neonatal cohort. The results held true whether only bacterial pathogens, or potential bacterial contaminants were considered. To the best of our knowledge, the current study is the first to pragmatically examine whether DS predict bacterial sepsis in infants. These findings were obtained ~~prospectively~~ in an LMIC setting where DS were prospectively identified by front line healthcare workers as they would normally do pragmatically outside a research protocol – we assumed that this study design would more closely reflects the real-life utility of DS. Few studies have attempted to determine how DS can predict bacterial sepsis caused by bacterial pathogens in ~~newborns or~~ young infants. A retrospective Brazilian study reviewed data on 83 sick neonates over a 12-month period, and came to the same conclusion that DS alone or in combination did not predict severe serious bacterial infection (15). The findings of the current study are also consistent with those of another study carried out in a primary care, rural setting in Bangladesh, India and Pakistan. In the latter study, clinical signs could not differentiate infants with viral or bacterial illnesses (16). The current study findings have implications for a hospital setting, but further research is needed to determine the generalizability of the results to other hospital contexts.

In addition to the main findings, the current study also found that only a minor proportion of infants with suspected sepsis showed positive blood culture. Only few other studies have prospectively estimated the incidence of bacterial sepsis in LMIC cohorts, and it is therefore useful to present the current findings in the context of those other studies. A hospital centre study in Delhi, India, included 13,530 neonates (all comers; mean GA±SD: 36.0±3.4 weeks) monitored daily for clinical signs of sepsis with per-protocol completion of a sepsis work-up (blood culture and lumbar puncture) prior to initiating antibiotics, and found a 14.3% incidence of culture-positive sepsis (17), which is similar to the findings of the current study. Another study in community settings in Madagascar, Senegal and Cambodia reported 8.2% culture-confirmed sepsis among 514 infants assessed for “possible serious bacterial infection” out of 3,688 neonates (18). One large study followed 12,622 live births up to 60 days of age in rural India between April 2002 and March 2005, with daily home visits and clinical assessment, and standardized blood and CSF cultures following enrollment of infants with suspected sepsis. It reported a 6.3% incidence of culture-confirmed sepsis (53 with positive blood cultures out of 842 infants assessed, excluding potential contaminants), for a population-level incidence of culture-confirmed sepsis of 6.7 per 1,000 births (19). Taken together, these findings ~~suggest tha~~provide robust data on the incidence of~~t~~ bacterial sepsis ~~may be uncommon in this age group even~~ in LMIC settings, to inform interventions and form the basis for future studies to measure the impact of those interventions. ~~s, when sepsis is clinically suspected.~~

Blood cultures are the gold standard for diagnosing bacterial sepsis in young infants (20). However, these tests lack sensitivity in neonates (21). In the current study we were careful to prescribe a minimum blood volume to maximize culture yield. Yet, a key question is whether we misestimate the proportion of bacterial sepsis in infants in whom cultures are negative? The Aetiology of Neonatal

Infection in South Asia (ANISA) study (22) also attempted to identify the cause of sepsis for 6022 cases of possible serious bacterial infections in infants in the first 60 days after birth, in a population of 63,114 livebirth). Bacterial and viral (e.g., respiratory syncytial virus) causes were identified in 16% and 12% of cases, respectively. For the remaining 72% cases, no pathogen could be identified despite systematic blood and respiratory cultures, which is consistent with findings from the current study.

Evidently the use of antibiotics in the current study was high, with the vast majority of infants receiving antibiotics for a minimum of five days during hospitalization, as per hospital practices. As in other LMIC settings, blood cultures were not routinely available at the study site prior to the study. As such, no approach had been developed to stop antibiotics after 48 hours given a negative blood culture. In another study in Thailand, the duration of antibiotic use was also high with durations of 15 and 8 days for culture-proven and culture-negative early-onset neonatal sepsis, respectively (23). These results highlight the ~~need~~ limits of having to rely on DS, and potential coercive effect of the lack of laboratory resources capable of providing basic laboratory test results to help “rule out” sepsis (24).

A strength of this study is ~~its prospective design~~its robust outcomes, with standardized blood culture procedures to ensure that the chances of contamination during blood sampling were minimized, and robust microbiological identification methods including clinical laboratory protocols on-site augmented by advanced MALDI-TOF species determination in another, North American clinical microbiology laboratory. The strong association between bacterial sepsis and mortality ~~suggest~~ provides reassurance~~that the~~ infants with positive cultures were correctly identified. However, a number of study limitations should be acknowledged. Although a sizeable proportion of the recruited infants presented from home, data were collected in a single hospital-type setting; as such, caution should be exercised when generalizing to other hospital settings or extrapolating to non-hospital primary care settings. For

logistical reasons CSF was collected from only approximately half of the infants, which may have underestimated the incidence of bacterial sepsis. Similarly, anaerobic blood cultures were not collected, which may have resulted in missed bacteremia. Some infants received antibiotics prior to blood culture, which may have ~~blunted~~ lowered bacterial sepsis estimates. Moreover, due to the extremely limited diagnostic capabilities of the study LMIC settings, in most cases, causes of deaths or alternate diagnoses could not be determined. Similarly, the study did not include non-bacterial testing, which precluded identification of viral sepsis syndromes.

In conclusion, our study validates the use of WHO DS to predict fatal outcomes, but not bacterial sepsis, in young infants. This study further emphasizes the need for better diagnostics for bacterial sepsis, to ensure that health gains continue to improve, in areas of the world where sepsis represents a huge burden and where antimicrobial resistance is also increasing at alarming rates (25).

Acknowledgments

We thank Bentry Tembo, Rhoda Chifisi and Blessings Chiluzi for help with recruitment and data collection, Charles Mwansambo, Patience Johane and the staff at the PACHI for administrative and operational support during the study, Robert Krysiak, Gerald Tegha and the staff of the University of North Carolina – Malawi lab for performing all bacterial cultures and identification, on-site, in Lilongwe, according to clinical laboratory standards, Dr. David Goldfarb for expert input into data interpretation, ~~and~~ Amber Johnson from the British Columbia Children's Hospital Microbiology Laboratory for secondary confirming bacterial isolates by mass spectrometry.

REFERENCES

1. GBD 2016 Causes of Death Collaborators. Global, regional, and national age-sex specific mortality for 264 causes of death, 1980-2016: a systematic analysis for the Global Burden of Disease Study 2016. Lancet. 2017;390(10100):1151-210.
2. Rudd KE, Johnson SC, Agesa KM, Shackelford KA, Tsoi D, Kievlan DR, et al. Global, regional, and national sepsis incidence and mortality, 1990-2017: analysis for the Global Burden of Disease Study. Lancet. 2020;395(10219):200-11.
3. Fleischmann-Struzek C, Goldfarb DM, Schlattmann P, Schlapbach LJ, Reinhart K, Kissoon N. The global burden of paediatric and neonatal sepsis: a systematic review. The Lancet Respiratory medicine. 2018;6(3):223-30.
4. Costelloe C, Metcalfe C, Lovering A, Mant D, Hay AD. Effect of antibiotic prescribing in primary care on antimicrobial resistance in individual patients: systematic review and meta-analysis. BMJ. 2010;340:c2096.
5. Klingenberg C, Kornelisse RF, Buonocore G, Maier RF, Stocker M. Culture-Negative Early-Onset Neonatal Sepsis - At the Crossroad Between Efficient Sepsis Care and Antimicrobial Stewardship. Front Pediatr. 2018;6:285.
6. Costello A. Integrated management of childhood illness. Lancet. 1997;350(9087):1266.
7. Young Infants Clinical Signs Study Group. Clinical signs that predict severe illness in children under age 2 months: a multicentre study. The Lancet. 2008;371(9607):135-42.
8. Opiyo N, English M. What clinical signs best identify severe illness in young infants aged 0-59 days in developing countries? A systematic review. Archives of disease in childhood. 2011;96(11):1052-9.

9. World Health Organization. Pocket Book of Hospital Care for Children: Guidelines for the Management of Common Childhood Illnesses. WHO Guidelines Approved by the Guidelines Review Committee. 2nd ed. Geneva, 2013.
10. Wynn JL, Polin RA. Progress in the management of neonatal sepsis: the importance of a consensus definition. Pediatr Res. 2018;83(1-1):13-5.
11. Clinical prediction of serious bacterial infections in young infants in developing countries. The WHO Young Infants Study Group. Pediatr Infect Dis J. 1999;18(10 Suppl):S23-31.
12. Popescu CR, Tembo B, Chifisi R, Cavanagh MMM, Lee AH, Chiluzi B, et al. Whole blood genome-wide transcriptome profiling and metagenomics next-generation sequencing in young infants with suspected sepsis in a low-and middle-income country: A study protocol. Gates Open Res. 2020;4:139.
13. Popescu C, Tembo B, Chifisi R, Cavanagh M, Chiluzi B, Lufesi N, et al. Procedure policies, data collection form and model consent form (supplemental material)~Improving the Early Diagnosis of Neonatal Sepsis in Malawi. In: Scholars Portal Dataverse, editor. 2 ed 2020.
14. Downie L, Armiento R, Subhi R, Kelly J, Clifford V, Duke T. Community-acquired neonatal and infant sepsis in developing countries: efficacy of WHO's currently recommended antibiotics--systematic review and meta-analysis. Archives of disease in childhood. 2013;98(2):146-54.
15. Ronfani L, Vilarim JN, Dragovich D, Bacalhau AF, Cattaneo A. Signs of severe bacterial infection in neonates. J Trop Pediatr. 1999;45(1):48-51.
16. Arvay ML, Shang N, Qazi SA, Darmstadt GL, Islam MS, Roth DE, et al. Infectious aetiologies of neonatal illness in south Asia classified using WHO definitions: a primary analysis of the ANISA study. The Lancet Global health. 2022;10(9):e1289-e97.
17. Study IotDNI. Characterisation and antimicrobial resistance of sepsis pathogens in neonates born in tertiary care centres in Delhi, India: a cohort study. The Lancet Global health. 2016;4(10):e752-60.

18. Huynh BT, Kermorvant-Duchemin E, Chheang R, Randrianirina F, Seck A, Ratsima EH, et al. Severe bacterial neonatal infections in Madagascar, Senegal, and Cambodia: A multicentric community-based cohort study. Plos Medicine. 2021;18(9).

19. Panigrahi P, Chandel DS, Hansen NI, Sharma N, Kandefer S, Parida S, et al. Neonatal sepsis in rural India: timing, microbiology and antibiotic resistance in a population-based prospective study in the community setting. J Perinatol. 2017;37(8):911-21.

20. Polin RA. Management of neonates with suspected or proven early-onset bacterial sepsis. Pediatrics. 2012;129(5):1006-15.

21. Popescu CR, Cavanagh MMM, Tembo B, Chiume M, Lufesi N, Goldfarb DM, et al. Neonatal sepsis in low-income countries: epidemiology, diagnosis and prevention. Expert Rev Anti Infect Ther. 2020;18(5):443-52.

22. Saha SK, Schrag SJ, El Arifeen S, Mullany LC, Shahidul Islam M, Shang N, et al. Causes and incidence of community-acquired serious infections among young children in south Asia (ANISA): an observational cohort study. Lancet. 2018;392(10142):145-59.

23. Kiatchoosakun P, Jirapradittha J, Sirikarn P, Laopaiboon M, Pattanittum P, Chandrakachorn W, et al. Early-Onset Neonatal Sepsis and Antibiotic Use in Northeast Thailand. Am J Perinatol. 2019;36(12):1295-303.

24. Lavoie PM, Popescu CR, Molyneux EM, Wynn JL, Chiume M, Keitel K, et al. Rethinking management of neonates at risk of sepsis. Lancet. 2019;394(10195):279-81.

25. Iregbu K, Dramowski A, Milton R, Nsutebu E, Howie SRC, Chakraborty M, et al. Global health systems' data science approach for precision diagnosis of sepsis in early life. Lancet Infect Dis. 2022;22(5):e143-e52.

General comment

• In the main method give the operation definition 'suspected sepsis' in newborns.

The definition of "suspected sepsis" was completely up to the attending staff, as it occurs pragmatically in the clinical setting. The following clarification has been made (line 108-110): This study was a pragmatic cohort study, and secondary analysis of data from a parent study that aimed to identify molecular signatures of bacterial sepsis in young infants with suspected sepsis, defined according to the attending staff's suspicion (12)."

We also added (line 149)): "In keeping with a pragmatic design, the study did not attempt to enforce strict criteria to define suspected sepsis, DS or illness status."

• Which types of bacteria were isolated from CSF?

As explained above, the two infants with positive CSF results had been mistakenly omitted, so this is now corrected in the revised manuscript. One child grew klebsiella pneumonia in the CSF (with a negative blood culture – was previously classified as such but is now reclassified as positive) and the other grew a coagulase-negative staph in the blood (previously classified as "contaminant") but grew an unidentified gram-negative diplococci in the CSF (that we now consider and reclassify as pathogen).

The analysis was revised accordingly, with minor changes in numbers, no major changes in overall conclusions or data interpretation.

• Some terms are inconsistent within the manuscript "young infants", "Newborns", "Neonates", "infants" It is good to define the study population and use it consistently throughout the manuscript.

Replaced newborns and neonates with infants.

• The study design, sample size (500 vs 401 vs) is not clearly mentioned.

We have clarified that: "In the parent study, the sample size was set conveniently at 500 infants over 2 years, but the study needed to be stopped in April 2020 due to the COVID-19 pandemic, so 401 infants were enrolled and included in the study."

• Consider replacing 'h' with 'hours' if it means so.

Line 61 and 212- 48h changed to 48 hours.

• Line # 144 "enrollment" Line # "approximatively" check the spelling.

Thank you for this comment, language was made uniform throughout in Canadian/British English (e.g., enrollment, instead of enrolment) as we were unable to find guidance on this on the PloS site.

• Mention the nine WHO danger signs in the clinical data and samples collection section.

We have added the "nine" to the objective line of the abstract. Most relevant to your point, we have clearly listed (and numbered) the nine DS in the Clinical data and blood sample collection Method section: "The presence of the following nine DS were prospectively collected at presentation, using electronic forms, on a tablet device: i) history of not feeding well (interviewing the parent/caregiver), entered as "feeding well", "unable to feed" or "uninterested in feeding" (combining the latter two categories), ii) fast breathing ≥60 bpm (with respiratory rate visually counted by attended clinician), iii) temperature instability, defined as axillary body temperature <35.5°C or >38°C, (measured using an electronic Welch Allyn SureTemp thermometer, model 692), iv) drowsiness/difficulty to wake, v) grunting, vi) severe chest recession, vii) central cyanosis, viii) movements only when stimulated or no movement at all and ix) convulsions (defined pragmatically by attending clinician)."

- Was the attempt made to isolate anaerobic bacteria? If not. include in the limitation.

No, we have not done anaerobic blood cultures – this has been added as a limitation.

- As the etiology of neonatal sepsis varies with age; I suggest considering presenting the result section by taking into account the age classification of newborns (< 7 days of age and 7-90 days of age).

We thank the reviewer for this suggestion. The numbers are small, but for whatever it is worth, we have added the observations that: "Of 230 infants under 7 days old, 21 (9.1%) had bacterial sepsis with a pathogen versus 20 of the 171 infants (11.7%; p=0.402 by chi-square test) ≥ 7 days old. The proportion of gram-positive sepsis (21/39) and gram-negative sepsis (20/39) were similar among all infants (**Supplemental Table 1**), and were also similar between infants under 7 days (9 versus 12) and infants ≥ 7 days old (12 versus 8; p=0.272 by chi-square test, noting however the small sample size for comparison)."

- What were the criteria to collect CSF?

It was left up to the discretion of the treating physician. "Infants also had cerebrospinal fluid (CSF) and urine cultures collected at the discretion of the clinical staff, availability of resources and cost of the test to be paid by parents." Line 146 and 147.

- How bacteria were identified after blood culture is not clear, for how long the blood culture was incubated before it was declared negative. There is no description of how CSF was cultured.

Blood cultures were incubated for 5 days before it was declared negative (which is fairly standard across microbiology lab in North America). We have added this (line 49).

"Identification of organisms from blood or CSF cultures was completed in-house using bioMerieux API, and BD Crystal kits at a local University of North Carolina-affiliated research facility, as described (12). Bacterial isolates that could be restored (~60% of isolates) were also analyzed for further confirmation of identity (not shown) on a Microflex LT MALDI-TOF mass

spectrometer, using the BioTyper software version 3.1 (Bruker Daltonik, Germany)."

Specific comments

• In the abstract section, I suggest separating methods and findings/results.

Done line 59.

• In the abstract it is written as "This prospective study evaluated nine DS in infants younger than 3 months…" Do newborns with the age of 3 months were included? **The comment is unclear to me.**

Hoping we understood well the reviewer's comment, we have clarified in the manuscript that the study enrolled infants under 3 months of age, including newborns, and used "infant" throughout.

• Line #93: "Over time, the use of DS has been extended to provide a reasonable basis for the initiation of empiric antibiotic treatment (9). However, these signs have neither been developed nor validated to diagnose sepsis of bacterial etiology." Is it correct to use reference #9 for the information given? Reference #9 looks like a guideline developed by WHO, is the WHO recommending the use of empiric antibiotic treatment without evidence?

Yes, the WHO is recommending to start antibiotics in a child that is showing DS, which was a major impetus for conducting this study. This has been extensively discussed in the literature and it is based on the rationale that, though these signs may be non-specific, starting antibiotics early in the children who truly have bacterial sepsis could be life-saving.

• In material and method section line #109 "study that aimed to identify molecular signatures of bacterial sepsis in young infants…" was molecular techniques used to diagnose sepsis?

Mainly RNA-sequencing (has been added to the sentence).

• Line #113-114: "…. infants were excluded if they had received antibiotics for >4 hours at the time of enrollment." What if they have received antibiotics treatment in the last two weeks?

The sentence has been reworded to clarify: "infants were excluded if they were already on antibiotics for >4 hours…"

• Line # 114-117: Out of 851, how many refused to participate, the condition was not permissive, and how many were included in the study?

Unfortunately, we do not have data on how many declined versus were not approached for consent.

• Line #119: "The Kamuzu Central Hospital the largest referral hospital in central Malawi, where approximatively 3,000 infants are admitted to its neonatal unit annually" needs reference

We are unaware of a published document we can reference for this. Admittedly, statistics in a poor country like Malawi are much less formally documented than can be in high income countries.

• Line # 128-129: "During the study, the authors did not have access to information that could identify participants…" if this is the case how lab results were communicated to the attending physician?

The blood culture results were communicated in real-time to attending staff but were not shared with the researchers. The sentence has been reworded to : "During the study, investigators did not have access to information that could identify participants, with the exception of the consent forms kept under the responsibility of the local study PI (MCK)."

Line # 150 "Culture results were made available to clinicians within 5 days." And also the recommended incubation period of blood culture is about a week, how culture result is available for the physician within 5 days?

Actually, we truly we did not capture the time between blood culture and resulting to clinicians, so more appropriately, the sentence has been reworded to: Culture results were made available to clinicians involved in the care of the infants."

• Line # 193-195: "In total, 94/401 (23.4%) infants had received antibiotics prior to the blood culture, for a median of 2 hours (range 0.75 to 3.5 195 hours)" This could have affected the findings.

We suspect that this would be unlikely to negate blood culture results in most cases. However, fairly, we have added the limitation that: "Some infants received antibiotics prior to blood culture, which may have lowered bacterial blood estimates." Our sense also is that many other studies had a similar problem but we were unable to find report of this information in most of these studies.

• Some data were collected from the baby's mother for example gestational age, and mode of delivery. Was consent sought from mothers?

Yes, consent was obtained from the mothers to obtain these data.

• Table 1 needs to be improved. Why values in the column 'N with data' are variable given the total sample size is 401? Fifth row what does 'inborn' mean?

Inborn means that the child was admitted from the birth hospital (i.e., never went home after delivery). As for the N with data, it is good practice to report missing data for every variable. In reality, all studies have missing data. The N refers to the number of babies with data for each specific variable.

• Table 1: Why capturing "Illness status, number on infants" is needed provided that the study population are newborns suspected of sepsis (ill) visiting the hospital.

Not all infants were ill at the time of initiation of antibiotics. Some were showing obviously some symptoms but were deemed general “well” by the attending staff (i.e., fever in an otherwise well baby).

• Line # 202: The contamination rate is somewhat high “However, about half (46 / 85) had bacterial types considered to be contaminants” If the blood specimen were collected aseptically they may be true pathogens.

Even in the best conditions when an aseptic technique is supposed to be used, would contaminants be expected even in a child with suspected sepsis. The literature often considers some bacteria very likely to be contaminants in a near term/term baby without risk factors. This is debatable but is what we have discussed with our microbiologist experts. It is also why we conducted the sensitivity analysis with all blood culture (a recognizable strength in our study).

• Line 203-204: “…and non-pathogenic streptococcus species such as oralis)…” you may rewrite as ‘….and non-pathogenic Streptococcus species such as S. oralis)…’ Consider scientific name during writing the name of bacteria.

Thank you, we have made this correction.

• Table 2: it is good if a column is added to show frequency and proportion in the ‘yes’ and ‘No’ categories for all variables. Doing so will make the reader see the prevalence in each cell.

• Table 3: it is good if a column is added to show frequency and proportion in yes and No category for all variables.

We thank the reviewer for these two suggestions, but the data is already in Table 1. Would it need to be repeated for each table? We hope this is acceptable to the reviewer, for the sake of simplicity.

• Tables 2 and 3: I don’t see the importance of using (), it can be omitted.

Change made

• Discussion lines # 277-278: “Taken together, these findings suggest that bacterial sepsis may be uncommon in this age group even in LMICs when sepsis is clinically suspected.” This is an area of controversy in LMICs and sub-Saharan African countries. Proxy studies and systematic reviews and meta-analyses reported a high burden of neonatal sepsis in these regions. Strong recommendations should be forwarded to concerned bodies so that similar studies (like the present study) using robust diagnostic methods to be conducted in the region. This will assist in device appropriate prevention strategies for the regions which may include vaccines and measure the impact of the prevention strategy. Without knowing the real burden of neonatal sepsis along with its etiology we may not objectively measure the impact of the prevention strategy to be used.

We thank the reviewer for this comment. We agree that it could be controversial to qualify the incidence as being “low” (or not). Based on the reviewer’s suggestion we have reworded the sentence: Taken together, these findings provide robust data on the incidence of bacterial sepsis in LMIC settings, to inform interventions and form the basis for future studies to measure the impact of those interventions.”

• Discussion: line# 302-304: “The strong association between bacterial sepsis and mortality suggest that the infants with positive cultures were correctly identified” Is there a finding which supports this claim?

What we mean here is that if the bacterial cultures were completely non-specific, we would not expect a difference in mortality between culture positive and culture-negative children. Perhaps it is clearly now when reworded as: The strong association between bacterial sepsis and mortality provides reassurance infants with positive cultures were correctly identified.”
