# Supplementary material for: World Health Organization Danger Signs to predict bacterial sepsis in young infants: A pragmatic cohort study
Source: PLOS Glob Public Health. 2023 Nov 21;3(11):e0001990. doi: 10.1371/journal.pgph.0001990 (PMC10662722; doi:10.1371/journal.pgph.0001990)
Supplement: Attachment — Submitted filename: PGPH-D-23-00809_R2_reviewer_R2.pdf [file pgph.0001990.s013.pdf]

PLOS Global Public Health

World Health Organization Danger Signs to predict bacterial sepsis in young infants: A pragmatic cohort study

--Manuscript Draft--

**Manuscript Number:**	PGPH-D-23-00809R2
**Article Type:**	Research Article
**Full Title:**	World Health Organization Danger Signs to predict bacterial sepsis in young infants: A pragmatic cohort study
**Short Title:**	Prediction of bacterial sepsis in infants.
**Corresponding Author:**	Pascal M. Lavoie, MDCM PhD FRCPC<br>The University of British Columbia<br>Vancouver, CANADA
**Order of Authors:**	Omolabake Akinseye, MBBS
	Constantin R. Popescu, MD, MSc
	Msandeni Chiume-Kayuni MMED Peads, MSc
	Michael A. Irvine, PhD
	Norman Lufesi DCM, MPhil
	Tisungane Mvalo, MBBS, MMED
	Niranjan Kissoon, MD
	Matthew O. Wiens, PharmD, PhD
	Pascal M. Lavoie, MDCM PhD FRCPC
**Keywords:**	Neonate; sepsis; Bacteremia; diagnosis; Epidemiology; low- and middle-income countries
**Abstract:**	Background: Bacterial sepsis is generally a major concern in ill infants. To help triaging decisions by front-line health workers in these situations, the World Health Organization (WHO) has developed danger signs (DS). The objective was to evaluate the extent to which nine DS predict bacterial sepsis in young infants presenting with suspected sepsis in a low-income country setting.<br>Methodology: This study pragmatically evaluated nine DS in infants younger than 3 months with suspected sepsis in a regional hospital in Lilongwe, Malawi, between June 2018 and April 2020. Main outcomes were positive blood or cerebrospinal fluid (CSF) cultures for neonatal pathogens, and mortality.<br>Findings/Results: Among 401 infants (gestational age [mean ± SD]: 37.1±3.3 weeks, birth weight 2865±785 grams), 41 had positive blood or CSF cultures for a neonatal pathogen. In-hospital mortality occurred in 9.7% of infants overall (N=39/401), of which 61.5% (24/39) occurred within 48 hours of admission. Mortality was higher in infants with bacterial sepsis compared to other infants (22.0% [9/41] versus 8.3% [30/360]; p=0.005). All DS were associated with mortality except for temperature instability and tachypnea, whereas none of the DS were significantly associated with bacterial sepsis, except for “unable to feed” (OR 2.25; 95%CI: 1.17 – 4.44; p=0.017). The number of DS predicted mortality (OR: 1.75; 95%CI: 1.43 – 2.17; p<0.001; AUC: 0.756), but was marginally associated with positive cultures with a neonatal pathogen (OR 1.22; 95%CI: 1.00 – 1.49; p=0.046; AUC: 0.743). The association between number of DS and mortality remained significant after adjusting for admission weight, the only statistically significant co-variable (OR 1.75 [95% CI: 1.39 – 2.23]; p<0.001). Considering all positive cultures including potential bacterial contaminants resulted a non-significant association between number of DS and sepsis (OR 1.09 [95% CI: 0.93 – 1.28]; p=0.273).<br>Conclusion/Significance: DS predicted death, but was marginally associated with culture-positive pathogen sepsis in a regional hospital setting. These data imply that the incidence of bacterial sepsis and attributable mortality in infants based on clinical signs may not be accurate, in LMIC settings.

Powered by Editorial Manager® and ProduXion Manager® from Aries Systems Corporation

Additional Information:	
**Question**	**Response**
**Financial Disclosure**<br><br>Enter a financial disclosure statement that describes the sources of funding for the work included in this submission and the role the funder(s) played. This includes grants and any commercial funding of the work or authors.<br><br>This statement will be typeset if the manuscript is accepted for publication.<br><br>*Please review the submission guidelines and the instructions link below for detailed requirements and guidance.*	This study was support by a grant from Grand Challenges Canada (R-ST-POC-1707-04630). PML was supported by the BC Children's Hospital Research Foundation through the Investigator Grant Award Program. CRP was funded by a Sue Carruthers Graduate Studentship from the BC Children's Hospital Foundation.
**Competing Interests**<br><br>On behalf of all authors, disclose any competing interests that could be perceived to bias this work.<br><br>This statement will be typeset if the manuscript is accepted for publication.<br><br>*Please review the instructions link below and PLOS Global Public Health's competing interests policy to determine what information must be disclosed at submission.*	The authors have declared that no competing interests exist.
**Data Availability**<br><br>Before publication, Authors are required to make fully available and without restriction all data underlying their findings. Please see our PLOS Data Policy page for detailed information on this policy.<br><br>A **Data Availability Statement**, detailing	Dataset has been uploaded.

where the data can be accessed, is required at first submission. Insert your Data Availability Statement in the box below.<br><br>Please see the data reporting section of our submission guidelines for instructions on what you need to include in your Data Availability Statement.<br><br>This statement will be typeset if the manuscript is accepted for publication.<br><br>*PLOS allows rare exemptions to address legal and ethical concerns. If you have legal or ethical restrictions, please use the box below to detail these in full sentences for the Journal team to consider.*	

Manuscript
Click here to access/download;Manuscript;WHO Manuscript - Revised Oct 4 2023.docx

World Health Organization Danger Signs to predict bacterial sepsis in young infants: A pragmatic cohort study

Omolabake Akinseye, MBBS[1,#], Constantin R. Popescu, MD, MSc[2,3,#], Msandeni Chiume-Kayuni MMED Peads, MSc[4,5], Michael A. Irvine, PhD[6,7], Norman Lufesi DCM, MPhil[8], Tisungane Mvalo, MBBS, MMED[9,10], Niranjan Kissoon, MD, MCCM[1,2], Matthew O. Wiens, PharmD, PhD[11], Pascal M. Lavoie, MDCM, PhD[1,2,*]

[#] contributed equally as co-first authors

Affiliations:
[1] Department of Pediatrics, Faculty of Medicine, University of British Columbia, Vancouver, Canada
[2] British Columbia Children's Hospital Research Institute, Vancouver, Canada
[3] Department of Pediatrics, Université Laval, Québec, Canada
[4] Department of Pediatrics, Kamuzu Central Hospital, Lilongwe, Malawi
[5] Kamuzu University of Health Sciences, Lilongwe, Malawi
[6] British Columbia Centre for Disease Control, Vancouver, Canada
[7] Faculty of Health Sciences, Simon Fraser University, Burnaby, Canada
[8] Department of Curative and Medical Rehabilitation, Ministry of Health, Lilongwe, Malawi
[9] University of North Carolina Project Malawi, Lilongwe, Malawi
[10] Department of Pediatrics, University of North Carolina at Chapel Hill School of Medicine, Chapel Hill, NC, USA
[11] Department of Anesthesiology, Pharmacology & Therapeutics, Faculty of Medicine, University of British Columbia, Vancouver, Canada

***Corresponding author:** Dr. Pascal M. Lavoie
BC Children's Hospital Research Institute
4th Floor, Translational Research Building
950 West 28th Avenue
Vancouver, British Columbia V5Z 4H4, Canada
Email: plavoie@bcchr.ca
Fax: +1-604-875-3106

Short title: Prediction of bacterial sepsis in infants.

Conflict of Interest Statement: The authors declare no conflicts of interest.

Funding Support: This study was support by a grant from Grand Challenges Canada (R-ST-POC-1707-04630). PML was supported by the BC Children's Hospital Research Foundation through the Investigator Grant Award Program. CRP was funded by a Sue Carruthers Graduate Studentship from the BC Children's Hospital Foundation. The other authors received no additional funding.

Role of Funder/Sponsor: The funder/sponsor did not participate in the work.

Abbreviations: WHO: World Health Organization; DS: danger signs; LMICs: low and middle-income countries; CSF: cerebrospinal fluid; AUC-ROC: Area Under Curve – Receiver Operator Characteristics; bpm: breaths per minute.

Abstract

Background: Bacterial sepsis is generally a major concern in ill infants. To help triaging decisions by front-line health workers in these situations, the World Health Organization (WHO) has developed danger signs (DS). The objective was to evaluate the extent to which nine DS predict bacterial sepsis in young infants presenting with suspected sepsis in a low-income country setting.

Methodology: This study pragmatically evaluated nine DS in infants younger than 3 months with suspected sepsis in a regional hospital in Lilongwe, Malawi, between June 2018 and April 2020. Main outcomes were positive blood or cerebrospinal fluid (CSF) cultures for neonatal pathogens, and mortality.

Findings/Results: Among 401 infants (gestational age [mean ± SD]: 37.1±3.3 weeks, birth weight 2865±785 grams), 41 had positive blood or CSF cultures for a neonatal pathogen. In-hospital mortality occurred in 9.7% of infants overall (N=39/401), of which 61.5% (24/39) occurred within 48 hours of admission. Mortality was higher in infants with bacterial sepsis compared to other infants (22.0% [9/41] versus 8.3% [30/360]; p=0.005). All DS were associated with mortality except for temperature instability and tachypnea, whereas none of the DS were significantly associated with bacterial sepsis, except for "unable to feed" (OR 2.25; 95%CI: 1.17 – 4.44; p=0.017). The number of DS predicted mortality (OR: 1.75; 95%CI: 1.43 – 2.17; p<0.001; AUC: 0.756), but was marginally associated with positive cultures with a neonatal pathogen (OR 1.22; 95%CI: 1.00 – 1.49; p=0.046; AUC: 0.743). The association between number of DS and mortality remained significant after adjusting for admission weight, the only statistically significant co-variable (OR 1.75 [95% CI: 1.39 – 2.23]; p<0.001). Considering all positive cultures including potential bacterial contaminants resulted a non-significant association between number of DS and sepsis (OR 1.09 [95% CI: 0.93 – 1.28]; p=0.273).

Conclusion/Significance: DS predicted death, but was marginally associated with culture-positive pathogen sepsis in a regional hospital setting. These data imply that the incidence of bacterial sepsis and attributable mortality in infants based on clinical signs may not be accurate, in LMIC settings.

Introduction

Three million children die each year worldwide in their first month of age, with a significant proportion of these deaths occurring during the first week(1). Severe infections (including sepsis, pneumonia, diarrhea and viral diseases) are common causes of serious illnesses in young infants (2). Up to 25% of cases in low- and middle-income countries (LMICs) have identified bacterial causes (3). However, the lack of diagnostic resources, particularly in LMICs makes it difficult to accurately quantify this burden. This gap has enormous implications in terms of implementing policies and guidelines for the use of antimicrobials to prevent these deaths (1). At the individual level, identifying bacterial causes for sepsis is crucial to prevent fatalities and guide the correct empiric antibiotic therapy in the context of rising antimicrobial resistance. Indeed, the misuse, or even the overuse, of antibiotics carries a risk of subsequent treatment failure due to colonization by resistant bacteria (4, 5).

To assist front-line health workers, the World Health Organization (WHO) and United Nations International Children's Emergency Fund developed algorithms in the early 1990s to help triage interventions for sick children (6). This Integrated Management of Childhood Illness (IMCI) strategy included the identification of clinical signs to alert the need for escalation of care, later termed Danger Signs (DS) (6). Subsequently, the Young Infant Clinical Signs Study Group carried out a multi-centered study in six LMICs and found that seven signs (i.e., history of difficulty feeding, grunting, convulsions, movement only when stimulated, respiratory rate ≥60 breaths per minute (bpm), severe chest indrawing, temperature below 35.5°C or above 37.5°C, prolonged capillary refill, cyanosis and stiff limbs) best predicted the need for higher level of care, with a sensitivity of 74% to 85%, and specificity of 75% to 79%, depending on infants' age (i.e., 0 to 6 days versus 7 to 59 days old) (7). A systematic review of five studies on 17,506 infants found similar results (8).

Over time, the use of DS has been extended to provide a reasonable basis for the initiation of empiric antibiotic treatment (9). However, these signs have neither been developed nor validated to diagnose sepsis of bacterial etiology. Neonatal disorders such as transient tachypnea of the newborn, hypoglycemia, hyaline membrane disease, birth asphyxia, and viral infections (to mention only a few), present like bacterial sepsis (10). Hence, a key question is whether DS can reasonably identify young infants who truly benefit from antibiotic treatment. Only a few studies have attempted to answer this question, but certainly none that have, to the best of our knowledge, been conducted prospectively, in a pragmatic setting where clinicians, not research staff, pragmatically reported DS (11).

The objective of this study was to determine the extent to which WHO DS predict bacterial sepsis in young infants, in a LMIC hospital setting where front-line health workers were asked to assess and record the presence of DS in infants who presented with suspected sepsis.

Materials and Methods

Study approval: The study was approved by the National Health Science Research Committee of Malawi (#17/8/1819) and the University of British Columbia's Children's and Women's Research Ethics Board (#H16-02639). The study was supported by the chair of the Department of Pediatrics at Kamuzu Central Hospital and discussed with leaders from the Pediatric and Child Health Association (PACHA), UNICEF Malawi and the Ministry of Health of Malawi.

Study design, setting & population: This study was a pragmatic cohort study, and secondary analysis of data from a parent study that aimed to identify molecular RNA-sequencing signatures of bacterial sepsis in young infants with suspected sepsis (12). In the parent study, infants below 3 months of age with suspected sepsis, defined according to the attending staff's clinically suspicion, were sequentially

enrolled “around the clock” at the time they presented to the neonatal or pediatric units at Kamuzu Central Hospital in Lilongwe, Malawi between June 5th, 2018, and April 6th, 2020 (including recruitment, exposure and data collection). To minimize bias towards false-negative blood culture, infants were excluded if they had been on antibiotics more than 4 hours prior to the cultures. About 851 infants presented with suspected sepsis and the vast majority were not enrolled because staff were unable to get consent – either parents declined, or were not approached for consent due to lack of time and/or competing clinical demands. Written informed consent was obtained from parents/guardian of each participant, in English or Chichewa (main local language). Enrolled infants were followed during hospitalization at Kamuzu Central Hospital, until transferred to another facility or discharged home.

The Kamuzu Central Hospital the largest referral hospital in central Malawi, where approximatively 3,000 infants are admitted to its neonatal unit annually. “Suspected sepsis” was defined as per the clinical staff, essentially in an infant that looked unwell where bacterial sepsis could not be excluded clinically. Before the study, the staff at Kamuzu Central Hospital did not have routinely access to blood culture or other ancillary tests, IV fluids and gavage feeds were also most often not available due to limited resources and supplies. Available interventions include antibiotics, assistance with feeding and routine surgeries when necessary. Oxygen and continuous positive pressure ventilation were only available to an extremely limited number of infants, on a limited basis when the hospital had electrical power. Invasive mechanical ventilation was not possible. During the study, investigators did not have access to information that could identify participants, except for the consent forms which were kept under the responsibility of the local study PI (MCK). The study was administered and local research staff were hired through a partnership with the Parent and Child Health Initiative (PACHI), a Malawian non-profit non-governmental organization, which works in collaboration with the Ministry of Health.

Clinical data and blood samples collection: Each infant had an initial clinical evaluation by a healthcare worker (or staff, either a nurse or clinical officer). The presence of the following nine DS were prospectively collected at presentation, using electronic forms, on a tablet device: i) history of not feeding well (interviewing the parent/caregiver), entered as “feeding well”, “unable to feed” or “uninterested in feeding” (combining the latter two categories), ii) fast breathing ≥60 bpm (with respiratory rate visually counted by attended clinician), iii) temperature instability, defined as axillary body temperature <35.5°C or >38°C, (measured using an electronic Welch Allyn SureTemp thermometer, model 692), iv) drowsiness/difficulty to wake, v) grunting, vi) severe chest recession, vii) central cyanosis, viii) movements only when stimulated or no movement at all and ix) convulsions (defined pragmatically by attending clinician). Additionally, clinical staff had to report whether they felt the infant was “well”, “ill” or “severely ill” at the time of assessment. However, in keeping with a pragmatic design, the study did not attempt to enforce strict criteria for illness status, or for to define suspected sepsis or the presence or absence of DS. All infants also had a standardized aerobic blood culture (minimum 2 mL of blood) collected within 4 hours of presentation, using a rigorously implemented aseptic protocol (with in-training of all front-line healthcare providers prior to study initiation), as detailed (13). Cerebrospinal fluid (CSF) cultures were collected by clinical staff, whenever they felt it was clinically indicated, at their discretion, based on availability of supplies, and the cost of the test that had to be paid by parents. No urine cultures were performed.

Blood cultures, were paid by the study, and processed into BACTEC PEDS Plus culture bottles incubated for 5 days in a BD BACTEC 9050 instrument. Identification of organisms from blood or CSF cultures was completed in-house using bioMerieux API, and BD Crystal kits at a local University of North Carolina-affiliated research facility, as described (12). Bacterial isolates that could be restored (~60% of isolates) were also analyzed for further confirmation of identity on a Microflex LT MALDI-

TOF mass spectrometer, using the BioTyper software version 3.1 (Bruker Daltonik, Germany). Culture results were made available to clinicians involved in the care of the infants.

Outcome measures: The primary outcomes of interest were *mortality*, and *culture-positive sepsis* defined as the bacterial growth of a known neonatal pathogen in blood or CSF culture sample. Micro-organisms not commonly considered as pathogens in term or near-term infants, such as *coagulase-negative Staphylococcus*, *Micrococcus*, *Corynebacterium*, *Bacillus species*, or streptococcus species such as *S. oralis* were considered as potential contaminants and were excluded from the main bacterial sepsis outcome (14), but these potential contaminants were included in a sensitivity analysis using an alternate bacterial sepsis outcome "including potential contaminants" (**Supplemental Table 1**). In-hospital mortality was recorded until discharge home or transfer to another hospital facility. Data were considered missing when infants were transferred within 72 hours after initial admission to the hospital.

Statistical Analysis: In the parent study, the sample size was set on the original molecular signature aim, at 500 infants over two years, assuming a 20% incidence of bacterial sepsis and expecting to identify between 10 and 50 differentially expressed molecular gene markers (12). However, the study needed to be stopped early, in April 2020 due to the COVID-19 pandemic, so in the end only 401 infants were enrolled. All infants originally enrolled in the parent study were analyzed. Demographic characteristics were reported descriptively using the mean/median with dispersion. The nine aforementioned WHO DS were used as predictors. All quantitative variables were analyzed as such, except for fast breathing and temperature instability which were categorized according to WHO definitions (see above). Odds ratios were calculated for each DS and the main outcomes of bacterial sepsis or mortality. Logistic regressions were conducted between the number of DS and the main outcomes. In separate analyses, odds ratios were calculated using logistic regressions between mean

DS (i.e., the number of DS divided by the available DS data) to determine the effect of missing DS data. Unadjusted logistic regression models, including demographic variables, were conducted to determine the predictive value of the DS. The predictive performance of DS for each outcome was assessed using the area under receiver operator characteristic curve (AUC-ROC). Sociodemographic variables associated with mortality, as well as their independent effects were analyzed using regression models. Only complete cases were used in the analysis for associations with individual DS, or the outcome "cumulative number of DS". However, analyses were also conducted using "mean number of DS", to account for missing values, with no significant differences in results. Statistical significance was considered at p-value <0.05 with no adjustment made for multiple testing in unadjusted models. Analyses were performed using R (version 4.1.0) and SPSS version 20 (IBM, Armonk, NY, USA).

Inclusivity in global research: Additional information regarding the ethical, cultural, and scientific considerations specific to inclusivity in global research is included in the **Supporting Information**.

Results

Study population: The majority of the 401 infants included in the study were term or late preterm infants, born vaginally, presented from home in their first week of post-natal life, and had roughly equal female/male distribution (**Table 1**). Most infants were reported by clinicians to look ill at the time of initial presentation (69.3%; 278/401), 22.2% (89/401) were severely ill, and 8.5% appeared well (34/401). In total, 79.8% (320/401) presented at least one DS. Overall, 56.9% (228/401) of infants had been ill for at least 24 hours prior to initial presentation, and their parents had traveled a median distance of 17 kilometers, often by foot or taxi, to reach the nearest hospital. In total, 94/401 (23.4%) infants had received antibiotics prior to the blood culture, for a median of 2 hours (range 0.75 to 3.5

hours). The duration of antibiotic use in infants ranged from 1 to 30 days (**Table 1**). Few infants with negative blood culture had antibiotics discontinued after 48 hours (21/401, 5.2%).

Table 1: Socio-demographic characteristics of the study population.

**Participant characteristics**		**N with data**
Gestational age at birth (weeks), mean (SD)	37.1 (3.3)	397
Birth weight (gram), mean (SD)	2865 (785)	380
Female, no (%)	185 (46)	401
Admitted from, no (%)		401
Inborn (i.e., admitted from birth hospital)	161 (40.1)	
Transferred from another facility	224 (55.8)	
Home	16 (4.0)	
Post-natal age at presentation (days), median (IQR)	4 (2-16)	401
Mode of delivery, no (%)		398
Caesarean section	101 (25.2)	
Vaginal delivery	297 (71.8)	
Duration of illness before presentation to hospital (hours), median (IQR)	24 (12-48)	341
Distance of travel to hospital (km), median (IQR)	17 (11-25)	342
Illness status, number on infants (%)		401
Well	34 (8.5)	
Ill	278 (69.3)	
Severely ill	89 (22.2)	

Total duration of antibiotics (days), median (IQR)	6 (5-9)	401
Duration of hospital stay (days), median (IQR)	7 (5-9)	401
Danger Signs, number of infants (%)		
Not feeding well	171 (43.3)	395
Convulsions	28 (7.0)	401
Drowsy / Difficult to wake	33 (8.2)	401
Movements only when stimulated or no movements	26 (6.5)	401
Fast breathing >=60 per min	104 (27.1)	384
Grunting	30 (7.5)	401
Severe chest recessions	51 (12.7)	401
Temperature instability >38°C or <35.5°C	118 (32.7)	361
Central cyanosis	29 (7.2)	401

Outcome data: The proportion of infants with bacterial sepsis, considering only known pathogens in term and near-term infants, was 10.2% (41/401). Blood (39/401) and CSF (2/204) cultures were positive for pathogens in 9.7% and 1.0% of the infants, respectively. Of 230 infants under 7 days old, 21 (9.1%) had bacterial sepsis with a pathogen versus 20 of the 171 infants (11.7%; p=0.402 by chi-square test) ≥ 7 days old. The proportion of gram-positive sepsis (21/39) and gram-negative sepsis (20/39) were similar among all infants (**Supplemental Table 1**), and were also similar between infants under 7 days (9 versus 12) and infants ≥ 7 days old (12 versus 8; p=0.272 by chi-square test, noting however the small sample size for comparison). The proportion of sepsis was also similar in inborn infants (19/161) versus infants transferred from another hospital facility (22/224) or admitted from home (0/16). Three infants were transferred before 72 hours from admission, so the mortality outcome

was considered missing in these infants. Among the remaining infants, mortality was 9.7% (i.e., 39/398), of which 61.5% [24/39] died within 48 hours.

Associations with mortality and culture-positive outcomes: The following DS were significantly associated with mortality (**Table 2**): *not feeding well*, *convulsions*, *drowsy/difficult to wake*, *movements only when stimulated or no movements*, *grunting*, *severe chest recessions*, *central cyanosis*. In contrast, *fast breathing* or *temperature instability* were not significantly associated with mortality. The number of DS was also significantly associated with mortality (OR 1.75 [95%CI: 1.43 – 2.17] per cumulative DS; p<0.001; AUC: 0.743) (**Supplemental Figure 1**). Admission weight was significantly associated with mortality, but neither age of the mother, gestational age, birth weight nor sex were significantly associated with mortality (**Supplemental Table 2**). When adjusting for these co-variables, both the cumulative (OR 1.75 [95% CI: 1.39 – 2.23]; p<0.001) and mean number of DS (1.80 [95% CI: 1.44 – 2.30]; p<0.001) remained highly significantly associated with mortality (**Supplemental Table 2**).

Table 2: Associations between DS and mortality.

Variable	Odds ratio (OR)	95%CI	P value
Not feeding well	7.21	3.27 – 18.2	<0.001*
Convulsions	3.52	1.31 – 8.59	0.008*
Drowsy / Difficult to wake	5.04	2.12 – 11.4	<0.001*
Movements only when stimulated or no movements	4.89	1.88 – 11.9	<0.001*
Fast breathing >=60 per min	1.86	0.87 – 3.85	0.100
Grunting	3.95	1.54 – 9.34	0.002*
Severe chest recessions	3.01	1.34 – 6.37	0.005*
Temperature instability >38°C or <35.5°C	1.70	0.75 – 3.75	0.191
Central cyanosis	4.15	1.62 – 9.87	0.002*

*Significant ORs (p<0.05)

Mortality was higher in infants with bacterial sepsis with a known neonatal pathogen compared to other infants (22.0% [9/41] versus 8.3% [30/360]; p=0.005). Bacterial sepsis was significantly associated with mortality (OR 3.07 [95%CI: 1.28 – 6.82]; p = 0.008). However, the only DS significantly associated with bacterial sepsis when considering only known neonatal pathogens was *not feeding well* (OR 2.25 [95%CI: 1.17 – 4.44]; p=0.017 (**Table 3**); 61.0% [25/41] of the children with bacterial sepsis were not feeding well versus 41.2% [146/354] of the other children). Associations between convulsions, drowsiness, lethargy (i.e., movements only when stimulated or no movement), tachypnea, grunting, severe chest recession, temperature instability and central cyanosis were all non-significant (**Table 3**). Association was between cumulative number of DS and bacterial sepsis was marginally significant (OR 1.22 [95% CI: 1.00 – 1.49]; p=0.046) (**Supplemental Figure 2**). At discharge, clinical

staff selected non-bacterial sepsis in 76.3% (306/401), bacterial sepsis in 15.5% (62/401) and "other non-sepsis syndromes" in 8.2% (33/401) of cases, from these options, for study purposes.

Table 3: Association between DS and bacterial sepsis when organism is a known pathogen.

Variable	Odds ratio (OR)	95%CI	P value
Not feeding well	2.25	1.17 – 4.44	0.017*
Convulsions	0.65	0.10 – 2.30	0.571
Drowsy / Difficult to wake	0.86	0.20 – 2.57	0.811
Movements only when stimulated or no movements	1.65	0.46 – 4.59	0.382
Fast breathing >=60 per min	1.06	0.49 – 2.17	0.868
Grunting	2.38	0.84 – 5.89	0.077
Severe chest recessions	2.17	0.92 – 4.74	0.061
Temperature instability >38°C or <35.5°C	1.60	0.78 – 3.24	0.193
Central cyanosis	1.01	0.32 – 3.03	0.994

*Significant ORs (p<0.05)

Associations when including potential contaminant blood cultures: When considering positive blood cultures for potential contaminants (**Supplemental Table 1**), an additional 45 infants had positive blood or CSF cultures (total of 86 infants with positive blood cultures). One infant had a contaminating blood culture, but grew unidentified gram-negative diplococci in the CSF, so this infants was originally classified as bacterial sepsis with a pathogen (total of 46 infants with potential blood culture contaminants). When including infants with positive blood cultures for both contaminants and pathogens, and infants with positive CSF cultures, culture positivity was not significantly associated with mortality (OR 1.74 [95% CI: 0.82 – 3.53]; p=0.135), or any DS (**Supplemental Table 3**). The

number of DS was also not significantly associated with bacterial sepsis when including all pathogens and potential contaminants (OR 1.09 [95% CI: 0.93 – 1.28]; p=0.273; **Supplemental Figure 3**).

Discussion

This study found that most WHO DS were significantly associated with mortality, but only one DS (i.e., not feeding well) as significantly associated with bacterial sepsis with a positive blood or CSF culture for a neonatal pathogen. Additionally, none of the other DS were significantly associated with bacterial sepsis in this LMIC neonatal cohort. The latter results held true whether only bacterial pathogens, or pathogens plus potential bacterial contaminants were considered. Notably, a significant minority (39%) of parent of infants with bacterial sepsis initially declared that their child was feeding well at presentation. Therefore, absence of this DS could practically miss a large proportion of infants with bacterial sepsis in real life. Few studies have attempted to determine how DS can predict bacterial sepsis caused by bacterial pathogens in young infants. A retrospective Brazilian study reviewed data on 83 sick neonates over a 12-month period, and came to the same conclusion that DS alone or in combination did not predict severe serious bacterial infection (15). The current study findings also support findings from another study carried out in primary care, rural setting in Bangladesh, India and Pakistan (16), clinical signs could not differentiate infants with viral or bacterial illnesses. To the best of our knowledge, however, the current study is the first to pragmatically examine whether DS predict bacterial sepsis in infants. These findings were obtained in an LMIC setting where DS were prospectively identified by front line healthcare workers as they would normally do in real-life, outside a research protocol – we assumed that this study design would more closely reflects the practical utility of DS. The current study findings have implications for a hospital setting. Essentially, it suggests that DS cannot reliably identify infants with bacterial sepsis who require antibiotic treatment. However, further research is needed to determine the generalizability of the results to other hospital contexts.

The strength of this study are its robust standardized blood culture outcome measures where contamination during blood sampling were minimized by using protocolized aseptic procedures, and the robust microbiological identification methods, including access to a certified clinical laboratory lab and protocols on-site augmented by advanced MALDI-TOF species determination in another, North American clinical microbiology laboratory. The strong association between bacterial sepsis and mortality provides reassurance infants with positive cultures were correctly identified.

In addition to the main findings, the current study also found that only a minor proportion of infants with suspected sepsis showed positive blood culture. A limited number of studies have prospectively estimated the incidence of bacterial sepsis in LMIC cohorts, and can be discussed here in the context of the current study findings. A hospital-centred study in Delhi, India, included 13,530 neonates (all comers; mean GA±SD: 36.0±3.4 weeks) monitored daily for clinical signs of sepsis with per-protocol completion of a sepsis work-up (blood culture and lumbar puncture) prior to initiating antibiotics, and found a 14.3% incidence of culture-positive sepsis (17), which is in keeping with the current study findings. Another study in community settings in Madagascar, Senegal and Cambodia reported 8.2% culture-confirmed sepsis among 514 neonates assessed for "possible serious bacterial infection" out of 3,688 infants followed during the neonatal period (18). One large study followed 12,622 live births up to 60 days of age in rural India between April 2002 and March 2005, with daily home visits and clinical assessment, and standardized blood and CSF cultures following enrollment of infants with suspected sepsis. It reported a 6.3% incidence of culture-confirmed sepsis (53 with positive blood cultures out of 842 infants assessed, excluding potential contaminants), for a population-level incidence of culture-confirmed sepsis of 6.7 per 1,000 births (19). Taken together, these findings support incidence rates

found in the current study, and provide robust data on the incidence of bacterial sepsis in LMIC settings, and form the basis for future studies to measure the impact of potential interventions.

Blood cultures are the gold standard for diagnosing bacterial sepsis in young infants (20). However, these tests lack sensitivity in neonates (21). In the current study we were careful to prescribe a minimum blood volume to maximize culture yield. Yet, a key question is whether we misestimate the proportion of bacterial sepsis in infants in whom cultures are negative? The Aetiology of Neonatal Infection in South Asia (ANISA) study (22) also attempted to identify the cause of sepsis for 6022 cases of possible serious bacterial infections in infants in the first 60 days after birth, in a population of 63,114 livebirth). Bacterial and viral (e.g., respiratory syncytial virus) causes were identified in 16% and 12% of cases, respectively. For the remaining 72% cases, no pathogen could be identified despite systematic blood and respiratory cultures, which is consistent with findings from the current study.

Evidently the use of antibiotics in the current study was high, with the vast majority of infants receiving antibiotics for a minimum of five days during hospitalization, as per hospital practices. As in other LMIC settings, blood cultures were not routinely available at the study site prior to the study. As such, no approach had been developed to stop antibiotics after 48 hours given a negative blood culture. In another study in Thailand, the duration of antibiotic use was also high with durations of 15 and 8 days for culture-positive and culture-negative early-onset neonatal sepsis, respectively (23). These results highlight the limits of having to rely on DS, and potential coercive effect of the lack of laboratory resources capable of providing basic laboratory test results to help "rule out" sepsis (24).

A number of study limitations should be acknowledged. Although a sizeable proportion of the recruited infants presented from home, data were collected in a single hospital-type setting; as such, caution

should be exercised when generalizing to other hospital settings or extrapolating to non-hospital primary care settings. For logistical reasons CSF was collected from only approximately half of the infants, which may have underestimated the incidence of bacterial sepsis. Similarly, urine or anaerobic blood cultures were not collected, which may have resulted in missed bacteremia. Some infants received antibiotics prior to blood culture, which may have lowered bacterial sepsis estimates. Moreover, due to the limited diagnostic capabilities of the study LMIC settings, in most cases, causes of deaths or alternate diagnoses could not be determined. Similarly, the study did not include non-bacterial testing, which precluded identification of viral sepsis syndromes.

In conclusion, this study validates the use of WHO DS to predict fatal outcomes, but not to reliably identify infants with bacterial sepsis. In other words, relying solely on DS to identify infants with bacterial sepsis is likely to lead to overtreatment while at the same time missing infants who require antibiotic treatment. This study further emphasizes the need for better diagnostics for bacterial sepsis, to ensure that health gains continue to improve, in areas of the world where sepsis represents a huge burden and where antimicrobial resistance is also increasing at alarming rates (25).

Acknowledgments

We thank Bentry Tembo, Rhoda Chifisi and Blessings Chiluzi for help with recruitment and data collection, Charles Mwansambo, Patience Johane and the staff at the PACHI for administrative and operational support during the study, Robert Krysiak, Gerald Tegha and the staff of the University of North Carolina – Malawi lab for performing all bacterial cultures and identification, on-site, in Lilongwe, according to clinical laboratory standards, Dr. David Goldfarb for expert input into data interpretation, Amber Johnson from the British Columbia Children’s Hospital Microbiology Laboratory for secondary confirming bacterial isolates by mass spectrometry.

REFERENCES

1. GBD 2016 Causes of Death Collaborators. Global, regional, and national age-sex specific mortality for 264 causes of death, 1980-2016: a systematic analysis for the Global Burden of Disease Study 2016. Lancet. 2017;390(10100):1151-210.
2. Rudd KE, Johnson SC, Agesa KM, Shackelford KA, Tsoi D, Kievlan DR, et al. Global, regional, and national sepsis incidence and mortality, 1990-2017: analysis for the Global Burden of Disease Study. Lancet. 2020;395(10219):200-11.
3. Fleischmann-Struzek C, Goldfarb DM, Schlattmann P, Schlapbach LJ, Reinhart K, Kissoon N. The global burden of paediatric and neonatal sepsis: a systematic review. The Lancet Respiratory medicine. 2018;6(3):223-30.
4. Costelloe C, Metcalfe C, Lovering A, Mant D, Hay AD. Effect of antibiotic prescribing in primary care on antimicrobial resistance in individual patients: systematic review and meta-analysis. BMJ. 2010;340:c2096.
5. Klingenberg C, Kornelisse RF, Buonocore G, Maier RF, Stocker M. Culture-Negative Early-Onset Neonatal Sepsis - At the Crossroad Between Efficient Sepsis Care and Antimicrobial Stewardship. Front Pediatr. 2018;6:285.
6. Costello A. Integrated management of childhood illness. Lancet. 1997;350(9087):1266.
7. Young Infants Clinical Signs Study Group. Clinical signs that predict severe illness in children under age 2 months: a multicentre study. The Lancet. 2008;371(9607):135-42.
8. Opiyo N, English M. What clinical signs best identify severe illness in young infants aged 0-59 days in developing countries? A systematic review. Archives of disease in childhood. 2011;96(11):1052-9.

9. World Health Organization. Pocket Book of Hospital Care for Children: Guidelines for the Management of Common Childhood Illnesses. WHO Guidelines Approved by the Guidelines Review Committee. 2nd ed. Geneva, 2013.
10. Wynn JL, Polin RA. Progress in the management of neonatal sepsis: the importance of a consensus definition. Pediatr Res. 2018;83(1-1):13-5.
11. Clinical prediction of serious bacterial infections in young infants in developing countries. The WHO Young Infants Study Group. Pediatr Infect Dis J. 1999;18(10 Suppl):S23-31.
12. Popescu CR, Tembo B, Chifisi R, Cavanagh MMM, Lee AH, Chiluzi B, et al. Whole blood genome-wide transcriptome profiling and metagenomics next-generation sequencing in young infants with suspected sepsis in a low-and middle-income country: A study protocol. Gates Open Res. 2020;4:139.
13. Popescu C, Tembo B, Chifisi R, Cavanagh M, Chiluzi B, Lufesi N, et al. Procedure policies, data collection form and model consent form (supplemental material)~Improving the Early Diagnosis of Neonatal Sepsis in Malawi. In: Scholars Portal Dataverse, editor. 2 ed 2020.
14. Downie L, Armiento R, Subhi R, Kelly J, Clifford V, Duke T. Community-acquired neonatal and infant sepsis in developing countries: efficacy of WHO's currently recommended antibiotics--systematic review and meta-analysis. Archives of disease in childhood. 2013;98(2):146-54.
15. Ronfani L, Vilarim JN, Dragovich D, Bacalhau AF, Cattaneo A. Signs of severe bacterial infection in neonates. J Trop Pediatr. 1999;45(1):48-51.
16. Arvay ML, Shang N, Qazi SA, Darmstadt GL, Islam MS, Roth DE, et al. Infectious aetiologies of neonatal illness in south Asia classified using WHO definitions: a primary analysis of the ANISA study. The Lancet Global health. 2022;10(9):e1289-e97.
17. Study IotDNI. Characterisation and antimicrobial resistance of sepsis pathogens in neonates born in tertiary care centres in Delhi, India: a cohort study. The Lancet Global health. 2016;4(10):e752-60.

18. Huynh BT, Kermorvant-Duchemin E, Chheang R, Randrianirina F, Seck A, Ratsima EH, et al. Severe bacterial neonatal infections in Madagascar, Senegal, and Cambodia: A multicentric community-based cohort study. Plos Medicine. 2021;18(9).
19. Panigrahi P, Chandel DS, Hansen NI, Sharma N, Kandefer S, Parida S, et al. Neonatal sepsis in rural India: timing, microbiology and antibiotic resistance in a population-based prospective study in the community setting. J Perinatol. 2017;37(8):911-21.
20. Polin RA. Management of neonates with suspected or proven early-onset bacterial sepsis. Pediatrics. 2012;129(5):1006-15.
21. Popescu CR, Cavanagh MMM, Tembo B, Chiume M, Lufesi N, Goldfarb DM, et al. Neonatal sepsis in low-income countries: epidemiology, diagnosis and prevention. Expert Rev Anti Infect Ther. 2020;18(5):443-52.
22. Saha SK, Schrag SJ, El Arifeen S, Mullany LC, Shahidul Islam M, Shang N, et al. Causes and incidence of community-acquired serious infections among young children in south Asia (ANISA): an observational cohort study. Lancet. 2018;392(10142):145-59.
23. Kiatchoosakun P, Jirapradittha J, Sirikarn P, Laopaiboon M, Pattanittum P, Chandrakachorn W, et al. Early-Onset Neonatal Sepsis and Antibiotic Use in Northeast Thailand. Am J Perinatol. 2019;36(12):1295-303.
24. Lavoie PM, Popescu CR, Molyneux EM, Wynn JL, Chiume M, Keitel K, et al. Rethinking management of neonates at risk of sepsis. Lancet. 2019;394(10195):279-81.
25. Iregbu K, Dramowski A, Milton R, Nsutebu E, Howie SRC, Chakraborty M, et al. Global health systems' data science approach for precision diagnosis of sepsis in early life. Lancet Infect Dis. 2022;22(5):e143-e52.

Click here to access/download
Supporting Information
WHO Manuscript - [Supplemental Data] - Final.docx

Click here to access/download
Supporting Information
REB Approval - PGPH-D-23-00809.pdf

Click here to access/download
Supporting Information
STROBE-checklist WHO DS Manuscript.docx

Click here to access/download
Supporting Information
Inclusivity-in-global-research-questionnaire.docx

Complete dataset

Click here to access/download
Supporting Information
WHO DS - Final DataSet - Jul 11 2023.xlsx

World Health Organization Danger Signs to predict bacterial sepsis in young infants: A pragmatic cohort study

Omolabake Akinseye, MBBS[1,#], Constantin R. Popescu, MD, MSc[2,3,#], Msandeni Chiume-Kayuni MMED Peads, MSc[4,5], Michael A. Irvine, PhD[6,7], Norman Lufesi DCM, MPhil[8], Tisungane Mvalo, MBBS, MMED[9,10], Niranjan Kissoon, MD, MCCM[1,2], Matthew O. Wiens, PharmD, PhD[11], Pascal M. Lavoie, MDCM, PhD[1,2,*]

[#] contributed equally as co-first authors

Affiliations:
[1] Department of Pediatrics, Faculty of Medicine, University of British Columbia, Vancouver, Canada
[2] British Columbia Children's Hospital Research Institute, Vancouver, Canada
[3] Department of Pediatrics, Université Laval, Québec, Canada
[4] Department of Pediatrics, Kamuzu Central Hospital, Lilongwe, Malawi
[5] Kamuzu University of Health Sciences, Lilongwe, Malawi
[6] British Columbia Centre for Disease Control, Vancouver, Canada
[7] Faculty of Health Sciences, Simon Fraser University, Burnaby, Canada
[8] Department of Curative and Medical Rehabilitation, Ministry of Health, Lilongwe, Malawi
[9] University of North Carolina Project Malawi, Lilongwe, Malawi
[10] Department of Pediatrics, University of North Carolina at Chapel Hill School of Medicine, Chapel Hill, NC, USA
[11] Department of Anesthesiology, Pharmacology & Therapeutics, Faculty of Medicine, University of British Columbia, Vancouver, Canada

***Corresponding author:** Dr. Pascal M. Lavoie
BC Children's Hospital Research Institute
4th Floor, Translational Research Building
950 West 28th Avenue
Vancouver, British Columbia V5Z 4H4, Canada
Email: plavoie@bcchr.ca
Fax: +1-604-875-3106

Short title: Prediction of bacterial sepsis in infants.

Conflict of Interest Statement: The authors declare no conflicts of interest.

Funding Support: This study was support by a grant from Grand Challenges Canada (R-ST-POC-1707-04630). PML was supported by the BC Children's Hospital Research Foundation through the Investigator Grant Award Program. CRP was funded by a Sue Carruthers Graduate Studentship from the BC Children's Hospital Foundation. The other authors received no additional funding.

Role of Funder/Sponsor: The funder/sponsor did not participate in the work.

Abbreviations: WHO: World Health Organization; DS: danger signs; LMICs: low and middle-income countries; CSF: cerebrospinal fluid; AUC-ROC: Area Under Curve – Receiver Operator Characteristics; bpm: breaths per minute.

Abstract

Background: Bacterial sepsis is generally a major concern in ill infants. To help triaging decisions by front-line health workers in these situations, the World Health Organization (WHO) has developed danger signs (DS). The objective was to evaluate the extent to which nine DS predict bacterial sepsis in young infants presenting with suspected sepsis in a low-income country setting.

Methodology: This study pragmatically evaluated nine DS in infants younger than 3 months with suspected sepsis in a regional hospital in Lilongwe, Malawi, between June 2018 and April 2020. Main outcomes were positive blood or cerebrospinal fluid (CSF) cultures for neonatal pathogens, and mortality.

Findings/Results: Among 401 infants (gestational age [mean ± SD]: 37.1±3.3 weeks, birth weight 2865±785 grams), 41 had positive blood or CSF cultures for a neonatal pathogen. In-hospital mortality occurred in 9.7% (N=39/401) of infants overall (N=39/401), of which 61.5% (24/39) occurred within 48 hours of admission. Mortality was higher in infants with bacterial sepsis compared to other infants (22.0% [9/41] versus 8.3% [30/360]; p=0.005). All DS were associated with mortality except for temperature instability and tachypnea, whereas none of the DS were significantly associated with bacterial sepsis, except for "unable to feed" (OR 2.25; 95%CI: 1.17 – 4.44; p=0.017). The number of DS predicted mortality (OR: 1.75; 95%CI: 1.43 – 2.17; p<0.001; AUC: 0.756), but was marginally associated with positive cultures with a neonatal pathogen (OR 1.22; 95%CI: 1.00 – 1.49; p=0.046; AUC: 0.743). The association between number of DS and mortality remained significant after adjusting for admission weight, the only statistically significant co-variable (OR 1.75 [95% CI: 1.39 – 2.23]; p<0.001). Considering all positive cultures including potential bacterial contaminants resulted a non-significant association between number of DS and sepsis (OR 1.09 [95% CI: 0.93 – 1.28]; p=0.273).

Conclusion/Significance: DS predicted death, but was marginally associated with culture-positive pathogen sepsis in a regional hospital setting. These data imply that the incidence of bacterial sepsis and attributable mortality in infants based on clinical signs may not be accurate, in LMIC settings.

Introduction

Three million children die each year worldwide in their first month of age, with a significant proportion of these deaths occurring during the first week(1). Severe infections (including sepsis, pneumonia, diarrhea and viral diseases) are common causes of serious illnesses in young infants (2). Up to 25% of cases in low- and middle-income countries (LMICs) have identified bacterial causes (3). However, the lack of diagnostic resources, particularly in LMICs makes it difficult to accurately quantify this burden. This gap has enormous implications in terms of implementing policies and guidelines for the use of antimicrobials to prevent these deaths (1). At the individual level, identifying bacterial causes for sepsis is crucial to prevent fatalities and guide the correct empiric antibiotic therapy in the context of rising antimicrobial resistance. Indeed, the misuse, or even the overuse, of antibiotics carries a risk of subsequent treatment failure due to colonization by resistant bacteria (4, 5).

To assist front-line health workers, the World Health Organization (WHO) and United Nations International Children's Emergency Fund developed algorithms in the early 1990s to help triage interventions for sick children (6). This Integrated Management of Childhood Illness (IMCI) strategy included the identification of clinical signs to alert the need for escalation of care, later termed Danger Signs (DS) (6). Subsequently, the Young Infant Clinical Signs Study Group carried out a multi-centered study in six LMICs and found that seven signs (i.e., history of difficulty feeding, grunting, convulsions, movement only when stimulated, respiratory rate ≥60 breaths per minute (bpm), severe chest indrawing, temperature below 35.5°C or above 37.5°C, prolonged capillary refill, cyanosis and stiff limbs) best predicted the need for higher level of care, with a sensitivity of 74% to 85%, and specificity of 75% to 79%, depending on infants' age (i.e., 0 to 6 days versus 7 to 59 days old) (7). A systematic review of five studies on 17,506 infants found similar results (8).

Over time, the use of DS has been extended to provide a reasonable basis for the initiation of empiric antibiotic treatment (9). However, these signs have neither been developed nor validated to diagnose sepsis of bacterial etiology. Neonatal disorders such as transient tachypnea of the newborn, hypoglycemia, hyaline membrane disease, birth asphyxia, and viral infections (to mention only a few), present like bacterial sepsis (10). Hence, a key question is whether DS can reasonably identify young infants who truly benefit from antibiotic treatment. Only a few studies have attempted to answer this question, but certainly none that have, to the best of our knowledge, been conducted prospectively, in a pragmatic setting where clinicians, not research staff, pragmatically reported DS (11).

The objective of this study was to determine the extent to which WHO DS predict bacterial sepsis in young infants, in a LMIC hospital setting where front-line health workers were asked to assess and record the presence of DS in infants who presented with suspected sepsis.

Materials and Methods

Study approval: The study was approved by the National Health Science Research Committee of Malawi (#17/8/1819) and the University of British Columbia's Children's and Women's Research Ethics Board (#H16-02639). The study was ~~discussed and~~ supported by the chair of the Department of Pediatrics at Kamuzu Central Hospital and discussed with ~~,~~ leaders from the Pediatric and Child Health Association (PACHA), ~~as well as~~ UNICEF Malawi and the ~~Health Minister~~Ministry of Health of Malawi.

Study design, setting & population: This study was a pragmatic cohort study, and secondary analysis of data from a parent study that aimed to identify molecular RNA-sequencing signatures of bacterial sepsis in young infants with suspected sepsis~~, defined according to the attending staff's suspicion~~ (12).

In the parent study, infants below 3 months of age with suspected sepsis, defined according to the attending staff's clinically suspicion, were sequentially enrolled "around the clock" at the time they presented to the neonatal or pediatric units at Kamuzu Central Hospital in Lilongwe, Malawi between June 5th, 2018, and April 6th, 2020 (including recruitment, exposure~~, follow-up~~ and data collection). To minimize bias towards false-negative blood culture, infants were excluded if they had ~~were already~~been on antibiotics more than~~for >~~ 4 hours prior to the cultures~~at the time of enrollment~~. About 851 infants presented with suspected sepsis and the vast majority were not enrolled because staff were unable to get consent – either parents declined, or were not approached for consent due to lack of time and/or competing clinical demands. Written informed consent was obtained from parents/guardian of each participant, in English or Chichewa (main local language). Enrolled infants were followed during ~~their entire~~ hospitalization at Kamuzu Central Hospital, until transferred to another facility or discharged home.

The Kamuzu Central Hospital the largest referral hospital in central Malawi, where approximatively 3,000 infants are admitted to its neonatal unit annually. "Suspected sepsis" was defined as per the clinical staff, essentially in an infant that looked unwell where bacterial sepsis could not be excluded clinically. Before the study, the staff at Kamuzu Central Hospital did not have routinely access to blood culture or other ancillary tests, IV fluids and gavage feeds were also most often not available due to limited resources and supplies. Available interventions include antibiotics, assistance with feeding and routine surgeries when necessary. Oxygen and continuous positive pressure ventilation were only available to an extremely limited number of infants, on a limited basis when the hospital had electrical power. Invasive mechanical ventilation was not possible. During the study, investigators did not have access to information that could identify participants, ~~with the exception of~~except for the consent forms which were kept under the responsibility of the local study PI (MCK). The study was administered and

local research staff were hired through a partnership with the Parent and Child Health Initiative (PACHI), a Malawian non-profit non-governmental organization, which works in collaboration with the Ministry of Health.

Clinical data and blood samples collection: Each infant had an initial clinical evaluation by a healthcare worker (or staff, either a nurse or clinical officer). The presence of the following nine DS were prospectively collected at presentation, using electronic forms, on a tablet device: i) history of not feeding well (interviewing the parent/caregiver), entered as “feeding well”, “unable to feed” or “uninterested in feeding” (combining the latter two categories), ii) fast breathing ≥60 bpm (with respiratory rate visually counted by attended clinician), iii) temperature instability, defined as axillary body temperature <35.5°C or >38°C, (measured using an electronic Welch Allyn SureTemp thermometer, model 692), iv) drowsiness/difficulty to wake, v) grunting, vi) severe chest recession, vii) central cyanosis, viii) movements only when stimulated or no movement at all and ix) convulsions (defined pragmatically by attending clinician). Additionally, clinical staff had to report whether they felt the infant was “well”, “ill” or “severely ill” at the time of assessment. However, i~~I~~n keeping with a pragmatic design, the study did not attempt to enforce strict criteria ~~to define suspected sepsis, DS or~~ for illness status, or for to define suspected sepsis or the presence or absence of DS. All infants also had a standardized aerobic blood culture (minimum 2 mL of blood) collected within 4 hours of presentation, using a rigorously implemented aseptic protocol (with in-training of all front-line healthcare providers prior to study initiation), as detailed (13). ~~Infants also had c~~Cerebrospinal fluid (CSF) ~~and urine~~ cultures were collected by clinical staff, whenever they felt it was clinically indicated, at their discretion~~ of the clinical staff~~, based on availability of supplies, ~~of resources~~ and the cost of the test that had to be paid by parents. No urine cultures were performed.

Blood cultures, were were paid by the study, and processed into BACTEC PEDS Plus culture bottles incubated for 5 days in a BD BACTEC 9050 instrument. In keeping with a pragmatic design, the study did not attempt to enforce strict criteria to define suspected sepsis, DS or illness status. Identification of organisms from blood or CSF cultures was completed in-house using bioMerieux API, and BD Crystal kits at a local University of North Carolina-affiliated research facility, as described (12). Bacterial isolates that could be restored (~60% of isolates) were also analyzed for further confirmation of identity on a Microflex LT MALDI-TOF mass spectrometer, using the BioTyper software version 3.1 (Bruker Daltonik, Germany). Culture results were made available to clinicians involved in the care of the infants.

Outcome measures: The primary outcomes of interest were *mortality*, and *culture-positive sepsis* defined as the bacterial growth of a known neonatal pathogen in blood or CSF culture sample. Micro-organisms not commonly considered as pathogens in term or near-term infants, such as *coagulase-negative Staphylococcus*, *Micrococcus*, *Corynebacterium, Bacillus species*, or streptococcus species such as *S. oralis* or *Bacillus* were considered as potential contaminants and were excluded from the main bacterial sepsis outcome (14), but , but these potential contaminants were included in a sensitivity analysis using an alternate bacterial sepsis outcome “including potential contaminants” (**Supplemental Table 1**). In-hospital mortality was recorded until discharge home or transfer to another hospital facility. Data were considered missing when infants were transferred within 72 hours after initial admission to the hospital.

Statistical Analysis: In the parent study, the sample size was set on the original molecular signature aim, conveniently at 500 infants over two years, assuming a 20% incidence of bacterial sepsis and expecting to identify between 10 and 50 differentially expressed molecular gene markers (12).

Formatted: Font: Italic

Formatted: Font: Italic

Formatted: Font: Italic

Formatted: Font: Italic

Formatted: Font: Italic

However, but the study needed to be stopped early, in April 2020 due to the COVID-19 pandemic, so in the end only 401 infants were enrolled. All infants originally enrolled in the parent study were analyzed. Demographic characteristics were reported descriptively using the mean/median with dispersion. The nine aforementioned WHO DS were used as predictors. All quantitative variables were analyzed as such, except for fast breathing and temperature instability which were categorized according to WHO definitions (see above). Odds ratios were calculated for each DS and the main outcomes of bacterial sepsis or mortality. Logistic regressions were conducted between the number of DS and the main outcomes. In separate analyses, odds ratios were calculated using logistic regressions between mean DS (i.e., the number of DS divided by the available DS data) to determine the effect of missing DS data. Unadjusted logistic regression models, including demographic variables, were conducted to determine the predictive value of the DS. The predictive performance of DS for each outcome was assessed using the area under receiver operator characteristic curve (AUC-ROC). Sociodemographic variables associated with mortality, as well as their independent effects were analyzed using regression models. Only complete cases were used in the analysis for associations with individual DS, or the outcome "cumulative number of DS". However, analyses were also conducted using "mean number of DS", to account for missing values, with no significant differences in results. Statistical significance was considered at p-value <0.05 with no adjustment made for multiple testing in unadjusted models. Analyses were performed using R (version 4.1.0) and SPSS version 20 (IBM, Armonk, NY, USA).

Inclusivity in global research: Additional information regarding the ethical, cultural, and scientific considerations specific to inclusivity in global research is included in the **Supporting Information**.

Results

Study population: The majority of the 401 infants included in the study were term or late preterm infants, born vaginally, presented from home in their first week of post-natal life, and had roughly equal female/male distribution (**Table 1**). Most infants were reported by clinicians to look ill at the time of initial presentation (69.3%; 278/401), 22.2% (89/401) were severely ill, and 8.5% appeared well (34/401). In total, 79.8% (320/401) presented at least one DS. Overall, 56.9% (228/401) of infants had been ill for at least 24 hours prior to initial presentation, and their parents had traveled a median distance of 17 kilometers, often by foot or taxi, to reach the nearest hospital. In total, 94/401 (23.4%) infants had received antibiotics prior to the blood culture, for a median of 2 hours (range 0.75 to 3.5 hours). The duration of antibiotic use in infants ranged from 1 to 30 days (**Table 1**). Few infants with negative blood culture had antibiotics discontinued after 48 hours (21/401, 5.2%).

Table 1: Socio-demographic characteristics of the study population.

**Participant characteristics**		**N with data**
Gestational age at birth (weeks), mean (SD)	37.1 (3.3)	397
Birth weight (gram), mean (SD)	2865 (785)	380
Female, no (%)	185 (46)	401
Admitted from, no (%)		401
Inborn (i.e., admitted from birth hospital)	161 (40.1)	
Transferred from another facility	224 (55.8~~6~~)	
Home	16 (4.0)	
Post-natal age at presentation (days), median (IQR)	4 (2-16)	401
Mode of delivery, no (%)		398

Caesarean section	101 (25.2)	
Vaginal delivery	297 (71.8)	
Duration of illness before presentation to hospital (hours), median (IQR)	24 (12-48)	341
Distance of travel to hospital (km), median (IQR)	17 (11-25)	342
Illness status, number on infants (%)		401
Well	34 (8.5)	
Ill	278 (69.3)	
Severely ill	89 (22.2)	
Total d~~D~~uration of antibiotics (days), median (IQR)	6 (5-9)	401
Duration of hospital stay (days), median (IQR)	7 (5-9)	401
Danger Signs, number of infants (%)		
Not feeding well	171 (43.3)	395
Convulsions	28 (7.0)	401
Drowsy / Difficult to wake	33 (8.2)	401
Movements only when stimulated or no movements	26 (6.5)	401
Fast breathing >=60 per min	104 (27.1)	384
Grunting	30 (7.5)	401
Severe chest recessions	51 (12.7)	401
Temperature instability >38°C or <35.5°C	118 (32.7)	361
Central cyanosis	29 (7.2)	401

Outcome data: The proportion of infants with bacterial sepsis, considering only known pathogens in term and near-term infants, was 10.2% (41/401). Blood (39/401) and CSF (2/204) cultures were positive for pathogens in 9.7% and 1.0% of the infants, respectively. ~~One infant had a contaminating blood culture, but grew unidentified gram-negative diplococci in the CSF, so was classified as bacterial sepsis with a pathogen.~~ Of 230 infants under 7 days old, 21 (9.1%) had bacterial sepsis with a pathogen versus 20 of the 171 infants (11.7%; p=0.402 by chi-square test) ≥ 7 days old. The proportion of gram-positive sepsis (21/39) and gram-negative sepsis (20/39) were similar among all infants (**Supplemental Table 1**), and were also similar between infants under 7 days (9 versus 12) and infants ≥ 7 days old (12 versus 8; p=0.272 by chi-square test, noting however the small sample size for comparison). The proportion of sepsis was also similar in inborn infants (19/161) versus infants transferred from another hospital facility (22/224) or admitted from home (0/16). Three infants were transferred before 72 hours from admission, so the mortality outcome was considered missing in these infants. Among the remaining infants, mortality was 9.7% (i.e., 39/398), of which 61.5% [24/39] died within 48 hours.

Associations with mortality and culture-positive outcomes: The following DS were significantly associated with mortality (**Table 2**): *not feeding well*, *convulsions*, *drowsy/difficult to wake*, *movements only when stimulated or no movements*, *grunting*, *severe chest recessions*, *central cyanosis*. In contrast, *fast breathing* or *temperature instability* were not significantly associated with mortality. The number of DS was also significantly associated with mortality (OR 1.75 [95%CI: 1.43 – 2.17] per cumulative DS; p<0.001; AUC: 0.743) (**Supplemental Figure 1**). Admission weight was significantly associated with mortality, but neither age of the mother, gestational age, birth weight nor sex were significantly associated with mortality (**Supplemental Table 2**). When adjusting for these co-variables, both the

Formatted: Space After: 0 pt, Line spacing: Double

cumulative (OR 1.75 [95% CI: 1.39 – 2.23]; p<0.001) and mean number of DS (1.80 [95% CI: 1.44 – 2.30]; p<0.001) remained highly significantly associated with mortality (**Supplemental Table 2**).

Formatted: Space After: 0 pt, Line spacing: Double

Table 2: Associations between DS and mortality.

Variable	Odds ratio (OR)	95%CI	P value
Not feeding well	7.21	3.27 – 18.2	<0.001*
Convulsions	3.52	1.31 – 8.59	0.008*
Drowsy / Difficult to wake	5.04	2.12 – 11.4	<0.001*
Movements only when stimulated or no movements	4.89	1.88 – 11.9	<0.001*
Fast breathing >=60 per min	1.86	0.87 – 3.85	0.100
Grunting	3.95	1.54 – 9.34	0.002*
Severe chest recessions	3.01	1.34 – 6.37	0.005*
Temperature instability >38°C or <35.5°C	1.70	0.75 – 3.75	0.191
Central cyanosis	4.15	1.62 – 9.87	0.002*

*Significant ORs (p<0.05)

Mortality was higher in infants with bacterial sepsis with a known neonatal pathogen compared to other infants (22.0% [9/41] versus 8.3% [30/360]; p=0.005). Bacterial sepsis ~~with a known neonatal pathogen~~ was significantly associated with mortality (OR 3.07 [95%CI: 1.28 – 6.82]; p = 0.008). However, the only DS significantly associated with bacterial sepsis when considering only known neonatal pathogens was *not feeding well* (OR 2.25 [95%CI: 1.17 – 4.44]; p=0.017 (**Table 3**); ~~p=0.017~~61.0% [25/41] of the children with bacterial sepsis were not feeding well versus 41.2% [146/354] of the other children) ~~(**Table 3**)~~. Associations between convulsions, drowsiness, lethargy (i.e., movements only when stimulated or no movement), tachypnea, grunting, severe chest recession, temperature instability and central cyanosis were all non-significant (**Table 3**). Association was between cumulative number of DS and ~~pathogen~~ bacterial sepsis was marginally significant (OR 1.22

Formatted: Font: Bold

Formatted Table

[95% CI: 1.00 – 1.49]; p=0.046) (**Supplemental Figure 2**). At discharge, clinical staff selected non-bacterial sepsis in 76.3% (306/401), bacterial sepsis in 15.5% (62/401) and “other non-sepsis syndromes” in 8.2% (33/401) of cases, from these options, for study purposes.

Table 3: Association between DS and bacterial sepsis when organism is a known pathogen.

**Variable**	**Odds ratio (OR)**	**95%CI**	**P value**
Not feeding well	2.25	1.17 – 4.44	0.017*
Convulsions	0.65	0.10 – 2.30	0.571
Drowsy / Difficult to wake	0.86	0.20 – 2.57	0.811
Movements only when stimulated or no movements	1.65	0.46 – 4.59	0.382
Fast breathing >=60 per min	1.06	0.49 – 2.17	0.868
Grunting	2.38	0.84 – 5.89	0.077
Severe chest recessions	2.17	0.92 – 4.74	0.061
Temperature instability >38°C or <35.5°C	1.60	0.78 – 3.24	0.193
Central cyanosis	1.01	0.32 – 3.03	0.994

*Significant ORs (p<0.05)

Associations when including potential contaminant blood cultures: When considering positive blood cultures for potential ~~blood culture~~ contaminants ~~(e.g., *micrococcus, Corynebacterium, bacillus* and *coagulase-negative staphylococcus* species and non-pathogenic *streptococcus* species such as *S. oralis*)~~(**Supplemental Table 1**), an additional 45 infants had positi~~86 infants had positi~~ve blood or CSF cultures (total of 86 infants with positive blood cultures). One infant had a contaminating blood culture, but grew unidentified gram-negative diplococci in the CSF, so this infants was originally classified as

Formatted: Font: Bold

bacterial sepsis with a pathogen (total of 46 infants with potential blood culture contaminants). When including infants with positive blood cultures for both contaminants and pathogens, and infants with positive CSF cultures, ~~Bacterial~~ culture positivity was not significantly associated with mortality (OR 1.74 [95% CI: 0.82 – 3.53]; p=0.135), or any DS ~~when including contaminants~~ (**Supplemental Table 3**). The number of DS was also not significantly associated with bacterial sepsis when including all pathogens and potential contaminants (OR 1.09 [95% CI: 0.93 – 1.28]; p=0.273; **Supplemental Figure 3**).

Discussion

This study found that most WHO DS were significantly associated with mortality, but only one DS (i.e., not feeding well) as significantly associated with ~~culture-proven~~ bacterial sepsis with a positive blood or CSF culture for a neonatal pathogen. Additionally, ~~the majority of~~none of the other DS were ~~not~~significantly associated with ~~culture-proven~~ bacterial sepsis in this LMIC neonatal cohort. The latter results held true whether only bacterial pathogens, or pathogens plus potential bacterial contaminants were considered. Notably, a significant minority (39%) of parent of infants with bacterial sepsis initially declared that their child was feeding well at presentation. Therefore, absence of this DS could practically miss a large proportion of infants with bacterial sepsis in real life. ~~To the best of our knowledge, the current study is the first to pragmatically examine whether DS predict bacterial sepsis in infants. These findings were obtained in an LMIC setting where DS were prospectively identified by front line healthcare workers as they would normally do pragmatically outside a research protocol – we assumed that this study design would more closely reflects the real-life utility of DS.~~ Few studies have attempted to determine how DS can predict bacterial sepsis caused by bacterial pathogens in ~~-~~young infants. A retrospective Brazilian study reviewed data on 83 sick neonates over a 12-month period, and

came to the same conclusion that DS alone or in combination did not predict severe serious bacterial infection (15). The ~~findings of the~~ current study findings also ~~are~~ support findings from ~~also consistent with those of~~ another study carried out in ~~a~~ primary care, rural setting in Bangladesh, India and Pakistan (16)~~. In the latter study~~, clinical signs could not differentiate infants with viral or bacterial illnesses ~~(16)~~. To the best of our knowledge, however, the current study is the first to pragmatically examine whether DS predict bacterial sepsis in infants. These findings were obtained in an LMIC setting where DS were prospectively identified by front line healthcare workers as they would normally do ~~pragmatically~~in real-life, outside a research protocol – we assumed that this study design would more closely reflects the ~~real-life~~practical utility of DS. The current study findings have implications for a hospital setting. Essentially, it suggests that DS cannot reliably identify infants with bacterial sepsis who require antibiotic treatment. However~~, but~~, further research is needed to determine the generalizability of the results to other hospital contexts.

The ~~A~~ strength of this study ~~is~~are its robust ~~outcomes, with~~ standardized blood culture ~~procedures~~outcome measures where ~~to ensure that the chances of~~ contamination during blood sampling were minimized by using protocolized aseptic procedures, and the robust microbiological identification methods, including access to a certified clinical laboratory lab and protocols on-site augmented by advanced MALDI-TOF species determination in another, North American clinical microbiology laboratory. The strong association between bacterial sepsis and mortality provides reassurance infants with positive cultures were correctly identified.

In addition to the main findings, the current study also found that only a minor proportion of infants with suspected sepsis showed positive blood culture. ~~Only~~ A limited number of~~few~~ ~~other~~ studies have

prospectively estimated the incidence of bacterial sepsis in LMIC cohorts, and can be discussed here in the context of the current study findings~~, and it is therefore useful to present the current findings in the context of those other studies~~. A hospital~~-~~ centred study in Delhi, India, included 13,530 neonates (all comers; mean GA±SD: 36.0±3.4 weeks) monitored daily for clinical signs of sepsis with per-protocol completion of a sepsis work-up (blood culture and lumbar puncture) prior to initiating antibiotics, and found a 14.3% incidence of culture-positive sepsis (17), which is in keeping ~~similar to~~with the current study findings~~ of the current study~~. Another study in community settings in Madagascar, Senegal and Cambodia reported 8.2% culture-confirmed sepsis among 514 neonates ~~infants~~ assessed for "possible serious bacterial infection" out of 3,688 ~~neonates~~ infants followed during the neonatal period (18). One large study followed 12,622 live births up to 60 days of age in rural India between April 2002 and March 2005, with daily home visits and clinical assessment, and standardized blood and CSF cultures following enrollment of infants with suspected sepsis. It reported a 6.3% incidence of culture-confirmed sepsis (53 with positive blood cultures out of 842 infants assessed, excluding potential contaminants), for a population-level incidence of culture-confirmed sepsis of 6.7 per 1,000 births (19). Taken together, these findings support incidence rates found in the current study, and provide robust data on the incidence of bacterial sepsis in LMIC settings,~~, to inform interventions~~ and form the basis for future studies to measure the impact of ~~those~~ potential interventions.

Blood cultures are the gold standard for diagnosing bacterial sepsis in young infants (20). However, these tests lack sensitivity in neonates (21). In the current study we were careful to prescribe a minimum blood volume to maximize culture yield. Yet, a key question is whether we misestimate the proportion of bacterial sepsis in infants in whom cultures are negative? The Aetiology of Neonatal Infection in South Asia (ANISA) study (22) also attempted to identify the cause of sepsis for 6022

cases of possible serious bacterial infections in infants in the first 60 days after birth, in a population of 63,114 livebirth). Bacterial and viral (e.g., respiratory syncytial virus) causes were identified in 16% and 12% of cases, respectively. For the remaining 72% cases, no pathogen could be identified despite systematic blood and respiratory cultures, which is consistent with findings from the current study.

Evidently the use of antibiotics in the current study was high, with the vast majority of infants receiving antibiotics for a minimum of five days during hospitalization, as per hospital practices. As in other LMIC settings, blood cultures were not routinely available at the study site prior to the study. As such, no approach had been developed to stop antibiotics after 48 hours given a negative blood culture. In another study in Thailand, the duration of antibiotic use was also high with durations of 15 and 8 days for culture-~~proven~~ positive and culture-negative early-onset neonatal sepsis, respectively (23). These results highlight the limits of having to rely on DS, and potential coercive effect of the lack of laboratory resources capable of providing basic laboratory test results to help "rule out" sepsis (24).

~~A strength of this study is its robust outcomes, with standardized blood culture procedures to ensure that the chances of contamination during blood sampling were minimized, and robust microbiological identification methods including clinical laboratory protocols on-site augmented by advanced MALDI-TOF species determination in another, North American clinical microbiology laboratory. The strong association between bacterial sepsis and mortality provides reassurance infants with positive cultures were correctly identified.~~ A number ~~However, a number~~ of study limitations should be acknowledged. Although a sizeable proportion of the recruited infants presented from home, data were collected in a single hospital-type setting; as such, caution should be exercised when generalizing to other hospital settings or extrapolating to non-hospital primary care settings. For logistical reasons CSF was collected

from only approximately half of the infants, which may have underestimated the incidence of bacterial sepsis. Similarly, urine or anaerobic blood cultures were not collected, which may have resulted in missed bacteremia. Some infants received antibiotics prior to blood culture, which may have lowered bacterial sepsis estimates. Moreover, due to the ~~extremely~~ limited diagnostic capabilities of the study LMIC settings, in most cases, causes of deaths or alternate diagnoses could not be determined. Similarly, the study did not include non-bacterial testing, which precluded identification of viral sepsis syndromes.

In conclusion, ~~our~~ this study validates the use of WHO DS to predict fatal outcomes, but not to reliably identify infants with ~~not~~ bacterial sepsis~~, in young infants~~. In other words, relying solely on DS to identify infants with bacterial sepsis is likely to lead to overtreatment while at the same time missing infants who require antibiotic treatment. This study further emphasizes the need for better diagnostics for bacterial sepsis, to ensure that health gains continue to improve, in areas of the world where sepsis represents a huge burden and where antimicrobial resistance is also increasing at alarming rates (25).

Acknowledgments

We thank Bentry Tembo, Rhoda Chifisi and Blessings Chiluzi for help with recruitment and data collection, Charles Mwansambo, Patience Johane and the staff at the PACHI for administrative and operational support during the study, Robert Krysiak, Gerald Tegha and the staff of the University of North Carolina – Malawi lab for performing all bacterial cultures and identification, on-site, in Lilongwe, according to clinical laboratory standards, Dr. David Goldfarb for expert input into data interpretation, Amber Johnson from the British Columbia Children’s Hospital Microbiology Laboratory for secondary confirming bacterial isolates by mass spectrometry.

REFERENCES

1. GBD 2016 Causes of Death Collaborators. Global, regional, and national age-sex specific mortality for 264 causes of death, 1980-2016: a systematic analysis for the Global Burden of Disease Study 2016. Lancet. 2017;390(10100):1151-210.
2. Rudd KE, Johnson SC, Agesa KM, Shackelford KA, Tsoi D, Kievlan DR, et al. Global, regional, and national sepsis incidence and mortality, 1990-2017: analysis for the Global Burden of Disease Study. Lancet. 2020;395(10219):200-11.
3. Fleischmann-Struzek C, Goldfarb DM, Schlattmann P, Schlapbach LJ, Reinhart K, Kissoon N. The global burden of paediatric and neonatal sepsis: a systematic review. The Lancet Respiratory medicine. 2018;6(3):223-30.
4. Costelloe C, Metcalfe C, Lovering A, Mant D, Hay AD. Effect of antibiotic prescribing in primary care on antimicrobial resistance in individual patients: systematic review and meta-analysis. BMJ. 2010;340:c2096.
5. Klingenberg C, Kornelisse RF, Buonocore G, Maier RF, Stocker M. Culture-Negative Early-Onset Neonatal Sepsis - At the Crossroad Between Efficient Sepsis Care and Antimicrobial Stewardship. Front Pediatr. 2018;6:285.
6. Costello A. Integrated management of childhood illness. Lancet. 1997;350(9087):1266.
7. Young Infants Clinical Signs Study Group. Clinical signs that predict severe illness in children under age 2 months: a multicentre study. The Lancet. 2008;371(9607):135-42.
8. Opiyo N, English M. What clinical signs best identify severe illness in young infants aged 0-59 days in developing countries? A systematic review. Archives of disease in childhood. 2011;96(11):1052-9.

9. World Health Organization. Pocket Book of Hospital Care for Children: Guidelines for the Management of Common Childhood Illnesses. WHO Guidelines Approved by the Guidelines Review Committee. 2nd ed. Geneva, 2013.
10. Wynn JL, Polin RA. Progress in the management of neonatal sepsis: the importance of a consensus definition. Pediatr Res. 2018;83(1-1):13-5.
11. Clinical prediction of serious bacterial infections in young infants in developing countries. The WHO Young Infants Study Group. Pediatr Infect Dis J. 1999;18(10 Suppl):S23-31.
12. Popescu CR, Tembo B, Chifisi R, Cavanagh MMM, Lee AH, Chiluzi B, et al. Whole blood genome-wide transcriptome profiling and metagenomics next-generation sequencing in young infants with suspected sepsis in a low-and middle-income country: A study protocol. Gates Open Res. 2020;4:139.
13. Popescu C, Tembo B, Chifisi R, Cavanagh M, Chiluzi B, Lufesi N, et al. Procedure policies, data collection form and model consent form (supplemental material)~Improving the Early Diagnosis of Neonatal Sepsis in Malawi. In: Scholars Portal Dataverse, editor. 2 ed 2020.
14. Downie L, Armiento R, Subhi R, Kelly J, Clifford V, Duke T. Community-acquired neonatal and infant sepsis in developing countries: efficacy of WHO's currently recommended antibiotics--systematic review and meta-analysis. Archives of disease in childhood. 2013;98(2):146-54.
15. Ronfani L, Vilarim JN, Dragovich D, Bacalhau AF, Cattaneo A. Signs of severe bacterial infection in neonates. J Trop Pediatr. 1999;45(1):48-51.
16. Arvay ML, Shang N, Qazi SA, Darmstadt GL, Islam MS, Roth DE, et al. Infectious aetiologies of neonatal illness in south Asia classified using WHO definitions: a primary analysis of the ANISA study. The Lancet Global health. 2022;10(9):e1289-e97.
17. Study IotDNI. Characterisation and antimicrobial resistance of sepsis pathogens in neonates born in tertiary care centres in Delhi, India: a cohort study. The Lancet Global health. 2016;4(10):e752-60.

18. Huynh BT, Kermorvant-Duchemin E, Chheang R, Randrianirina F, Seck A, Ratsima EH, et al. Severe bacterial neonatal infections in Madagascar, Senegal, and Cambodia: A multicentric community-based cohort study. Plos Medicine. 2021;18(9).
19. Panigrahi P, Chandel DS, Hansen NI, Sharma N, Kandefer S, Parida S, et al. Neonatal sepsis in rural India: timing, microbiology and antibiotic resistance in a population-based prospective study in the community setting. J Perinatol. 2017;37(8):911-21.
20. Polin RA. Management of neonates with suspected or proven early-onset bacterial sepsis. Pediatrics. 2012;129(5):1006-15.
21. Popescu CR, Cavanagh MMM, Tembo B, Chiume M, Lufesi N, Goldfarb DM, et al. Neonatal sepsis in low-income countries: epidemiology, diagnosis and prevention. Expert Rev Anti Infect Ther. 2020;18(5):443-52.
22. Saha SK, Schrag SJ, El Arifeen S, Mullany LC, Shahidul Islam M, Shang N, et al. Causes and incidence of community-acquired serious infections among young children in south Asia (ANISA): an observational cohort study. Lancet. 2018;392(10142):145-59.
23. Kiatchoosakun P, Jirapradittha J, Sirikarn P, Laopaiboon M, Pattanittum P, Chandrakachorn W, et al. Early-Onset Neonatal Sepsis and Antibiotic Use in Northeast Thailand. Am J Perinatol. 2019;36(12):1295-303.
24. Lavoie PM, Popescu CR, Molyneux EM, Wynn JL, Chiume M, Keitel K, et al. Rethinking management of neonates at risk of sepsis. Lancet. 2019;394(10195):279-81.
25. Iregbu K, Dramowski A, Milton R, Nsutebu E, Howie SRC, Chakraborty M, et al. Global health systems' data science approach for precision diagnosis of sepsis in early life. Lancet Infect Dis. 2022;22(5):e143-e52.

- Abstract, Page 2: Is this mortality from neonate with sepsis or others included? or does it mean 39/41 (95%) were died?

Response: We have clarified added the following clarification (lines 60 – 61): "Mortality was higher in infants with bacterial sepsis compared to other infants (22.0% [9/41] versus 8.3% [30/360]; p=0.005)." (This result was also added to the Result section (lines 246 – 247).

- How this sample was practically collected and cultured?

Response: We have added: "*Cerebrospinal fluid (CSF) cultures were collected by clinical staff, whenever they felt it was clinically indicated, at their discretion, based on availability of supplies, and the cost of the test that had to be paid by parents.*" - Note that no urine culture were performed so we deleted this and added that "*Urine cultures were not performed.*"

As for the culture methods, more information was found in section just below: "*Identification of organisms from blood or CSF cultures was completed in-house using bioMerieux API, and BD Crystal kits at a local University of North Carolina-affiliated research facility, as described (12). Bacterial isolates that could be restored (~60% of isolates) were also analyzed for further confirmation of identity on a Microflex LT MALDI-TOF mass spectrometer, using the BioTyper software version 3.1 (Bruker Daltonik, Germany).*" (lines 163 – 167).

- How this sample was determined from the beginining? Is there any clue prior knowledge for this estimation?

Response: In the parent study protocol, the main objective was to "*identify molecular RNA-sequencing signatures of bacterial sepsis in young infants with suspected sepsis (12).*" (lines 117 – 118)

In line with this original objective, the sample size was determined as follows: "*In the parent study, the sample size was set on the original molecular signature aim, at 500 infants over two years, assuming a 20% incidence of bacterial sepsis and expecting to identify between 10 and 50 differentially expressed molecular gene markers (12).*" (lines 180 – 182). Details of prior knowledge for gene markers estimates can be found in the parent study protocol publication (#12).

- In Table 1, how was "well", "ill" and "severely ill" categorized?

Response: We clarified that "*Additionally, clinical staff had to report whether they felt the infant was "well", "ill" or "severely ill" at the time of assessment. However, in keeping with a pragmatic design, the study did not attempt to enforce strict criteria for illness status, or for to define suspected sepsis or the presence or absence of DS.*" (lines 152 – 155)

- Regarding the association with "not feeding well" and bacterial sepsis, what this indicates, or could you recommend this DS for empiric diagnosis of bacterial sepsis?

Response: We added to the discussion: "*Notably, a significant minority (39%) of parent of infants with bacterial sepsis initially declared that their child was feeding well at presentation. Therefore, absence of this DS could practically miss a large proportion of infants with bacterial sepsis in real life.*" (lines 278 – 281)

- Request to bold or add (*) beside significant p values in Tables.

Response: We have made these changes in the main tables and supplemental tables.

- Could there a possibility that some of the contaminants are contributing sepsis? How contaminant was defined in the study?

Response: Thank you for this great question. We are aware that many expert believe that positive blood cultures for certain bacteria should be considered as possible contaminants systematically. Anticipating this, since this is not universally agreed upon in the literature, we have conducted the sensitivity analysis where we include those bacterial cultures in the (modified) "sepsis" definition. So we believe that this reviewer's question is best addressed by this analysis.

Regarding the definition of potential contaminants, we asked our microbiological experts and based on their clinical expertise (and their knowledge of the literature), they have listed the following commonly considered bacterial contaminants (as detailed in method section): "*Micro-organisms not commonly considered as pathogens in term or near-term infants, such as coagulase-negative Staphylococcus, Micrococcus, Corynebacterium, Bacillus species, or streptococcus species such as S. oralis were considered as potential contaminants and were excluded from the main bacterial sepsis outcome (14), but these potential contaminants were included in a sensitivity analysis using an alternate bacterial sepsis outcome "including potential contaminants" (Supplemental Table 1).*" (lines 171 – 176). We have also grouped and reorganize results to make this more clear (lines 262 – 269)

The list of those bacteria considered as potential contaminants is also in Supplemental Table 1, so we have modified the corresponding Result paragraph to include reference to this Table (line 263)

- Do you mean including both pathogen and contaminants? specify? what is the rate of contaminants in overall the study? How do you compare it with the WHO contaminant rate for validating the blood culture?

Response: Yes, we have now clarified: "*The number of DS was also not significantly associated with bacterial sepsis when including all pathogens and potential contaminants (OR 1.09 [95% CI: 0.93 – 1.28]; p=0.273; Supplemental Figure 3).*" (lines 269 – 271)

Apologies, we are not aware of a WHO definition of contaminants so we are unable to answer the question regarding this…

- Adding reference to rural settings in Bangladesh, India and Pakistan.

Response: The reference was #16. We have moved it earlier in the sentence to make this clear (line 286)

- Reference to culture positive sepsis in ref #17 similar to current study lacking statistical test.

Response: Thank you for this question. What we meant here is that results in these cited studies are in keeping with the current study findings (line 307) i.e., only a minority of infants with suspected bacterial sepsis actually had proven positive blood culture. We clarified that "In addition to the main findings, the current study also found that only a minor proportion of infants with suspected sepsis showed positive blood culture." (lines 301-302). We did not mean to make any statistical inference here.

ADDITIONAL QUESTIONS:

#1. How were the study participants selected? What was the criteria included in the study? How was sepsis suspected was defined for this study?

Response: We re-iterated that "*In the parent study, infants below 3 months of age with suspected sepsis, defined according to the attending staff's clinically suspicion…*" (lines 118 – 119)

#2. Why high figure of non-response rate (401/851 approached)? Was it the problem of awareness or high price of blood culture? How do you explain this for researchers.

Response: Most babies were missed because "*parents either declined, or were not approached for consent due to lack of time and/or competing clinical demands.*", as mentioned on (lines 125 – 126) Patients did not have to pay for the blood cultures, which were standard and paid for infants in the study. This was indicated in the original study protocol (ref #12) and is now clarified (line 162): "*Blood cultures, were paid by the study…*". This sort of non-response rate is not unusual in a study setting, particularly considering this study was done is a very limited, difficult, LMIC setting.

#3. Clarify how you had determined the sample size in your plan (500) and achievement (400).

Response: Thank you for this question. As mentioned above, the sample size was determined as follows: "*In the parent study, the sample size was set on the original molecular signature aim, at 500 infants over two years, assuming a 20% incidence of bacterial sepsis and expecting to identify between 10 and 50 differentially expressed molecular gene markers (12).*" (lines 180 – 182). Details of prior knowledge for gene markers estimates can be found in the parent study protocol publication (#12).

#4. In page 6 line 155 you mentioned about urine culture in your work but can't found any in the finding or method related with this. Clarify!

Response: Urine cultures were allowed in the original study, but not provided by the study. In the end, no urine cultures were performed. This has been clarified: "*Cerebrospinal fluid (CSF) cultures were collected by clinical staff, whenever they felt it was clinically indicated, at their discretion, based on availability of supplies, and the cost of the test that had to be paid by parents. No urine cultures were performed.*" (lines 158 – 160) This has also been added to the limitations (line 339)

#5. You did CSF culture for the selected infants in the study. How did you select for those who CSF culture done and not done?

Response: Unfortunately, CSF cultures were not enforced (contrary to blood cultures which were enforced, with standardized training of all staff for aseptic technique at the beginning of the study, and supplies / cost of the blood culture processing paid for, and processed in a certified NIH lab on-site, as indicated in original study protocol and lines 155 – 158), so because of this CSF cultures were not systematically done.

#6. Most of children was on antibiotics for the median?? Of 6 days. How this matched with your mention exclusion criteria, more than 4hrs? May the BACTEC have an advantage of reducing the effect of antibiotics on bacterial growth?

Response: Thank you for this question. The max 4h was the time infants were on antibiotics for before the blood culture. The median of 6 days was the total duration of antibiotics once cultured and the antibiotics were initiated. We added "*total*" in the corresponding line in Table 1, and "*that infants were excluded if they had been on antibiotics more than 4 hours prior to the cultures*", hoping this clarifies.

#7. How were contaminants defined in the study? Have you done with more than one bottle blood culture? Is there a possibility for those bacteria like CoNS, to be a potential pathogen?

Response: See response to similar question above. Additionally, only one blood culture was sent per child (as it is the standard of care in most settings). Based on many experts, CoNS is not a pathogen in late gestation/term infants who do not have central lines. However, because this could be debated we have planned to do the sensitivity analysis including all positive blood cultures (with similar results).

#8. What is the total contaminant rate in the study based on your definition used? How this rate comparable with the tolerable WHO blood culture rate? Figure on the supplementary table is high!

Response: We agree that the proportion of contaminants can be viewed as high. Despite rigorous in-training of all clinical staff at the beginning of the study (by our own nurses and physicians from Canada), for the aseptic technique, we are left believing that in best conditions in LMICs many blood cultures may be contaminated. This is a potential indirect lesson from our study and experience in this setting. Alternatively, it remains possible that a higher proportion of infants with IV catheters become infected with bacterial that would otherwise be considered as contaminants in a North American setting. However, we think it is extremely unlikely that many would show up on the initial blood culture (i.e. before the IV cannula is inserted). Therefore, we have not discussed this as we think the most likely possibility is that it is the cultures themselves that were contaminated despite the standardized guidelines and procedures we tried to implement in this unit. This could be studied outside this article.

#9. Once you have an identified bacterium, why can't look for antibiotic resistance?

Response: We have obtained resistance data, and all isolates were shipped to Canada for sequencing. These results are being analyzed, but outside the scope of this publication.

#10. What is the overall about the conclusion of your study? Does it conclude with DS more related with sepsis caused by other than bacterial infections or what? elaborate?

Response: Thank you for asking clarification on this. The main interpretation we make of the study results is that *"…DS predict fatal outcomes, but not to reliably identify infants with bacterial sepsis. In other words, relying solely on DS to identify infants with bacterial sepsis is likely to lead to overtreatment while at the same time missing infants who require antibiotic treatment."* (lines 349 – 354)

In addition, as stated in the Abstract: "*DS predicted death, but was marginally associated with culture-positive pathogen sepsis in a regional hospital setting. These data imply that the incidence of bacterial sepsis and attributable mortality in infants based on clinical signs may not be accurate, in LMIC settings.*" (lines 70 – 72)

We also made a few minor edits throughout the manuscript for clarity (see tracked changes).
