# Supplementary material for: World Health Organization Danger Signs to predict bacterial sepsis in young infants: A pragmatic cohort study
Source: PLOS Glob Public Health. 2023 Nov 21;3(11):e0001990. doi: 10.1371/journal.pgph.0001990 (PMC10662722; doi:10.1371/journal.pgph.0001990)
Supplement: Attachment — Submitted filename: Plos Response to Reviewers comments.docx [file pgph.0001990.s010.docx]

General comment
• In the main method give the operation definition ‘suspected sepsis’ in newborns.

The definition of “suspected sepsis” was completely up to the attending staff, as it occurs pragmatically in the clinical setting. The following clarification has been made (line 108-110): This study was a pragmatic cohort study, and secondary analysis of data from a parent study that aimed to identify molecular signatures of bacterial sepsis in young infants with suspected sepsis, defined according to the attending staff’s suspicion (12)."

We also added (line 149)): “In keeping with a pragmatic design, the study did not attempt to enforce strict criteria to define suspected sepsis, DS or illness status.”

• Which types of bacteria were isolated from CSF?

As explained above, the two infants with positive CSF results had been mistakenly omitted, so this is now corrected in the revised manuscript. One child grew klebsiella pneumonia in the CSF (with a negative blood culture – was previously classified as such but is now reclassified as positive) and the other grew a coagulase-negative staph in the blood (previously classified as “contaminant”) but grew an unidentified gram-negative diplococci in the CSF (that we now consider and reclassify as pathogen).

The analysis was revised accordingly, with minor changes in numbers, no major changes in overall conclusions or data interpretation.

• Some terms are inconsistent within the manuscript “young infants”, “Newborns”, “Neonates”, “infants” It is good to define the study population and use it consistently throughout the manuscript.

Replaced newborns and neonates with infants.

• The study design, sample size (500 vs 401 vs) is not clearly mentioned.

We have clarified that: “In the parent study, the sample size was set conveniently at 500 infants over 2 years, but the study needed to be stopped in April 2020 due to the COVID-19 pandemic, so 401 infants were enrolled and included in the study.”

• Consider replacing ‘h’ with ‘hours’ if it means so.

Line 61 and 212- 48h changed to 48 hours.

• Line # 144 “enrollment” Line # “approximatively” check the spelling.

Thank you for this comment, language was made uniform throughout in Canadian/British English (e.g., enrollment, instead of enrolment) as we were unable to find guidance on this on the PloS site.

• Mention the nine WHO danger signs in the clinical data and samples collection section.

We have added the “nine” to the objective line of the abstract. Most relevant to your point, we have clearly listed (and numbered) the nine DS in the Clinical data and blood sample collection Method section: “The presence of the following nine DS were prospectively collected at presentation, using electronic forms, on a tablet device: i) history of not feeding well (interviewing the parent/caregiver), entered as “feeding well”, “unable to feed” or “uninterested in feeding” (combining the latter two categories), ii) fast breathing ≥60 bpm (with respiratory rate visually counted by attended clinician), iii) temperature instability, defined as axillary body temperature <35.5°C or >38°C, (measured using an electronic Welch Allyn SureTemp thermometer, model 692), iv) drowsiness/difficulty to wake, v) grunting, vi) severe chest recession, vii) central cyanosis, viii) movements only when stimulated or no movement at all and ix) convulsions (defined pragmatically by attending clinician).”

• Was the attempt made to isolate anaerobic bacteria? If not. include in the limitation.

No, we have not done anaerobic blood cultures – this has been added as a limitation.

• As the etiology of neonatal sepsis varies with age; I suggest considering presenting the result section by taking into account the age classification of newborns (< 7 days of age and 7-90 days of age).

We thank the reviewer for this suggestion. The numbers are small, but for whatever it is worth, we have added the observations that: “Of 230 infants under 7 days old, 21 (9.1%) had bacterial sepsis with a pathogen versus 20 of the 171 infants (11.7%; p=0.402 by chi-square test) ≥ 7 days old. The proportion of gram-positive sepsis (21/39) and gram-negative sepsis (20/39) were similar among all infants (**Supplemental Table 1**), and were also similar between infants under 7 days (9 versus 12) and infants ≥ 7 days old (12 versus 8; p=0.272 by chi-square test, noting however the small sample size for comparison).“

• What were the criteria to collect CSF?

It was left up to the discretion of the treating physician. “Infants also had cerebrospinal fluid (CSF) and urine cultures collected at the discretion of the clinical staff, availability of resources and cost of the test to be paid by parents.” Line 146 and 147.

• How bacteria were identified after blood culture is not clear, for how long the blood culture was incubated before it was declared negative. There is no description of how CSF was cultured.

Blood cultures were incubated for 5 days before it was declared negative (which is fairly standard across microbiology lab in North America). We have added this (line 49).

“Identification of organisms from blood or CSF cultures was completed in-house using bioMerieux API, and BD Crystal kits at a local University of North Carolina-affiliated research facility, as described (12). Bacterial isolates that could be restored (∼60% of isolates) were also analyzed for further confirmation of identity (not shown) on a Microflex LT MALDI-TOF mass spectrometer, using the BioTyper software version 3.1 (Bruker Daltonik, Germany).”

Specific comments
• In the abstract section, I suggest separating methods and findings/results.

Done line 59.

• In the abstract it is written as “This prospective study evaluated nine DS in infants younger than 3 months…” Do newborns with the age of 3 months were included? **The comment is unclear to me.**

Hoping we understood well the reviewer’s comment, we have clarified in the manuscript that the study enrolled infants under 3 months of age, including newborns, and used “infant” throughout.

• Line #93: “Over time, the use of DS has been extended to provide a reasonable basis for the initiation of empiric antibiotic treatment (9). However, these signs have neither been developed nor validated to diagnose sepsis of bacterial etiology.” Is it correct to use reference #9 for the information given? Reference #9 looks like a guideline developed by WHO, is the WHO recommending the use of empiric antibiotic treatment without evidence?

Yes, the WHO is recommending to start antibiotics in a child that is showing DS, which was a major impetus for conducting this study. This has been extensively discussed in the literature and it is based on the rationale that, though these signs may be non-specific, starting antibiotics early in the children who truly have bacterial sepsis could be life-saving.

• In material and method section line #109 “study that aimed to identify molecular signatures of bacterial sepsis in young infants…” was molecular techniques used to diagnose sepsis?

Mainly RNA-sequencing (has been added to the sentence).

• Line #113-114: “…. infants were excluded if they had received antibiotics for >4 hours at the time of enrollment.” What if they have received antibiotics treatment in the last two weeks?

The sentence has been reworded to clarify: “infants were excluded if they were already on antibiotics for >4 hours…”

• Line # 114-117: Out of 851, how many refused to participate, the condition was not permissive, and how many were included in the study?

Unfortunately, we do not have data on how many declined versus were not approached for consent.

• Line #119: “The Kamuzu Central Hospital the largest referral hospital in central Malawi, where approximatively 3,000 infants are admitted to its neonatal unit annually” needs reference

We are unaware of a published document we can reference for this. Admittedly, statistics in a poor country like Malawi are much less formally documented than can be in high income countries.

• Line # 128-129: “During the study, the authors did not have access to information that could identify participants…” if this is the case how lab results were communicated to the attending physician?

The blood culture results were communicated in real-time to attending staff but were not shared with the researchers. The sentence has been reworded to : “During the study, investigators did not have access to information that could identify participants, with the exception of the consent forms kept under the responsibility of the local study PI (MCK).”

Line # 150 “Culture results were made available to clinicians within 5 days.” And also the recommended incubation period of blood culture is about a week, how culture result is available for the physician within 5 days?

Actually, we truly we did not capture the time between blood culture and resulting to clinicians, so more appropriately, the sentence has been reworded to: Culture results were made available to clinicians involved in the care of the infants.”

• Line # 193-195: “In total, 94/401 (23.4%) infants had received antibiotics prior to the blood culture, for a median of 2 hours (range 0.75 to 3.5 195 hours)” This could have affected the findings.

We suspect that this would be unlikely to negate blood culture results in most cases. However, fairly, we have added the limitation that: “Some infants received antibiotics prior to blood culture, which may have lowered bacterial blood estimates.” Our sense also is that many other studies had a similar problem but we were unable to find report of this information in most of these studies.

• Some data were collected from the baby’s mother for example gestational age, and mode of delivery. Was consent sought from mothers?

Yes, consent was obtained from the mothers to obtain these data.

• Table 1 needs to be improved. Why values in the column ‘N with data’ are variable given the total sample size is 401? Fifth row what does ‘inborn’ mean?

Inborn means that the child was admitted from the birth hospital (i.e., never went home after delivery). As for the N with data, it is good practice to report missing data for every variable. In reality, all studies have missing data. The N refers to the number of babies with data for each specific variable.

• Table 1: Why capturing “Illness status, number on infants” is needed provided that the study population are newborns suspected of sepsis (ill) visiting the hospital.

Not all infants were ill at the time of initiation of antibiotics. Some were showing obviously some symptoms but were deemed general “well” by the attending staff (i.e., fever in an otherwise well baby).

• Line # 202: The contamination rate is somewhat high “However, about half (46 / 85) had bacterial types considered to be contaminants” If the blood specimen were collected aseptically they may be true pathogens.

Even in the best conditions when an aseptic technique is supposed to be used, would contaminants be expected even in a child with suspected sepsis. The literature often considers some bacteria very likely to be contaminants in a near term/term baby without risk factors. This is debatable but is what we have discussed with our microbiologist experts. It is also why we conducted the sensitivity analysis with all blood culture (a recognizable strength in our study).

• Line 203-204: “…and non-pathogenic streptococcus species such as oralis)…” you may rewrite as ‘….and non-pathogenic Streptococcus species such as S. oralis)…’ Consider scientific name during writing the name of bacteria.

Thank you, we have made this correction.

• Table 2: it is good if a column is added to show frequency and proportion in the ‘yes’ and ‘No’ categories for all variables. Doing so will make the reader see the prevalence in each cell.

• Table 3: it is good if a column is added to show frequency and proportion in yes and No category for all variables.

We thank the reviewer for these two suggestions, but the data is already in Table 1. Would it need to be repeated for each table? We hope this is acceptable to the reviewer, for the sake of simplicity.

• Tables 2 and 3: I don’t see the importance of using (), it can be omitted.

Change made

• Discussion lines # 277-278: “Taken together, these findings suggest that bacterial sepsis may be uncommon in this age group even in LMICs when sepsis is clinically suspected.” This is an area of controversy in LMICs and sub-Saharan African countries. Proxy studies and systematic reviews and meta-analyses reported a high burden of neonatal sepsis in these regions. Strong recommendations should be forwarded to concerned bodies so that similar studies (like the present study) using robust diagnostic methods to be conducted in the region. This will assist in device appropriate prevention strategies for the regions which may include vaccines and measure the impact of the prevention strategy. Without knowing the real burden of neonatal sepsis along with its etiology we may not objectively measure the impact of the prevention strategy to be used.

We thank the reviewer for this comment. We agree that it could be controversial to qualify the incidence as being “low” (or not). Based on the reviewer’s suggestion we have reworded the sentence: Taken together, these findings provide robust data on the incidence of bacterial sepsis in LMIC settings, to inform interventions and form the basis for future studies to measure the impact of those interventions.”

• Discussion: line# 302-304: “The strong association between bacterial sepsis and mortality suggest that the infants with positive cultures were correctly identified” Is there a finding which supports this claim?

What we mean here is that if the bacterial cultures were completely non-specific, we would not expect a difference in mortality between culture positive and culture-negative children. Perhaps it is clearly now when reworded as: The strong association between bacterial sepsis and mortality provides reassurance infants with positive cultures were correctly identified.”
