# Supplementary material for: World Health Organization Danger Signs to predict bacterial sepsis in young infants: A pragmatic cohort study
Source: PLOS Glob Public Health. 2023 Nov 21;3(11):e0001990. doi: 10.1371/journal.pgph.0001990 (PMC10662722; doi:10.1371/journal.pgph.0001990)
Supplement: Attachment — Submitted filename: Response to reviewers.docx [file pgph.0001990.s012.docx]

- Abstract, Page 2: Is this mortality from neonate with sepsis or others included? or does it mean 39/41 (95%) were died?

**Response:** We have clarified added the following clarification (lines 60 – 61): “Mortality was higher in infants with bacterial sepsis compared to other infants (22.0% [9/41] versus 8.3% [30/360]; p=0.005).” (This result was also added to the Result section (lines 246 – 247).

- How this sample was practically collected and cultured?

**Response:** We have added: “*Cerebrospinal fluid (CSF) cultures were collected by clinical staff, whenever they felt it was clinically indicated, at their discretion, based on availability of supplies, and the cost of the test that had to be paid by parents.*” - Note that no urine culture were performed so we deleted this and added that “*Urine cultures were not performed.*”

As for the culture methods, more information was found in section just below: “*Identification of organisms from blood or CSF cultures was completed in-house using bioMerieux API, and BD Crystal kits at a local University of North Carolina-affiliated research facility, as described (12). Bacterial isolates that could be restored (∼60% of isolates) were also analyzed for further confirmation of identity on a Microflex LT MALDI-TOF mass spectrometer, using the BioTyper software version 3.1 (Bruker Daltonik, Germany).*” (lines 163 – 167).

- How this sample was determined from the beginining? Is there any clue prior knowledge for this estimation?

**Response:** In the parent study protocol, the main objective was to “*identify molecular RNA-sequencing signatures of bacterial sepsis in young infants with suspected sepsis (12).*” (lines 117 – 118)

In line with this original objective, the sample size was determined as follows: “*In the parent study, the sample size was set on the original molecular signature aim, at 500 infants over two years, assuming a 20% incidence of bacterial sepsis and expecting to identify between 10 and 50 differentially expressed molecular gene markers (12).*” (lines 180 – 182). Details of prior knowledge for gene markers estimates can be found in the parent study protocol publication (#12).

- In Table 1, how was “well”, “ill” and “severely ill” categorized?

**Response:** We clarified that “*Additionally, clinical staff had to report whether they felt the infant was “well”, “ill” or “severely ill” at the time of assessment. However, in keeping with a pragmatic design, the study did not attempt to enforce strict criteria for illness status, or for to define suspected sepsis or the presence or absence of DS.*” (lines 152 – 155)

- Regarding the association with “not feeding well” and bacterial sepsis, what this indicates, or could you recommend this DS for empiric diagnosis of bacterial sepsis?

**Response:** We added to the discussion: “*Notably, a significant minority (39%) of parent of infants with bacterial sepsis initially declared that their child was feeding well at presentation. Therefore, absence of this DS could practically miss a large proportion of infants with bacterial sepsis in real life.*” (lines 278 – 281)

- Request to bold or add (*) beside significant p values in Tables.

**Response:** We have made these changes in the main tables and supplemental tables.

- Could there a possibility that some of the contaminants are contributing sepsis? How contaminant was defined in the study?

**Response:** Thank you for this great question. We are aware that many expert believe that positive blood cultures for certain bacteria should be considered as possible contaminants systematically. Anticipating this, since this is not universally agreed upon in the literature, we have conducted the sensitivity analysis where we include those bacterial cultures in the (modified) “sepsis” definition. So we believe that this reviewer’s question is best addressed by this analysis.

Regarding the definition of potential contaminants, we asked our microbiological experts and based on their clinical expertise (and their knowledge of the literature), they have listed the following commonly considered bacterial contaminants (as detailed in method section): “*Micro-organisms not commonly considered as pathogens in term or near-term infants, such as coagulase-negative Staphylococcus, Micrococcus, Corynebacterium, Bacillus species, or streptococcus species such as S. oralis were considered as potential contaminants and were excluded from the main bacterial sepsis outcome (14), but these potential contaminants were included in a sensitivity analysis using an alternate bacterial sepsis outcome “including potential contaminants” (Supplemental Table 1).*” (lines 171 – 176). We have also grouped and reorganize results to make this more clear (lines 262 – 269)

The list of those bacteria considered as potential contaminants is also in Supplemental Table 1, so we have modified the corresponding Result paragraph to include reference to this Table (line 263)

- Do you mean including both pathogen and contaminants? specify? what is the rate of contaminants in overall the study? How do you compare it with the WHO contaminant rate for validating the blood culture?

**Response:** Yes, we have now clarified: “*The number of DS was also not significantly associated with bacterial sepsis when including all pathogens and potential contaminants (OR 1.09 [95% CI: 0.93 – 1.28]; p=0.273; Supplemental Figure 3).*” (lines 269 – 271)

Apologies, we are not aware of a WHO definition of contaminants so we are unable to answer the question regarding this…

- Adding reference to rural settings in Bangladesh, India and Pakistan.

**Response:** The reference was #16. We have moved it earlier in the sentence to make this clear (line 286)

- Reference to culture positive sepsis in ref #17 similar to current study lacking statistical test.

**Response:** Thank you for this question. What we meant here is that results in these cited studies are in keeping with the current study findings (line 307) i.e., only a minority of infants with suspected bacterial sepsis actually had proven positive blood culture. We clarified that “In addition to the main findings, the current study also found that only a minor proportion of infants with suspected sepsis showed positive blood culture.” (lines 301-302). We did not mean to make any statistical inference here.

ADDITIONAL QUESTIONS:

#1. How were the study participants selected? What was the criteria included in the study? How was sepsis suspected was defined for this study?

**Response:** We re-iterated that “*In the parent study, infants below 3 months of age with suspected sepsis, defined according to the attending staff’s clinically suspicion…*” (lines 118 – 119)

#2. Why high figure of non-response rate (401/851 approached)? Was it the problem of awareness or high price of blood culture? How do you explain this for researchers.

**Response:** Most babies were missed because “*parents either declined, or were not approached for consent due to lack of time and/or competing clinical demands.*”, as mentioned on (lines 125 – 126) Patients did not have to pay for the blood cultures, which were standard and paid for infants in the study. This was indicated in the original study protocol (ref #12) and is now clarified (line 162): “*Blood cultures, were paid by the study…*”. This sort of non-response rate is not unusual in a study setting, particularly considering this study was done is a very limited, difficult, LMIC setting.

#3. Clarify how you had determined the sample size in your plan (500) and achievement (400).

**Response:** Thank you for this question. As mentioned above, the sample size was determined as follows: “*In the parent study, the sample size was set on the original molecular signature aim, at 500 infants over two years, assuming a 20% incidence of bacterial sepsis and expecting to identify between 10 and 50 differentially expressed molecular gene markers (12).*” (lines 180 – 182). Details of prior knowledge for gene markers estimates can be found in the parent study protocol publication (#12).

#4. In page 6 line 155 you mentioned about urine culture in your work but can’t found any in the finding or method related with this. Clarify!

**Response:** Urine cultures were allowed in the original study, but not provided by the study. In the end, no urine cultures were performed. This has been clarified: “*Cerebrospinal fluid (CSF) cultures were collected by clinical staff, whenever they felt it was clinically indicated, at their discretion, based on availability of supplies, and the cost of the test that had to be paid by parents. No urine cultures were performed.*” (lines 158 – 160) This has also been added to the limitations (line 339)

#5. You did CSF culture for the selected infants in the study. How did you select for those who CSF culture done and not done?

**Response:** Unfortunately, CSF cultures were not enforced (contrary to blood cultures which were enforced, with standardized training of all staff for aseptic technique at the beginning of the study, and supplies / cost of the blood culture processing paid for, and processed in a certified NIH lab on-site, as indicated in original study protocol and lines 155 – 158), so because of this CSF cultures were not systematically done.

#6. Most of children was on antibiotics for the median?? Of 6 days. How this matched with your mention exclusion criteria, more than 4hrs? May the BACTEC have an advantage of reducing the effect of antibiotics on bacterial growth?

**Response:** Thank you for this question. The max 4h was the time infants were on antibiotics for before the blood culture. The median of 6 days was the total duration of antibiotics once cultured and the antibiotics were initiated. We added “*total*” in the corresponding line in Table 1, and “*that infants were excluded if they had been on antibiotics more than 4 hours prior to the cultures*”, hoping this clarifies.

#7. How were contaminants defined in the study? Have you done with more than one bottle blood culture? Is there a possibility for those bacteria like CoNS, to be a potential pathogen?

**Response:** See response to similar question above. Additionally, only one blood culture was sent per child (as it is the standard of care in most settings). Based on many experts, CoNS is not a pathogen in late gestation/term infants who do not have central lines. However, because this could be debated we have planned to do the sensitivity analysis including all positive blood cultures (with similar results).

#8. What is the total contaminant rate in the study based on your definition used? How this rate comparable with the tolerable WHO blood culture rate? Figure on the supplementary table is high!

**Response:** We agree that the proportion of contaminants can be viewed as high. Despite rigorous in-training of all clinical staff at the beginning of the study (by our own nurses and physicians from Canada), for the aseptic technique, we are left believing that in best conditions in LMICs many blood cultures may be contaminated. This is a potential indirect lesson from our study and experience in this setting. Alternatively, it remains possible that a higher proportion of infants with IV catheters become infected with bacterial that would otherwise be considered as contaminants in a North American setting. However, we think it is extremely unlikely that many would show up on the initial blood culture (i.e. before the IV cannula is inserted). Therefore, we have not discussed this as we think the most likely possibility is that it is the cultures themselves that were contaminated despite the standardized guidelines and procedures we tried to implement in this unit. This could be studied outside this article.

#9. Once you have an identified bacterium, why can’t look for antibiotic resistance?

**Response:** We have obtained resistance data, and all isolates were shipped to Canada for sequencing. These results are being analyzed, but outside the scope of this publication.

#10. What is the overall about the conclusion of your study? Does it conclude with DS more related with sepsis caused by other than bacterial infections or what? elaborate?

**Response:** Thank you for asking clarification on this. The main interpretation we make of the study results is that *“…DS predict fatal outcomes, but not to reliably identify infants with bacterial sepsis. In other words, relying solely on DS to identify infants with bacterial sepsis is likely to lead to overtreatment while at the same time missing infants who require antibiotic treatment.*” (lines 349 – 354)

In addition, as stated in the Abstract: “*DS predicted death, but was marginally associated with culture-positive pathogen sepsis in a regional hospital setting.* *These data imply that the incidence of bacterial sepsis and attributable mortality in infants based on clinical signs may not be accurate, in LMIC settings.*” (lines 70 – 72)

We also made a few minor edits throughout the manuscript for clarity (see tracked changes).
